# Dual Substrate Specificity of the Rutinosidase from *Aspergillus niger* and the Role of Its Substrate Tunnel

**DOI:** 10.3390/ijms21165671

**Published:** 2020-08-07

**Authors:** Katerina Brodsky, Michal Kutý, Helena Pelantová, Josef Cvačka, Martin Rebroš, Michael Kotik, Ivana Kutá Smatanová, Vladimír Křen, Pavla Bojarová

**Affiliations:** 1 Institute of Microbiology of the Czech Academy of Sciences, Vídeňská 1083, CZ 14220 Prague 4, Czech Republic; katerina.brodsky@biomed.cas.cz (K.B.); pelantova@biomed.cas.cz (H.P.); kotik@biomed.cas.cz (M.K.); kren@biomed.cas.cz (V.K.); 2 Department of Biochemistry and Microbiology, University of Chemistry and Technology Prague, Technická 3, CZ 16628 Prague 6, Czech Republic; 3 Center for Nanobiology and Structural Biology, Institute of Microbiology of the Czech Academy of Sciences, Zámek 136, CZ 37333 Nové Hrady, Czech Republic; kutym@seznam.cz (M.K.); ivanaks@seznam.cz (I.K.S.); 4 Institute of Chemistry, Faculty of Science, University of South Bohemia, Branišovská 1760, CZ 37005 České Budějovice, Czech Republic; 5 Institute of Organic Chemistry and Biochemistry of the Czech Academy of Sciences, Flemingovo nám. 2, CZ 16610 Prague 6, Czech Republic; josef.cvacka@uochb.cas.cz; 6 Institute of Biotechnology, Slovak University of Technology, Radlinského 9, SK 81237 Bratislava, Slovakia; martin.rebros@stuba.sk

**Keywords:** azide, glycosylation, molecular modeling, rutinosidase, tunnel

## Abstract

Rutinosidases (α-l-rhamnopyranosyl-(1-6)-β-d-glucopyranosidases, EC 3.2.1.168, CAZy GH5) are diglycosidases that cleave the glycosidic bond between the disaccharide rutinose and the respective aglycone. Similar to many retaining glycosidases, rutinosidases can also transfer the rutinosyl moiety onto acceptors with a free –OH group (so-called transglycosylation). The recombinant rutinosidase from *Aspergillus niger* (*An*Rut) is selectively produced in *Pichia pastoris.* It can catalyze transglycosylation reactions as an unpurified preparation directly from cultivation. This enzyme exhibits catalytic activity towards two substrates; in addition to rutinosidase activity, it also exhibits β-d-glucopyranosidase activity. As a result, new compounds are formed by β-glucosylation or rutinosylation of acceptors such as alcohols or strong inorganic nucleophiles (NaN_3_). Transglycosylation products with aliphatic aglycones are resistant towards cleavage by rutinosidase, therefore, their side hydrolysis does not occur, allowing higher transglycosylation yields. Fourteen compounds were synthesized by glucosylation or rutinosylation of selected acceptors. The products were isolated and structurally characterized. Interactions between the transglycosylation products and the recombinant *An*Rut were analyzed by molecular modeling. We revealed the role of a substrate tunnel in the structure of *An*Rut, which explained the unusual catalytic properties of this glycosidase and its specific transglycosylation potential. *An*Rut is attractive for biosynthetic applications, especially for the use of inexpensive substrates (rutin and isoquercitrin).

## 1. Introduction

Rutinosidases (6-α-l-rhamnopyranosyl-β-d-glucopyranosidases, EC 3.2.1.168) are diglycosidases that cleave rutinosides, affording the respective aglycone and the disaccharide rutinose [1]. Rutinosidases are commonly found in plants [2] and microorganisms [3,4] and their natural substrates are flavonoids such as rutin or hesperidin. Rutinosidase is used in the food industry for taste and aroma improvement of tea or wine or for citrus juice debittering [5]. Quercetin and hesperetin have been proven to have anti-inflammatory and antioxidative effects [6,7,8,9]. Rutinosidases can be applied in the manufacturing of dietary supplements and bioactive products that contain these flavonoids. The production process becomes more efficient because rutinosidases cleave flavonoid glycosides in a single step [10]. The substrates of *An*Rut are mostly by-products of the food industry, which makes them both inexpensive and eco-friendly [11]. The released disaccharide rutinose is currently being investigated as a prospective cosmetic and medicinal agent for its potential antiaging capability [12].

One of the most studied rutinosidases is the rutinosidase from *Aspergillus niger* K2 (*An*Rut), which is also the first heterogously (*Pichia pastoris*) produced enzyme of this class [13]; it is a member of the glycoside hydrolase family GH-5, subfamily 23 (http://www.cazy.org/) [14]. In addition to its hydrolytic activity, *An*Rut is also able to transfer the disaccharide moiety onto acceptors such as alcohols, phenolic acceptors, and even carboxylic acids [13,15]. This process, which is common among retaining glycosidases [16], is called transglycosylation. The substrate for transglycosylation, i.e. rutin, is an inexpensive bulk substance produced in food-grade quality. Quercetin, which is the aglycone released from this reaction, can be easily removed by filtration. The reaction proceeds even at a high rutin concentration in suspension, in the mode of the so-called “solid state biocatalysis” [17].

It has recently been found [18] that *An*Rut has dual substrate specificity, processing the diglycosidic substrate rutin (quercetin-3-*O*-rutinoside) and also the monoglycosidic isoquercitrin (quercetin-3-*O*-β-d-glucopyranoside). The recently published crystal structure of *An*Rut (Protein data bank, PDB ID: 6I1A) revealed that the active site of this enzyme was strictly specific for both the aglycone (quercetin) and the β-glucosyl moiety of the substrate (e.g., isoquercitrin) but surprisingly less specific towards the terminal rhamnosyl moiety of the diglycosidic substrate rutin [18]. The *An*Rut enzyme is a typical retaining glycosidase, which employs the two-step double-displacement retaining reaction mechanism, as previously described in detail [18].

The major biotechnological value of *An*Rut lies in its particular transglycosylation potential. Rutinosylated or glucosylated compounds find their use in biomedical and biological applications [19]. Rhamnosyl rich oligo- and polysaccharides have been proven to be active in inhibiting the harmful effects of advanced glycation end-products (AGEs) such as cytotoxicity. AGEs are nonspecifically glycated proteins decreasing cellular efficiency that originate during aging. AGEs have been shown to catalyze elastase-type endopeptidase expression in human skin fibroblasts [20,21]. Rhamnose only occurs in plants and microorganisms but not in higher organisms (i.e., humans). To exert its anti-AGE effect, this carbohydrate should be delivered to human cells by a carrier, therefore, *An*Rut is a promising tool for the preparation of multivalent rhamnose-carrying neoglycoconjugates, as also demonstrated in this work. The ability to efficiently glycosylate a range of substrates of various structural features with a mono- or a disaccharide moiety makes *An*Rut a useful tool for medical, pharmaceutic, and food industry applications.

In this paper we describe a series of novel transglycosylation products prepared using *An*Rut, carrying either a β-glucopyranosyl or rutinosyl moiety. Both rutin and isoquercitrin were used as glycosyl donors in transglycosylation reactions, demonstrating the glycosylation capability of *An*Rut with these two substrates. A possible application of the prepared transglycosylation products was shown in the synthesis of a trivalent glycoconjugate by the “click” chemistry method. Important structure-affinity aspects within the analyzed acceptor series were found using molecular modeling. We revealed the existence and function of a large substrate tunnel leading to the active site, which was unusual among glycosidases [22,23]. We discussed our finding in relation to other glycosidases structurally close to *An*Rut [24,25]. In silico modeling shed light on the specific structural relations in the active site of *An*Rut and revealed crucial enzyme-substrate interactions during the catalytic process.

## 2. Results

### 2.1. Production of Recombinant Rutinosidase from A. niger

The large-scale production of recombinant *An*Rut by *P. pastoris* KM71H was performed in 3 L laboratory bioreactors, as described previously [17]. The overall yield of the *An*Rut fermentation was 5.7 mg protein per liter per hour. The recombinant *An*Rut was produced virtually as a sole protein during fermentation and it had a molecular mass of ca 66 kDa, as determined by SDS-PAGE. The crude enzyme was stable for several months; only after a longer period of time the enzyme was partially deglycosylated, showing a band of ca 45 kDa on SDS electrophoresis, which agreed with previous results [13] (see the Supplementary Figure S1). The enzyme activity was determined using *p-*nitrophenyl rutinoside (**15**) to be 0.81 U/mL and the specific activity was 1.14 U/mg. It was found by means of size exclusion chromatography that the native deglycosylated enzyme occurred as a dimer in solution (see the Supplementary Figure S2); its molecular mass corresponded to ca 87.1 kDa, which was in accordance with the estimated molecular mass of a dimer (90 kDa).

### 2.2. Transglycosylation Products

**Figure 1:**
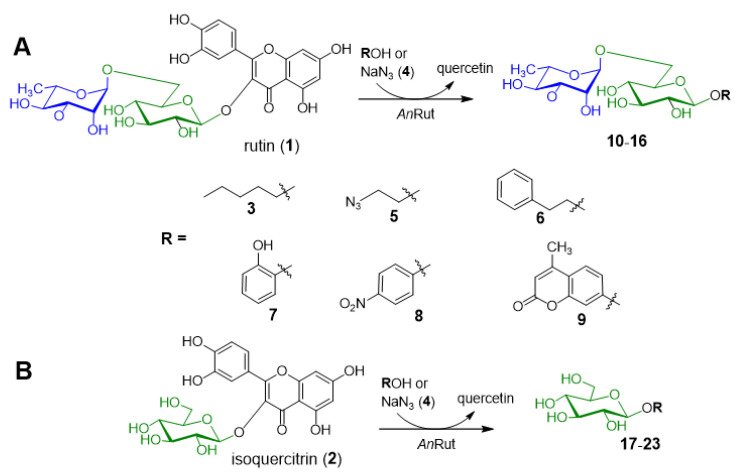
Transglycosylation reactions catalyzed by recombinant *An*Rut. Reactions with rutin (**A**) and isoquercitrin (**B**) as glycosyl donors with various acceptors.

The basic transglycosylation properties of *An*Rut were mentioned in our previous work using rutin as a donor [13]. In the present work, we performed a detailed structure affinity study encompassing several acceptors of varying structural features, namely a primary alcohol, azide-containing compounds, and phenolic compounds. The transglycosylation properties were studied with both *An*Rut activities, i.e., rutinosidase (with rutin donor) and glucosidase (with isoquercitrin donor), yielding a series of novel rutinosides and glucosides (Figure 1).

The transglycosylation reactions were monitored by TLC and they were terminated when most rutin was converted. Rutin and isoquercitrin are hardly soluble in water, which did not allow full conversion of the substrates. Hence, we applied the concept of solid state biocatalysis [17]. Thus, the more soluble glycosylated products could easily be separated from the reaction mixture by filtration. Advantageously, this method allowed us to avoid the use of organic solvents (e.g., dimethyl sulfoxide; DMSO). The structures of the products were confirmed by mass spectrometry (MS) or nuclear magnetic resonance (NMR) spectroscopy, as detailed further.

Here, we describe a rather unprecedented transglycosylation reaction (Table 1). The *An*Rut enzyme was able to efficiently transfer both glucosyl and rutinosyl moieties onto the inorganic ion of N_3_^−^ [26]. This reaction has been previously described with glycosynthase enzymes (mutant glycosidases where the catalytic nucleophile is substituted with a non-nucleophilic residue) in the frame of the so-called enzyme activity rescue [27]. The reaction with wild type *An*Rut and sodium azide acceptor proceeded in a higher yield (44%) with rutin as a donor and less efficiently with isoquercitrin (22%).

### 2.3. Product Stability against Enzymatic Hydrolysis

The common problem of transglycosylations is the reversal cleavage of the product(s) by the enzyme. This complication often strongly diminishes the yields. Therefore, the stability of the transglycosylation products **10**–**23** was tested (Table 2). All the reactions contained the same concentration of transglycosylation product (2 mM) and the same amount of active enzyme (0.8 U/mL). Enzymatic hydrolysis was monitored for 48 h. During this time, none of the products containing an aliphatic aglycone (**10**–**12**, **17**–**19**) was cleaved. Neither reactions with **13** and **20** showed any formation of glucose or rutinose. In contrast, products with a phenolic aglycone (**14**–**15**, **21**–**22**) were hydrolyzed by *An*Rut and the sugar moiety was detected by TLC. The low resistance of **15** and **22** against enzymatic hydrolysis was expected since **15** is commonly used for rutinosidase activity determination [13,17]. These products exhibited complete hydrolysis during the first 4 h of the reaction.

### 2.4. Application of Transglycosylation Products

Azide containing oligosaccharides can easily be attached to multivalent scaffolds, such as functionalized pyrogallol (*tri*-*O*-propargyl-1,2,3-benzentriol (**24**) via Cu^+^-catalyzed azide-alkyne cycloaddition reaction (“click chemistry”) to create rhamnosyl-decorated glycoconjugates. These multivalent rhamnose carrying structures can be used in search for rhamnose-specific lectins and in biological assays for testing inhibitory activities towards AGE-mediated pathologies. The trivalent carrier **24** was synthesized as described previously [28]. Two of the prepared transglycosylation products, rutinosyl azide (**11**) and 2-azidoethyl rutinoside (**12**), were used as possible ligands for multivalent presentation.

**Figure 2:**
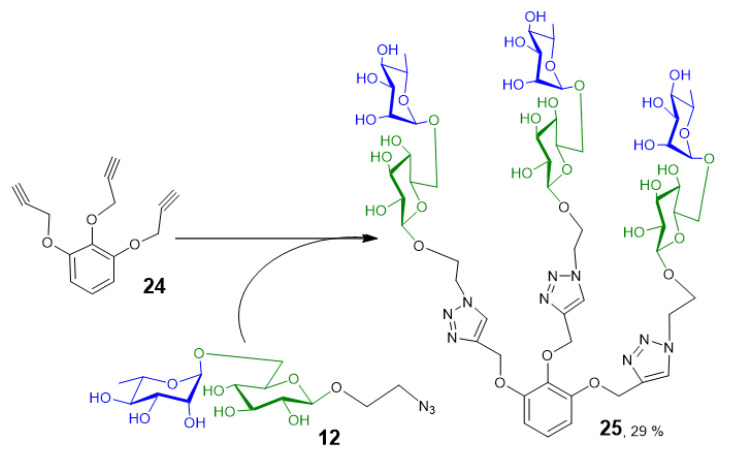
Conjugation of 2-azidoethyl rutinoside (**12**) to the trivalent carrier **23** using Cu^+^-catalyzed azide-alkyne cycloaddition reaction.

Click reactions were performed using the standard protocol comprising sodium ascorbate and CuSO_4_ in methanol/water at 60 °C, for 72 h. Interestingly, the reaction with rutinosyl azide (**11**) did not show any notable conversion, probably due to the steric hindrance caused by the lack of a spacer between the carrier and the rutinosyl moiety. In contrast, the reaction with 2-azidoethyl rutinoside (**12**) yielded the multivalent glycoconjugate **25** (Figure 2). Compound **25** was isolated in a final yield of 29%.

### 2.5. AnRut Structure and Description of Substrate Tunnels

**Figure 3:**
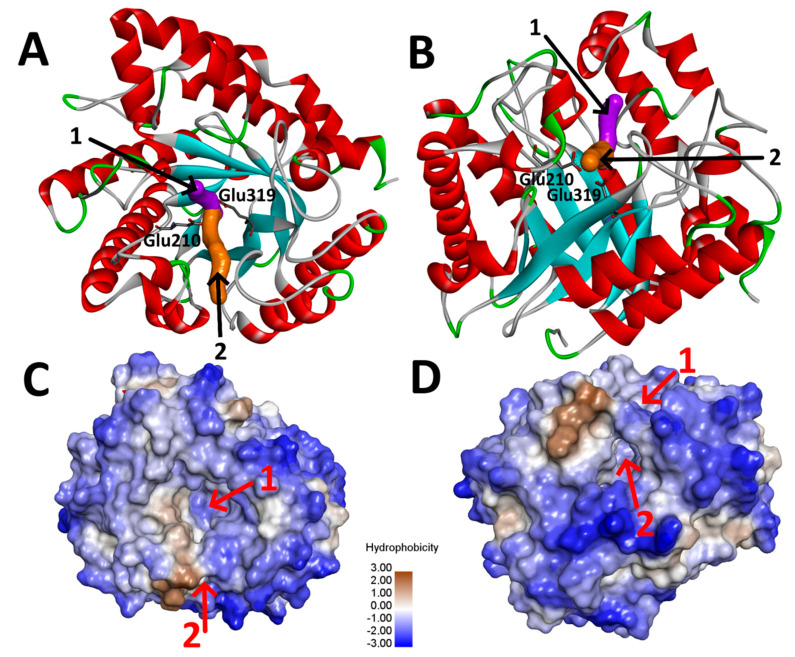
Tunnels leading to the active site of *AnRut* protein (PDB ID: 6I1A). (**A**,**B**) Main (magenta) and side (orange) tunnels within the ribbon model, the active site residues Glu319 and Glu210 are shown as stick models; (**C**,**D**) The views of the main (1) and side tunnels (2) within the hydrophobic surface model.

The term tunnel in biochemistry generally refers to a pathway connecting a protein surface with an internal cavity or a pathway connecting several internal cavities [29]. Accessibility of individual pathways for different substances is largely governed by their size, shape, and amino acid composition and can be efficiently modified by protein engineering [30,31,32,33]. A deep pocket of *An*Rut protein structure is located at the center of the (β/α) barrel architecture. The pocket forming the active site of the enzyme is comprised of residues in loop regions that connect the C-terminal ends of the β-sheets to the following α-helices [18]. In the crystal structure of *An*Rut, our CAVER calculations revealed the presence and parameters of two tunnels leading to the active site (Figure 3). The main wider tunnel has an average tunnel length of 8.8 Å, its average bottleneck radius is 2.2 Å, and its average tunnel throughput is 0.90 (this parameter describes the probability that the pathway is used as a route for transport of the substances, maximal value is 1.0). The second tunnel has an average length of 15.2 Å, an average bottleneck radius of 1.4 Å, and an average tunnel throughput of 0.67 [34]. The output of CAVER 3.0 provided us with the necessary data for CAVERDOCK computational analysis of the time evolution of individual pathways.

**Figure 4:**
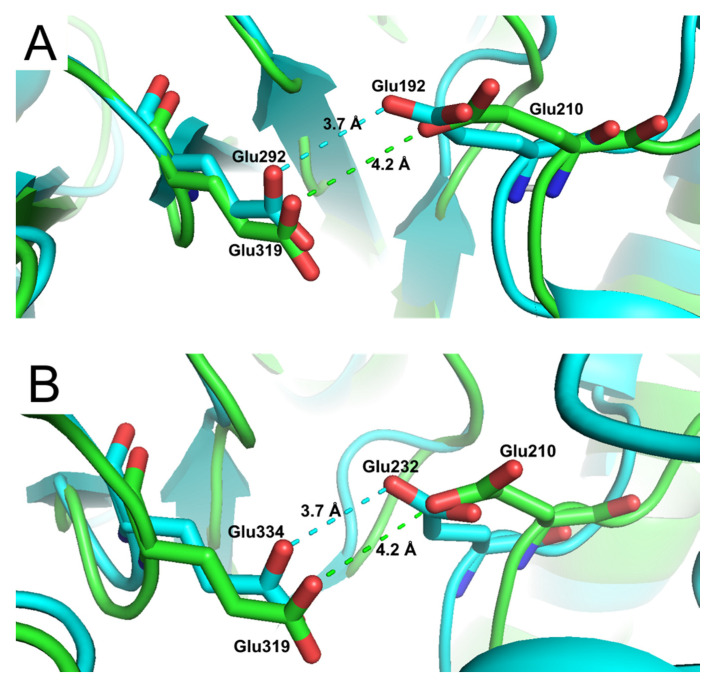
Overlay of the *AnRut* catalytic active site (green) with (**A**) *exo*-β-(1,3)-glucanase (blue) from *Candida albicans* (*Ca*Exg) or with (**B**) *exo*-β-(1,3)-glucanase (blue) from *Saccharomyces cerevisiae* (*Sc*Exg). Distances between active site residues Glu210 and Glu319 of *AnRut* (3.7 Å) and the corresponding residues (**A**) Glu192 and Glu292 (4.2 Å) in *Ca*Exg and (**B**) Glu232 and Glu334 (4.2 Å) in *Sc*Exg are displayed. Despite the low sequence identity of ca 20%, a high structural similarity between *An*Rut and both *exo*-β-(1,3)-glucanases was found in their catalytic sites.

Despite a low sequence identity of ca 20%, we found a high structural similarity between the catalytic sites of *An*Rut and of two *exo*-β-(1,3)-glucanases (Exgs) from *Candida albicans* (*Ca*Exg, PDB code: 1CZ1 [24]) and from *Saccharomyces cerevisiae* (*Sc*Exg, PDB code: 1H4P [25]) (Figure 4), including their –1 subsites, which in both cases bind the glucosyl moieties of their respective substrates [18].

**Figure 5:**
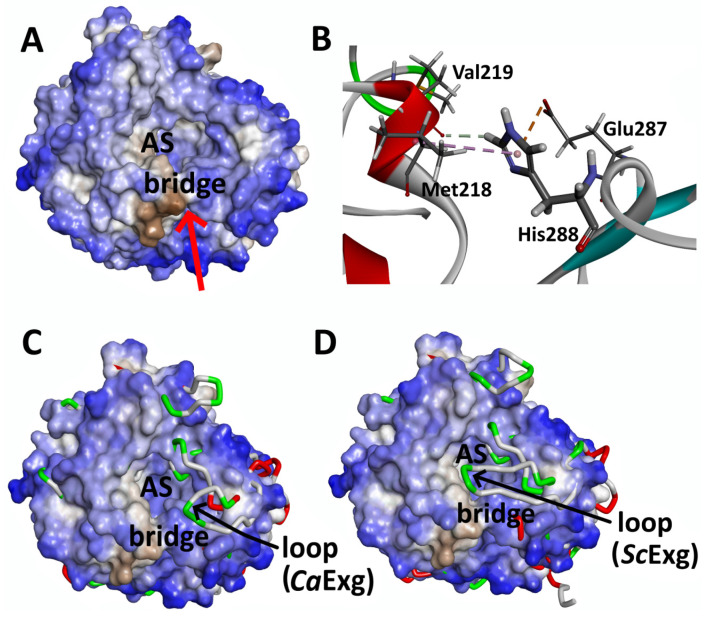
The structural similarity of *An*Rut, *Ca*Exg, and *Sc*Exg in the area of deep valley leading to the protein active site (AS). (**A**) Surface model of *An*Rut protein colored by hydrophobicity with a deep bridged valley indicated by a red arrow; (**B**) View of the bridge residues of *An*Rut, which span the valley via non-covalent interactions caused by the presence of His288 (His288-Val219 via π-alkyl, His288-Met218 via van der Waals, and His288-Glu287 via π-anion interactions); (**C**) Hydrophobic surface model *An*Rut superimposed into *Ca*Exg tube model revealing the existence of a flexible loop in *Ca*Exg (Glu314-Ile323) over the valley; (**D**) Hydrophobic surface model of *An*Rut superimposed into *Sc*Exg tube model revealing the existence of a large loop in *Sc*Exg (Gly357-Cys372) over the valley.

The comparison of the *An*Rut surface area (colored according to hydrophobicity) with structurally similar enzymes *Ca*Exg and *Sc*Exg revealed that all three enzymes contained a weakly hydrophilic deep valley pathway leading to the active site pocket with significant hydrophobic areas in *An*Rut (Figure 5).

In the case of *An*Rut, the valley is bridged (Figure 5A,B) by non-covalently bound residues His288, Val219, Met218, and Glu287 forming a second tunnel (detected by CAVER), which has not been observed so far in any other glycosidase. For comparison, the main entrance to the active site pocket of *Sc*Exg was partially reduced by a large flexible loop Gly357-Cys372 (Figure 5C), which could potentially play the role of a bridge analogous to the *An*Rut structure. A similar phenomenon could be observed in *Ca*Exg, in which the valley was partially covered by a loop Glu314-Ile323, six residues shorter than in *Sc*Exg (Figure 5D). Contrary to *An*Rut, the loops in *Ca*Exg and *Sc*Exg leave the valley partially open, and thus, no side tunnel existed (see Supplementary Figure S3). The superposition of all three protein structures was also performed and visualized to get a deeper insight into their structural similarity and differences (see Supplementary Figure S4).

To evaluate the stability of the second tunnel in the *An*Rut structure, molecular dynamics simulations in GROMACS were performed. The 100 ns molecular dynamics simulations and the following root mean square fluctuation (RMSF) analysis to reproduce the flexibility of protein residues (analogous to crystallographic B-factors) revealed the most flexible protein area within the second tunnel part (loop Asn215-Pro224); nevertheless, during the whole simulation time the tunnel remained preserved (Supplementary Figure S5). The extreme flexibility of this loop, and the ability to approach the amino-acid triad His-Glu-Tyr could be the crucial parameters for the tunnel formation and stability.

### 2.6. Dual Substrate Specificity and Substrate Docking

Initially, we attempted to determine the crystal structure of *An*Rut in complex with the substrate rutin or the product rutinose. After our extensive but unsuccessful efforts to co-crystallize the *An*Rut complex with rutin or rutinose [18], we decided to model rutin in the active site of *An*Rut using the available structure of *Ca*Exg complexed with the substrate laminaritriose as a starting point (PDB ID: 3N9K [35]). In this work we modeled the *An*Rut complex with rutin and with isoquercitrin using CAVER and CAVERDOCK as powerful molecular docking tools.

The *An*Rut-rutin complex (Supplementary Figure S6) was obtained by transportation of the substrate from the outside environment into the receptor active site. The most favorable binding mode (energy score −8.3 kcal/mol) of rutin was located near the catalytic acid/base Glu195 (with oxygen atom of carbonyl nonparticipating in peptide bond-Oε1) with the shortest distance 3.2 Å from the glycosidic bond oxygen. We identified polar and hydrogen bonds, π-alkyl, π-lone pair, and π–π T-shaped interactions in the modeled complex. It is obvious that all kinds of π interactions are present only between the aglycone and the *An*Rut sidechains. The aglycone is clamped by Leu162 and two aromatic sidechains of Phe221 and Tyr286, building π interactions with two ring structures of the bound quercetin moiety. Additionally, C-7 hydroxyl of the aglycone (A ring of quercetin) forms H-bond with His288. The His288 and Tyr286 residues significantly participate in the formation of the side tunnel. The glucose subunit forms three strong H-bond interactions with Glu210, Glu315, and Tyr286 residues. The rhamnose unit of rutin is the least important for substrate binding as it forms only one H-bond with Glu35 residue. Thus, based on the analysis of interactions in the modeled complex, the aglycone and the glucose moiety appear to substantially contribute to the binding of rutin to the *An*Rut active site through an extensive number of non-covalent interactions (Supplementary Figure S6).

In the *An*Rut-isoquercitrin complex (Supplementary Figure S7A,B), two different most favorable binding modes of isoquercitrin (energy scores −8.4 and −8.0 kcal/mol, respectively) were found near the catalytic acid/base residue Glu210 (oxygen-ε1) with the shortest distance of 3.2 Å from the glycosidic oxygen. The first binding mode with an energy score of −8.4 kcal/mol (Supplementary Figure S7A) is deeply inserted in the side tunnel with the catechol moiety (ring B) of quercetin. In addition to van der Waals interactions, we identified other non-covalent interactions, primarily hydrogen bonds and π interactions in the modeled complex. The π interactions with Phe221, Tyr286, Glu50, and Leu162 residues are present only between the aglycone and the *An*Rut sidechains, whereas the glucose unit forms five significant H-bonds with Glu210, Glu319, Tyr284, and Tyr286 residues. Both the aglycone and the glucosyl moiety appear to contribute to the binding of substrate to the *An*Rut active site.

In contrast to the first (energetically most favorable) binding mode of isoquercitrin, the second binding mode (Supplementary Figure S7B) is deeply inserted in the side tunnel with the main part of aglycone (rings A and C) similar to the rutin binding mode (Supplementary Figure S6). In addition to the many van der Waals interactions, non-covalent π interactions were present only between the aglycone and the *An*Rut sidechain residues Phe221, Tyr286, and Leu162, whereas two H-bonds were found between the aglycone and His288 and Glu50 residues, and three H-bonds between the glucose unit and Glu210, Glu319, and Tyr284 residues. On the basis of the analysis of interactions in the modeled complex, both the aglycone and the glucose moiety appear to contribute to the binding of rutin in the *An*Rut active site through an extensive number of non-covalent interactions. It seems that α-rhamnosyl moiety is not quite crucial for the binding, therefore, we also consider *An*Rut to be a “β-glucosidase with a broad substrate specificity”.

### 2.7. Acceptor Specificity of AnRut

**Figure 6:**
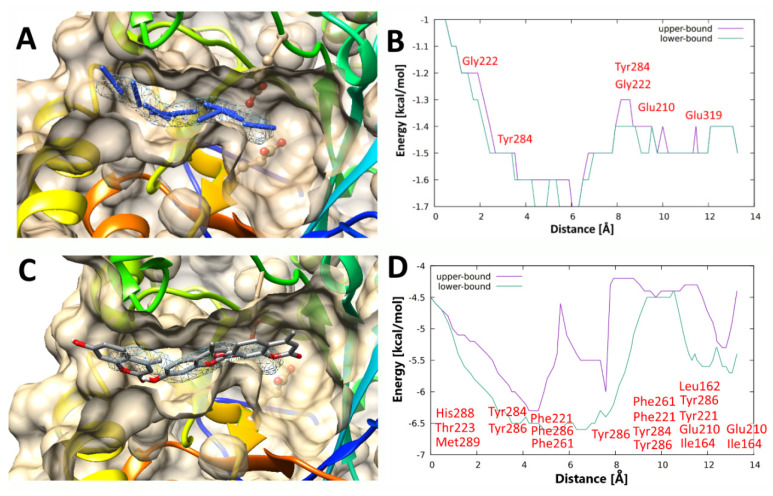
Selected snapshots of (**A**) the best (azide, **4**) and (**C**) the worst (4-methylumbelliferone, **9**) acceptors passing through the second tunnel of *An*Rut. At a specific point close to the entry to the tunnel, most acceptors form a temporary hydrogen bond with the peptide carbonyl of Gly222 residue (2.2 Å distance). Thus, Gly222 could be the major driving force attracting the acceptor molecule and dragging it into the tunnel. The respective energy profiles of acceptor passages are shown in panels (**B**) (for **4**) and (**D**) (for **9**). The lower-bound trajectory (green) is not contiguous; however, it samples the tunnel without any gap in the substrate movement and some bottlenecks may not be detected. The upper-bound trajectory (magenta) is contiguous, it is the best trajectory found; however, a trajectory with better energy profile may exist, the actual energy would then be the same or lower. During the acceptor (azide, **4** or 4-methylumbelliferone, **9**) passage through the side tunnel, non-covalent interactions of amino acid residues (indicated in red in panels (**B**,**D**)) with respective acceptors were monitored.

We hypothesize that the special shape, size, and specific interactions of the *An*Rut second tunnel leading to the active site play a significant role in transglycosylation reactions with rutin or isoquercitrin donors, respectively, and a broad range of acceptors including phenolic compounds (Figure 6). It appears that the variable substrate specificity of acceptors could be related to the acceptor accessibility through the tunnel during their way to the *An*Rut active site. To verify this hypothesis, we calculated pathways through the side tunnel with the CAVERDOCK technique for all acceptors used in our in vitro experiments.

In silico experiments (trajectories of acceptor passages) were visualized, and binding energy profiles were analyzed and compared to our experimental yields of transglycosylation products obtained from seven structurally diverse glycosyl acceptors. The best transglycosylation acceptor, azide anion (**4**) (experimental yield of transglycosylation 44%), passed through the tunnel smoothly (Figure 6A) and a low energy barrier (ca 0.4 kcal/mol, Figure 6B) was detected on its way to the active site. The azide anion has recently been identified as a glycosyl acceptor with wild type *An*Rut forming β-rutinosyl azide with retention of anomeric configuration [26]. However, 4-methylumbelliferone (**9**) passed through the tunnel (Figure 6C) with a much higher energy barrier (ca. 2 kcal/mol, Figure 6D) and in an inappropriate position (opposite direction) of the hydroxyl group for the attack of the glycoside donor. Therefore, the 4-methylumbelliferone was evaluated as the worst acceptor, which corresponds to our in vitro experiments (very low yield, due to its paucity the product could not be isolated in sufficient purity and amount for NMR analysis).

**Figure 7:**
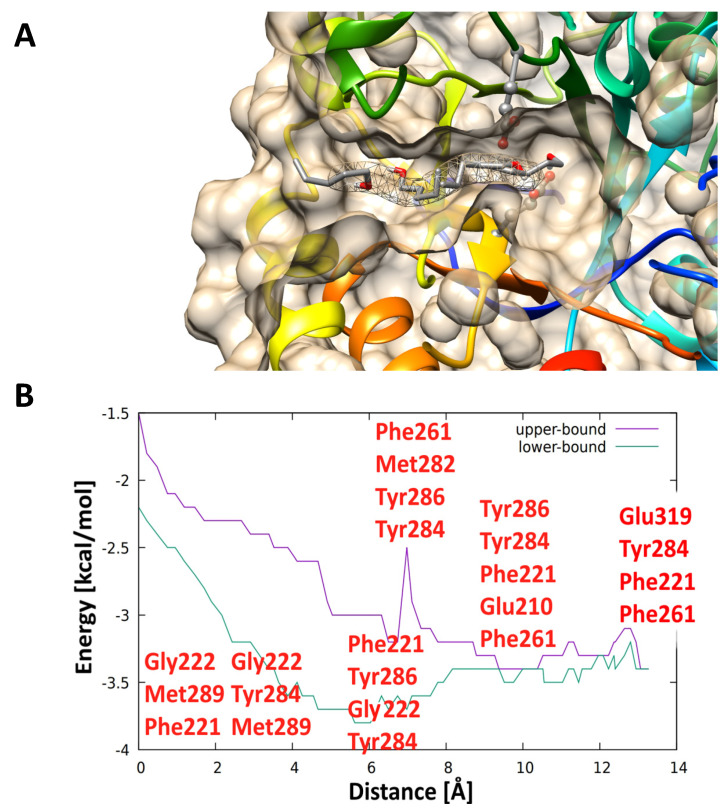
(**A**) Selected snapshots of aliphatic acceptor (pentanol, **3**), passing through the second tunnel to the active site of *An*Rut protein; (**B**) binding energy profile of acceptor **3** passing through the side tunnel. During the acceptor passage through the side tunnel, the non-covalent interactions of amino acid residues (indicated in red in panel (**B**)) with acceptor **3** were recorded.

A typical aliphatic alcohol acceptor, i.e., pentanol was also one of the best acceptors, which provided a good yield of transglycosylation product with both rutin and isoquercitrin according to the in vitro experiments, and in coherence with in silico results (Figure 7). During the passage through the side tunnel, pentanol was initially attracted by Met289 forming alkyl interactions (2.0 Å), then, it was strongly attracted by carbonyl of Gly222 forming a 2.3 Å long H-bond. The aliphatic moiety of pentanol was further attracted to Tyr284, Tyr286, Phe221, and Phe261 via π-alkyl interactions, to His282 via H-bond of carbonyl part, and to Glu210 via H-bond of carboxylate. These interactions forced pentanol to move forward to the active site residue Glu319, creating a H-bond (2.3 Å). For a video showing pentanol passage through the side tunnel see Supplementary Video S1.

## 3. Discussion

The pioneer transglycosylation reactions with *An*Rut have been described by Šimčíková et al. [13] who showed that *An*Rut can synthesize a variety of transglycosylation products. Those reactions were performed with purified enzyme and a fully dissolved substrate (rutin in DMSO). DMSO is rather detrimental for the enzyme activity. In the present study, we synthesized several transglycosylation products using crude recombinant enzyme, which is a convenient procedure, more suitable for a scale-up. Additionally, the “solid state catalysis” concept was employed using the substrates in a suspension for the reaction to occur, as previously demonstrated with this enzyme [17]. The present results showed that *An*Rut could also catalyze transglycosylation reactions under the conditions of “solid state catalysis”, which was previously reported only for hydrolytic reactions. Thus, *An*Rut appears to be a robust enzyme without the need of any special treatment and requirements, which makes it an attractive tool in biotechnology and biomedicine.

In our work, we synthesized fourteen transglycosylation products by rutinosylation or glucosylation of a series of acceptors. Yields of typical preparative transglycosylation reactions are shown in Table 1. From these data, we can roughly estimate enzyme affinity to respective acceptors, although this estimate can be influenced, for example, by the feasibility of product isolation. We conclude that *An*Rut has a high affinity to azide anion and to primary alcohol acceptors. In contrast, phenolic compounds seem to be less efficient acceptors for glycosylation, due to a more difficult passage through the side tunnel. Moreover, glucosides and rutinosides of phenolic acceptors **7**–**9** can be hydrolyzed by the enzyme [13,15,36], which makes the isolation of these products challenging and lowers the glycosylation yield. 2-Phenyl ethanol (**6**) as an acceptor seems to be on the verge between both groups, as reflected in its mediocre transglycosylation yield.

The major aim of this study was to decipher the relation between the acceptor structure and the transglycosylation potential of *An*Rut. We created the molecular model of *An*Rut enzyme based on the recently published crystal structure [18] and modeled the enzyme interactions with the respective substrates and acceptors in silico. We found the active site to be a deep cave connected to the enzyme surface with a side tunnel. Similar tunnels were found in enzymes of glycosidase families GH6 [35] and GH7 [25]. *An*Rut seems to be the first glycosidase within the GH5 family where a tunnel structure was described.

By docking **1** and **2** to the active site, we confirmed that the substrate was bound to the enzyme mainly by its aglycone. Other interactions existed between the enzyme and the glucose subunit, however, the rhamnosyl moiety in **1** exhibited no significant interactions with the enzyme. This fact insinuates that *An*Rut is a β-glucosidase with a broad substrate specificity including a diglycosidase activity [13]. Molecular modeling also explained why some transglycosylation products (**10**–**13**, **17**–**20**) cannot be cleaved by the enzyme (Table 2). Compounds with a small aliphatic aglycone (**10**–**12**, **17**–**19**) are unable to form the necessary interactions with the amino acid sidechains in the enzyme active site to hold the substrate in the correct position for catalysis. In contrast, the transglygosylation products with aromatic aglycones (**8**–**9**, **22**–**23**) are well fixed in the active site mainly by interacting with the binding site similar to the quercetin moiety and act as substrates of the enzyme, which is probably the main reason for the relatively low isolated yields in transglycosylations with phenolic acceptors (Table 1). This does not seem to be the case for the phenethyl glycosides (**13** and **20**) where the aromatic moieties are separated from their glycosides by a C_2_ spacer, leading to a conformation that does not allow proper binding to the active site, resulting in their resistance to enzymatic hydrolysis.

We assumed that the structure of the side tunnel close to the active site in the *An*Rut structure could also affect the progress of transglycosylation reactions. The tunnel leads to the catalytic amino acids (Glu210 and Glu319) and it appears that it is the pathway, by which acceptors approach the active site to participate in the transglycosylation process. We traced the path for each acceptor and found that their passage through the side tunnel was strongly dependent on the acceptor size, shape, and hydrophobicity.

The results of in silico experiments agree well with the results of transglycosylation reactions, and thus support our hypothesis on the significant role of the side tunnel in the synthetic activity of *An*Rut. The validation and analysis of tunnel importance for the enzymatic activity with selected acceptors will be the subject of our future studies focused on point mutations in the side tunnel and in the transglycosylation acceptor binding site.

Another highlight of our work is the high yield of transglycosylation reactions with inorganic azide (product 22–44% yield). Glycosylation of inorganic azide by a wild type enzyme is a new phenomenon, which has recently been shown in several rutinosidases from different sources [26]. Inorganic azide is commonly used in what is called “activity rescue” reaction in glycosidases, when the catalytic site of the enzyme is mutated and needs to receive a nucleophile from the outside for the catalytic reaction to occur. Wild type enzymes do not need an external nucleophile, but the inorganic azide is an efficient acceptor of both rutinosyl and glucosyl moieties in the catalytic reaction with wild type *An*Rut. The higher yield for rutinosyl azide (**11**) as compared with glucosyl azide (**18**) is explained by the differences between the enzyme activity towards β-glucosides and rutinosides [18].

Transglycosylation products containing an azido moiety (**11**–**12**, **18**–**19**) can be used for creating multivalent structures, for example, by Cu^+^ catalyzed azide-alkyne cycloaddition (“click reaction”). Interestingly, in the case of azido-functionalized rutinosylated products **11** and **12**, the click reaction acted significantly better if rutinose was presented on a longer linker (**12**) as compared with azide being directly attached (**11**). Probably the short distance between the disaccharide and the azide residue caused steric hindrance that did not allow **11** to properly bind to the carrier **24**. The click reaction with azide directly attached to the carbohydrate moiety would probably need longer optimization, such as the employment of microwaves. The prepared trivalent glycoconjugate (**25**) which was synthesized from transglycosylation product **12**, will be tested in further biological experiments as a carrier of rhamnosyl moiety to prevent fibrosis and aging [20,21]. It shows a pathway to possible biotechnological application of *An*Rut for chemoenzymatic synthesis of rhamnosyl-capped glycoconjugates.

## 4. Materials and Methods

### 4.1. Materials

Rutin was obtained from Alchimica (Prague, Czech Republic), isoquercitrin was prepared using α-l-rhamnosidase from *A. terreus* from rutin, as described previously [37]. Acceptors 2-azidoethanol and sodium 4-methylumbelliferone were from Sigma-Aldrich (Prague, Czech Republic), 2-phenylethanol was from Honeywell Fluka (Bucharest, Romania), sodium azide was provided by Alfa Aesar (Kandel, Germany), catechol was from Acros Organics (Geel, Belgium), pentan-1-ol was from VWR International (Stříbrná Skalice, Czech Republic), and *p*-nitrophenol was from Lachema (Brno, Czech Republic). Pyrogallol (1,2,3-benzentriol) was provided by SERVA (Heidelberg, Germany). Kits for Molecular Weights for Gel Filtration were obtained from Sigma-Aldrich (Prague, Czech Republic) and the EasySelect Pichia Expression Kit was from Invitrogen (Carlsbad, CA, USA).

### 4.2. Recombinant Rutinosidase from A. niger

Competent *Pichia pastoris* KM71H cells were transformed with the expression vector pPICZαA-RUT, obtained as described previously [13]. The nucleotide sequence of this enzyme is deposited in GenBank under the accession number MN393234. Heterologous expression was done according to the manufacturer’s instructions (EasySelect Pichia Expression Kit, Invitrogen, Carlsbad, CA, USA) and the recombinant protein was produced as a fusion product into the growth medium. For the experiments, we used crude cultivation medium, as described previously [17], void of other enzyme activities. The enzyme was used in transglycosylation reactions unpurified, dissolved in the cultivation medium.

Protein concentrations were determined by a Bradford assay calibrated for bovine plasma γ-globulin (IgG, BioRad, Watford, Hertfordshire, UK). The quaternary structure of the enzyme (monomer or dimer) was determined by size exclusion chromatography. The enzyme sample (7.6 mg protein) was loaded on Superdex™ 200 10/300 column (30 cm × 10 mm; Sigma-Aldrich, Prague, Czech Republic) with 50 mM citrate-phosphate/150 mM NaCl buffer pH 5.0 as a mobile phase at an elution rate of 0.4 mL/min. The elution volume of the sample was compared with the following calibration markers (Kits for Molecular Weights, Sigma-Aldrich, St. Louis, MO, USA): cytochrome c, carbonic anhydrase, bovine serum albumin, alcohol dehydrogenase, apoferritin, and blue dextran.

### 4.3. Enzyme Activity Assay

Rutinosidase activity was measured spectrophotometrically using *p*-nitrophenyl rutinoside, which was prepared as previously described [13,17]. The reaction mixture contained substrate (10 mM solution, 10 µL), 50 mM McIlvaine buffer pH 3.5, and crude *An*Rut (total volume 50 µL). The reaction was incubated at 36 °C, for 10 min, with shaking at 550 rpm, and stopped by adding 1 mL of 0.1 M Na_2_CO_3_ to the reaction mixture. The released *p*-nitrophenol was determined spectrophotometrically at 420 nm. One unit of enzymatic activity was defined as the amount of enzyme releasing 1 µmol of *p*-nitrophenol per minute under the assay conditions.

### 4.4. Analytical Transglycosylation Reactions

Transglycosylation activity of *An*Rut with acceptors **3**–**9** was assessed in a total volume of 550 µL (0.1–2 M isoquercitrin or rutin; 0.6–3 M acceptor and 500 µL of crude *An*Rut, 0.8 U/mL; pH of the reaction mixture was adjusted to 3.5 with H_3_PO_4_ [13]). Reactions ran at 36 °C with shaking (550 rpm). The formation of the transglycosylation product (β-d-glucoside or rutinoside) was monitored by TLC (ethyl acetate/2-propanol/H_2_O = 3:2:2, *v*/*v*/*v*). The presence of the desired transglycosylation product in the analytical transglycosylation reactions was confirmed by HPLC-MS (high performace liquid chromatography combined with mass spectrometry).

### 4.5. Preparative Transglycosylation Reactions

General biosynthetic procedure (compounds **10**–**12**, **14**–**19**, **21**–**23**): Rutin (for products **10**–**16**) or isoquercitrin (for products **17**–**23**) were mixed with the respective acceptor (**3**–**9**), in a molar ratio of 3–6-fold excess over the acceptor. The crude *An*Rut (0.8 U/mL) was added and pH was adjusted to pH 3.5 (H_3_PO_4_). Reactions ran at 36 °C with shaking (550 rpm). All reactions were monitored by TLC (mobile phase: ethyl acetate/2-propanol/H_2_O = 3:2:2, *v*/*v*/*v*) and stopped by heating (95 °C, 10 min). The undissolved rutin or isoquercitrin and produced quercetin were removed by centrifugation (4500× *g*, 15 min at 15 °C) and subsequent filtration. The supernatants were evaporated in vacuo to dryness, dissolved in mobile phase (dichloromethane/methanol = 4:1, *v*/*v*) and purified by silica gel chromatography (15–20 × 500 mm). Fractions containing the product were combined and evaporated in vacuo to dryness. Then, products **10**–**14**, **18**–**19** were dissolved in *tert*-butyl alcohol and lyophilized.

Compounds **13** and **20** were synthesized as described for the general biosynthetic procedure and isolated using a BioGel P-2 column (26 × 1000 mm, Bio-Rad, Prague, Czech Republic).

Pentyl rutinoside (**10**, pentyl α-l-rhamnopyranosyl-(1-6)-β-d-glucopyranoside): The reaction mixture contained rutin donor (6 g, 9.8 mmol), pentanol acceptor (6 mL, 55.5 mmol), and 26 mL of crude *An*Rut (21 U). The reaction proceeded according to the general biosynthetic procedure. The product was isolated as a white amorphous solid; yield of 620 mg (16% relative to the donor). Compound purity was inferred from ^1^H NMR spectrum to be higher than 98%. For ^1^H and ^13^C NMR see Supplementary Table S1 and Figure S8A,B; MS (ESI^+^): [M + Na]^+^, *m/z* 419.2; HRMS (ESI^+^): calcd for C_17_H_32_O_10_Na^+^: 419.18877, found 419.18890 (0.3 ppm), see Supplementary Figure S8C.

Rutinosyl azide (**11**, α-l-rhamnopyranosyl-(1-6)-β-d-glucopyranosyl azide): The reaction mixture contained rutin donor (2 g, 3.28 mmol), sodium azide acceptor (4.3 g, 66.1 mmol), and 16 mL of crude *An*Rut (13 U). The reaction proceeded according to the general biosynthetic procedure. The product was isolated as a white amorphous solid; yield of 530 mg (44% relative to the donor). Compound purity was inferred from ^1^H NMR spectrum to be 89%. For ^1^H and ^13^C NMR see Supplementary Table S2 and Figure S9A,B; MS (ESI^+^): [M + Na]^+^, *m/z* 374.1; HRMS (ESI^+^): calcd for C_12_H_21_O_9_N_3_Na^+^: 374.11700, found 374.11709 (0.2 ppm), see Supplementary Figure S9C.

2-Azidoethyl rutinoside (**12**, 2-azidoethyl α-l-rhamnopyranosyl-(1-6)-β-d-glucopyranoside): The reaction mixture contained rutin donor (3 g, 5 mmol), 2-azidoethanol acceptor (1.7 mL, 22.4 mmol), and crude *An*Rut (22 mL, 16 U). The reaction proceeded according to the general biosynthetic procedure. Product was isolated as a white amorphous solid; yield of 310 mg (16% relative to the donor). Compound purity was inferred from ^1^H NMR spectrum to be higher than 97%. For ^1^H and ^13^C NMR see Supplementary Table S3 and Figure S10A,B; MS (ESI^+^): [M + Na]^+^, *m/z* 418.1; HRMS (ESI^+^): calcd for C_14_H_25_O_10_N_3_Na^+^: 418.14322, found 418.14327 (0.1 ppm), see Supplementary Figure S10C.

2-Phenylethyl rutinoside (**13**, 2-phenylethyl α-l-rhamnopyranosyl-(1-6)-β-d-glucopyranoside)**:** The reaction mixture contained rutin donor (0.61 g, 1 mmol), 2-phenylethanol acceptor (720 µL, 6.01 mmol), and crude *An*Rut (10 mL, 8 U). The reaction proceeded according to the general biosynthetic procedure, with purification on BioGel P-2 column. The product was isolated as a light yellow amorphous solid; yield of 38 mg (8.8% relative to the donor). Compound purity was inferred from ^1^H NMR spectrum to be higher than 97%. For ^1^H and ^13^C NMR see Supplementary Table S4 and Figure S11A,B; MS (ESI^+^): [M + Na]^+^, *m/z* 453.2; HRMS (ESI^+^): calcd for C_20_H_30_O_10_Na^+^: 453.17312, found 453.17305 (−0.1 ppm), see Supplementary Figure S11C.

Catechol rutinoside (**14**, 2-hydroxyphenyl α-l-rhamnopyranosyl-(1-6)-β-d-glucopyranoside): The reaction mixture contained rutin donor (0.5 g, 0.82 mmol), catechol acceptor (0.9 g, 8.2 mmol), and 5 mL of crude *An*Rut (4 U). The reaction proceeded according to the general biosynthetic procedure. The product was isolated as a light orange amorphous solid; yield of 10.1 mg (7.4% relative to the donor). ^1^H and ^13^C NMR data were in accordance with the structure, as published previously [13]. MS (ESI^+^): [M + Na]^+^, *m/z* 441.1; HRMS (ESI^+^): calcd for C_18_H_26_O_11_Na^+^: 441.13673, found 441.13643 (−0.7 ppm), see the Supplementary Figure S12.

*p*-Nitrophenyl rutinoside (**15**, 4-nitrophenyl α-l-rhamnopyranosyl-(1-6)-β-d-glucopyranoside): The reaction mixture contained rutin donor (0.263 g, 0.43 mmol), *p*-nitrophenol acceptor (0.18 g, 1.3 mmol), and crude *An*Rut (4 U, 5 mL). The reaction proceeded according to the general biosynthetic procedure. The product was isolated as a yellow amorphous solid; yield of 12.5 mg (6.5% relative to the donor). Compound purity was inferred from ^1^H NMR spectrum to be higher than 90 %. For ^1^H and ^13^C NMR see Supplementary Table S5 and Figure S13A,B; MS (ESI^+^): [M + Na]^+^, *m/z* 470.1; HRMS (ESI^+^): calcd for C_18_H_25_O_12_Na^+^: 470.12690, found 470.12672 (−0.4 ppm), see Supplementary Figure S13C.

4-Methylumbelliferyl rutinoside (**16**, 4-methylumbelliferyl α-l-rhamnopyranosyl-(1-6)-β-d-glucopyranoside): The reaction mixture contained rutin donor (0.263 g, 0.4 mmol), sodium 4-methylumbelliferone acceptor (0.256 g, 1.3 mmol), and 5 mL of crude *An*Rut (0.8 U). The reaction proceeded according to the general biosynthetic procedure. The product was purified as a yellowish amorphous solid; yield of 11.3 mg (5.4% relative to the donor). The product was not isolated in a sufficient purity for NMR analysis. MS (ESI^+^): [M + H]^+^, *m/z* 485.2; [M + Na]^+^, *m/z* 507.1; HRMS (ESI^+^): calcd for C_22_H_28_O_12_Na^+^: 507.14730, found 507.14716 (−0.3 ppm), see the Supplementary Figure S14.

Pentyl β-d-glucopyranoside (**17**): The reaction mixture contained isoquercitrin donor (0.5 g, 1.08 mmol), pentanol acceptor (540 µL, 5 mmol), and the crude *An*Rut (8 U, 10 mL). The reaction proceeded according to the general biosynthetic procedure. The product was isolated as a white amorphous solid; yield of 42 mg (15% relative to the donor). Compound purity was inferred from ^1^H NMR spectrum to be higher than 98%. For ^1^H and ^13^C NMR see Supplementary Table S6 and Figure S15A,B; MS (ESI^+^): [M + Na]^+^, *m/z* 273.1; HRMS (ESI^+^): calcd for C_11_H_22_O_6_Na^+^: 273.13086, found 273.13100 (0.5 ppm), see Supplementary Figure S15C.

β-d-Glucopyranosyl azide (**18**): The reaction mixture contained isoquercitrin donor (0.5 g, 1.1 mmol), sodium azide acceptor (1.43 g, 22 mmol), and crude *An*Rut (5.6 U, 7 mL). The reaction proceeded according to the general biosynthetic procedure. The product was isolated as a yellowish amorphous solid; yield of 40 mg (22% relative to the donor). Compound purity was inferred from ^1^H NMR spectrum to be higher than 97%. For ^1^H and ^13^C NMR see Supplementary Table S7 and Figure S16A,B; MS (ESI^+^): [M + Na]^+^, *m/z* 228.1; HRMS (ESI^+^): calcd for C_6_H_11_O_5_N_3_Na^+^: 228.05909, found 228.05910 (0.0 ppm), see Supplementary Figure S16C.

2-Azidoethyl β-d-glucopyranoside (**19**): The reaction mixture contained isoquercitrin donor (1 g, 2.15 mmol), 2-azidoethanol acceptor (760 µL, 10 mmol), and crude *An*Rut (8 U, 10 mL). The reaction proceeded according to the general biosynthetic procedure. The product was isolated as a light gray amorphous solid; yield of 85 mg (11% relative to the donor). Compound purity was inferred from ^1^H NMR spectrum to be higher than 90%. For ^1^H and ^13^C NMR see Supplementary Table S8 and Figure S17A,B; MS (ESI^+^): [M + Na]^+^, *m/z* 272.1; HRMS (ESI^+^): calcd for C_8_H_15_O_6_N_3_Na^+^: 272.08531, found 272.08543 (0.4 ppm), see Supplementary Figure S17C.

2-Phenylethyl β-d-glucopyranoside (**20**): The reaction mixture contained isoquercitrin donor (0.5 g, 1.08 mmol), 2-phenylethanol acceptor (720 µL, 6.01 mmol), and 10 mL of crude *An*Rut (8 U). The reaction proceeded according to the general biosynthetic procedure, with purification on BioGel P-2 column. The product was isolated as a white amorphous solid; yield of 23 mg (7% relative to the donor). Compound purity was inferred from ^1^H NMR spectrum to be higher than 98%. For ^1^H and ^13^C NMR see Supplementary Table S9 and Figure S18A,B; MS (ESI^+^): [M + Na]^+^, *m/z* 307.1; HRMS (ESI^+^): calcd for C_14_H_20_O_6_Na^+^: 307.11521, found 307.11530 (0.3 ppm), see Supplementary Figure S18C.

2-Hydroxyphenyl β-d-glucopyranoside (**21**, catechol β-glucoside): The reaction mixture contained isoquercitrin donor (0.2 g, 0.43 mmol), catechol acceptor (0.142 g, 1.29 mmol), and crude *An*Rut (4 U, 5 mL). The reaction proceeded according to the general biosynthetic procedure. The product was isolated as a light orange amorphous solid; yield of 10.7 mg (9.1% relative to the donor). Compound purity was inferred from ^1^H NMR spectrum to be higher than 95%. For ^1^H and ^13^C NMR see Supplementary Table S10 and Figure S19A,B; MS (ESI^−^): [M − H]^−^, *m/z* 271.1; HRMS (ESI^−^): calcd for C_12_H_15_O_7_^−^: 271.08233, found 271.08282 (1.8 ppm), see Supplementary Figure S19C.

*p*-Nitrophenyl β-d-glucopyranoside (**22**): The reaction mixture contained isoquercitrin donor (0.1 g, 0.215 mmol), *p*-nitrophenol acceptor (0.18 g, 1.3 mmol), and 5 mL of crude *An*Rut (4 U). The reaction proceeded according to the general biosynthetic procedure. The product was isolated as a yellow amorphous solid; yield of 5.3 mg (8.2% relative to the donor). Compound purity was inferred from ^1^H NMR spectrum to be higher than 98%. For ^1^H and ^13^C NMR see Supplementary Table S11 and Figure S20A,B; MS (ESI^+^): [M + Na]^+^, *m/z* 324.1; HRMS (ESI^+^): calcd for C_12_H_15_O_8_NNa^+^: 324.06899, found 324.06885 (−0.4 ppm), see Supplementary Figure S20C.

4-Methylumbelliferyl β-d-glucopyranoside (**23**): The reaction mixture contained isoquercitrin (0.2 g, 0.43 mmol) and sodium 4-methylumbelliferone (0.256 g, 1.29 mmol) were mixed in 5 mL of crude *An*Rut (0.8 U/mL). The reaction proceeded according the general biosynthetic procedure. The product was isolated as a light yellow amorphous solid; yield of 7.2 mg (5% relative to the donor). The product was not isolated in sufficient purity for NMR analysis. MS (ESI^+^): [M + H]^+^, *m/z* 339.1; [M + Na]^+^, *m/z* 361.1; HRMS (ESI^−^): calcd for C_16_H_19_O_8_^+^: 339.10744, measured 339.10726 (−0.5 ppm), see the Supplementary Figure S21.

### 4.6. Product Stability against Enzymatic Hydrolysis

Transglycosylation products (2 mM) were mixed with *An*Rut (0.8 U/mL) in a total volume of 200 µL. Reactions were incubated at 36 °C under shaking (550 rpm), for 48 h. After 4, 24, and 48 h, samples were taken from reaction mixtures and analyzed by TLC (dichloromethane/methanol = 4:1, *v*/*v*). Hydrolysis was indicated by glucose or rutinose detection and substrate consumption on the TLC plate. All reactions were accompanied by respective blank reactions void of enzyme.

### 4.7. Structural Characterization

NMR: All nuclear magnetic resonance data were acquired on a Bruker AVANCE III 400 MHz spectrometer except for compounds **12** and **22** acquired on a 600 MHz spectrometer, and except for compound **25** acquired on a 700 MHz instrument (Bruker BioSpin, Rheinstetten, Germany). All NMR experiments were performed at 30 °C in D_2_O; only compounds **10** and **21** were measured in CD_3_OD. Residual signals of solvents (D_2_O, *δ*_H_ 4.732 ppm; CD_3_OD, *δ*_H_ 3.306 ppm and *δ*_C_ 49.05 ppm) were used as internal standards; ^13^C NMR spectra in D_2_O were referenced to the signal of acetone (*δ*_C_ 30.50 ppm) Before Fourier transformation, ^1^H NMR and ^13^C NMR spectra were zero filled to four-fold data points and multiplied by window function. Individual spin systems of monosaccharide units and aglycones were assigned using COSY, 1D-TOCSY, and HSQC experiments. The HMBC experiment enabled us to assign quaternary carbons and join the abovementioned moieties together.

HPLC-MS: Analyses were performed using a Shimadzu Prominence LC analytical system comprising a Shimadzu CBM-20A system controller, Shimadzu LC-20AD binary HPLC pump, a Shimadzu CTO-10AS column oven, a Shimadzu SIL-20ACHT cooling autosampler, and a Shimadzu SPD-20MA diode array detector (Shimadzu, Japan). The sample was dissolved in acetonitrile. The flow rate of mobile phase (acetonitrile) was 0.3 mL/min at 25 °C and the injection volume was 1 μL. The MS-ESI parameters were as follows: positive and negative mode; ESI interface voltage, 4.5 kV and −3.5 kV; detector voltage, 1.15 kV; nebulizing gas flow, 1.5 mL/min; drying gas flow, 15 mL/min; heat block temperature, 200 °C; temperature of desolvation line pipe, 250 °C, SCAN mode 300–600 *m*/*z*; and chromatograms were analyzed using software LabSolutions ver. 5.75 SP2 (Shimadzu, Kyoto, Japan).

HR-MS: High-resolution mass spectra were measured with an LTQ Orbitrap XL hybrid mass spectrometer (Thermo Fisher Scientific, Waltham, MA, USA) equipped with an electrospray ion source. The samples were dissolved in methanol and injected using a 2 μL loop into the mobile phase consisting of methanol/water (4:1 *v*/*v*, flow rate 100 μL/min). The spectra were recorded in Orbitrap operated with a resolution of 100,000 and processed using Xcalibur software (Thermo Fisher Scientific).

### 4.8. Synthesis of 1,2,3-tris-[1-(α-l-rhamnopyranosyl-(1→6)-β-d-glucopyranosyl)-1H-1,2,3-triazol-4-yl)-2-ethyloxy]benzene (25)

The reaction mixture contained 1 equivalent of the carrier *tris*-*O*-propargyl-1,2,3-benzentriol (**24**; 10 mg, 41.6 µmol) [26], 3.1 equivalents of the respective transglycosylation product (**11**: 45.2 mg, 130 µmol or **12**: 51 mg, 129 µmol), and sodium ascorbate (8.24 mg, 41.6 µmol) in a total volume of 1.035 mL of H_2_O/methanol mixture in a ratio of 2/5. The reaction mixture was bubbled with argon, and then 5.2 mg of copper sulfate (in 60 µL H_2_O) was added. The reaction ran under argon atmosphere at 60 °C with stirring. After 72 h, maximum conversion was detected under TLC (propan-2-ol/H_2_O/NH_4_OH aq. = 7:2:1, *v*/*v*/*v*). Methanol was removed by evaporated in vacuo and the reaction mixture was loaded on a BioGel P-2 column (26 × 1000 mm, Bio-Rad) in water as a mobile phase, at 6.8 mL/h. The fractions containing the title product were combined and lyophilized. Compound **25** was acquired as white solid in a yield of 17.3 mg (29% related to the carrier **24**). Compound purity was inferred from ^1^H NMR spectrum to be higher than 96%. For ^1^H and ^13^C NMR see Supplementary Table S12 and Figure S22A,B; MS (ESI^+^): [M + Na]^+^, *m/z* 1448.5; [M + 2Na]^2+^, *m/z* 735.8; HRMS (ESI^+^): calcd for C_57_H_87_O_33_N_9_Na^+^: 1448.52985, found 1448.53000 (0.1 ppm), see Supplementary Figure S22C.

### 4.9. Modeling of Enzyme Tunnels and Substrate Docking

The analysis of the access tunnels was performed by Caver 3.0 PyMOL Plugin [29,38], as described previously [39]. Briefly, the coordinates of the *An*Rut dimer crystal structure were downloaded from the RCSB PDB database (http://www.rscb.org/) (PDB ID: 6I1A). Non-protein molecules (10 molecules of *N*-acetyl-β-d-glucosamine, 684 water molecules, and 28 ethan-1,2-diol molecules) and alternative conformations of amino acids were removed from the structures prior to tunnel calculations. The molecular structure was opened using PyMOL 1.7 and the starting point coordinates were set in the position corresponding to 30.969, 29.83, and 38.043 Å. Transport tunnels were identified using a probe radius of 1.0 Å, shell radius of 7.0 Å, shell depth of 4.0 Å, and tunnels were clustered using the threshold of 7.0 and number of approximating balls 20.

The Caverdock method is a computational molecular modeling technique, which is on the verge between geometrical approaches and molecular dynamics simulations. The method offers high-throughput comparative analyses of ligand penetration through multiple protein tunnels. The output is ligand trajectory and energy profile of the transport process. In order to explore the accessibility of substrates rutin and isoquercitrin to the active site of *An*Rut protein, the CAVERDOCK 1.1 [40] tool was applied. This program models the transportation of a substrate from the outside environment into the receptor active or binding site (or vice versa). The input for the calculation is a receptor structure in the PDB format and a ligand structure in the PDBQT format. The outputs are the ligand trajectory in the PDBQT format and the energetic profile of the process. CaverDock implements a novel algorithm based on molecular docking that produces a contiguous ligand trajectory and estimates the binding energy along the pathway. The current version of CaverDock uses CAVER for the pathway identification and a modified Autodock Vina ver. 1.1.2 [41] as the docking engine. The structures of the rutin and isoquercitrin substrates were obtained from the Pubchem database [42] and their molecular geometry was optimized using Chimera [43]. Searches were carried out over the whole molecule allowing ligands to be flexible. The involvement of active site residues in the substrate binding was examined by DS Visualizer ver. 20.1.0.19295 [44], which provided all types of non-covalent interactions in two-dimensional (2D) diagram.

### 4.10. Molecular Dynamics Simulations

Before molecular dynamics simulations, rutin and isoquercitrin substrates were docked into the *An*Rut crystal structure using Autodock Vina tool implemented in Chimera 1.3.1; the size of grid box was as follows: size_x = 19.87 Å, size_y = 22.97 Å, and size_z = 18.56 Å.

Then, the protein molecule was consecutively processed using molecular dynamics software Gromacs 2016.3 [45,46] with OPLS-AA/L all-atom force field. The molecular system was solvated in a cubic water box using SPC/E 3-point water model and the ions were added to neutralize the net charge on the protein. The initial steps in preparing the system for production of molecular dynamics simulations involved energy minimization to find the local energy minimum, adjustment of the particular distribution of solvent molecules, and relaxation of possible steric clashes. In the next step, a short molecular dynamics simulation was performed with harmonic position restraints on the heavy protein atoms with an integration step of 2 fs. There, the solvent and ions around the protein were equilibrated in two phases, the first phase, under 100 ps NVT (constant number of particles, volume, and temperature) ensemble and the second phase under 100 ps NPT (constant number of particles, pressure, and temperature) ensemble. The velocity rescaling thermostat (improvement upon the Berendsen weak coupling method) was used for NVT equilibration, and a Parrinello–Rahman barostat was used for pressure coupling in NPT phase. Electrostatic interactions were estimated using the particle-mesh Ewald (PME) method. Upon completion of both phases, the system was equilibrated at the desired temperature of 300 K and pressure of 1 atm. In the subsequent step, production dynamics were performed where the position restraints were released, and the molecular dynamics trajectories of 100 ns were calculated at a constant temperature of 300 K and a pressure of 1 atm. After visual inspection of the molecular dynamics trajectories, some standard checks regarding quality of the simulations were performed by analysis tools involved in the Gromacs package. The following properties were investigated: root mean square deviation (RMSD) to the X-ray structure and to the average structure; root mean square fluctuations (RMSF) to reproduce flexibility of protein residues (could be compared to crystallographic B-factors); and finally, radius of gyration (Rg) as the measure of the molecular shape and compactions level at each time.

## 5. Conclusions

In the present work, we have demonstrated the application of the solid state catalysis method [17] for the glycosylation of a range of acceptors using both rutin and isoquercitrin as glycosyl donors. The structure–affinity relationships within the studied acceptor series were explained by means of molecular modeling, including the role of inorganic azide as an efficient transglycosylation acceptor. Furthermore, in silico modeling revealed the crucial role of two substrate tunnels in the structure of *An*Rut, unique for the GH5 family and also for the structurally similar exoglucanase enzymes. We hypothesize that especially the side tunnel is the major factor influencing the enzyme synthetic behavior. We have demonstrated the applicability of azide functionalized rutinosides for the construction of multivalent rhamnosyl-capped conjugates that will be further tested as inhibitors of formation of AGE products with potential anti-aging properties.

## Figures and Tables

**Table 1:** Typical isolated yields of transglycosylation products ^a^.

Glycosyl Donor Acceptor	Rutin		Isoquercitrin	
	Product	Yield (%)	Product	Yield (%)
pentanol (**3**)	**10**	16	**17**	15
sodium azide (**4**)	**11**	44	**18**	22
2-azidoethanol (**5**)	**12**	16	**19**	11
2-phenylethanol (**6**)	**13**	9	**20**	7
catechol (**7**)	**14** ^b^	<7	**21**	9
*p*-nitrophenol (**8**)	**15**	7	**22**	8
4-methylumbelliferone (**9**)	**16** ^c^	<5	**23** ^c^	<5

^a^ Products were synthesized and isolated as described in the Materials and Methods Section; ^b^ Data taken from previously published results [13]; ^c^ Products could not be isolated in a sufficient amount for NMR analysis.

**Table 2:** Hydrolysis of transglycosylation products **10**–**23** by *An*Rut ^a^.

Rutinosides	Hydrolysis Rate	β-Glucosides	Hydrolysis Rate
**10**	-	**17**	-
**11**	-	**18**	-
**12**	-	**19**	-
**13**	-	**20**	-
**14**	+	**21**	++
**15**	++	**22**	++

^a^ Total hydrolysis within 4 h, ++; within 24 h, +; no hydrolysis after 48 h, -. The reaction mixtures contained 2 mM transglycosylation product and *An*Rut (0.8 U/mL) in a total volume of 200 µL and were incubated at 36 °C. Samples were analyzed by thin-layer chromatography (TLC; dichloromethane/methanol = 4:1, *v*/*v*) for glucose or rutinose formation.

## Supplementary Materials

The following are available online at https://www.mdpi.com/1422-0067/21/16/5671/s1.

## Abbreviations

*An*Rut	Recombinant rutinosidase from *Aspergillus niger*
GH	Glycoside hydrolase
AGE	Advanced glycation end-products
SDS	Sodium dodecyl sulfate
TLC	Thin-layer chromatography
DMSO	Dimethyl sulfoxide
MS	Mass spectrometry
ESI-MS	Electrospray ionization mass spectrometry
HPLC	High-performance liquid chromatography
NMR	Nuclear magnetic resonance
PDB	Protein data bank
HRMS	High-resolution mass spectrometry
NVT	Constant number of particles, volume, and temperature
NPT	Constant number of particles, pressure, and temperature
RMSD	Root mean square deviation
RMSF	Root mean square fluctuations
