# Supplementary material for: Dual Substrate Specificity of the Rutinosidase from Aspergillus niger and the Role of Its Substrate Tunnel
Source: Int J Mol Sci. 2020 Aug 7;21(16):5671. doi: 10.3390/ijms21165671 (PMC7460883; doi:10.3390/ijms21165671)
Supplement: Supplementary file 1 [file ijms-21-05671-s001.zip › Supplementary materials.pdf]

Supplementary Materials

Dual Substrate Specificity of the Rutinosidase from *Aspergillus niger* and the Role of Its Substrate Tunnel

Katerina Brodsky [1,2], Michal Kutý [3,4], Helena Pelantová [1], Josef Cvačka [5], Martin Rebroš [6], Michael Kotik [1], Ivana Kutá Smatanová [3,4], Vladimír Křen [1] and Pavla Bojarová [1,*]

1 Institute of Microbiology of the Czech Academy of Sciences, Vídeňská 1083, CZ 14220 Prague 4, Czech Republic
2 Department of Biochemistry and Microbiology, University of Chemistry and Technology Prague, Technická 3, CZ 16628 Prague 6, Czech Republic
3 Center for Nanobiology and Structural Biology, Institute of Microbiology of the Czech Academy of Sciences, Zámek 136, CZ 37333 Nové Hrady, Czech Republic
4 Institute of Chemistry, Faculty of Science, University of South Bohemia, Branišovská 1760, CZ 37005 České Budějovice, Czech Republic
5 Institute of Organic Chemistry and Biochemistry of the Czech Academy of Sciences, Flemingovo nám. 2, CZ 16610 Prague 6, Czech Republic
6 Institute of Biotechnology, Slovak University of Technology, Radlinského 9, SK 81237 Bratislava, Slovakia
* Correspondence: bojarova@biomed.cas.cz; Tel.: +420 296442360

Contents:

1. SDS-PAGE and Size Exclusion Chromatography of Recombinant *An*Rut
2. Molecular Modeling
3. Structural Characterization of Compounds

---

1. SDS-PAGE and Size Exclusion Analysis of Recombinant *An*Rut

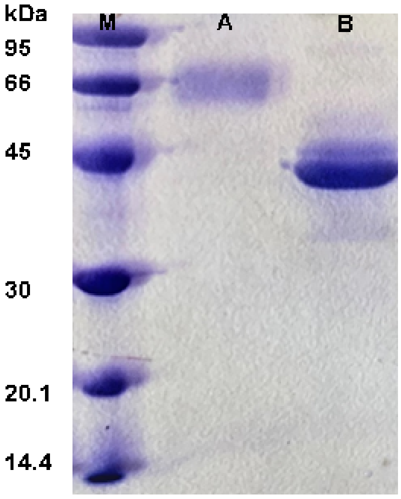

Figure S1. SDS-PAGE analysis of recombinant *An*Rut. Lane A shows recombinant *An*Rut in the native (glycosylated) form; lane B shows spontaneously deglycosylated form of *An*Rut after a longer time period (several months). The deglycosylated *An*Rut has the same activity as the glycosylated enzyme. Lane M shows Low Molecular Weight Protein Marker (GE Healthcare, Chalfont St Giles, UK). Wider bands indicate a mixture of partially glycosylated forms of the enzyme.

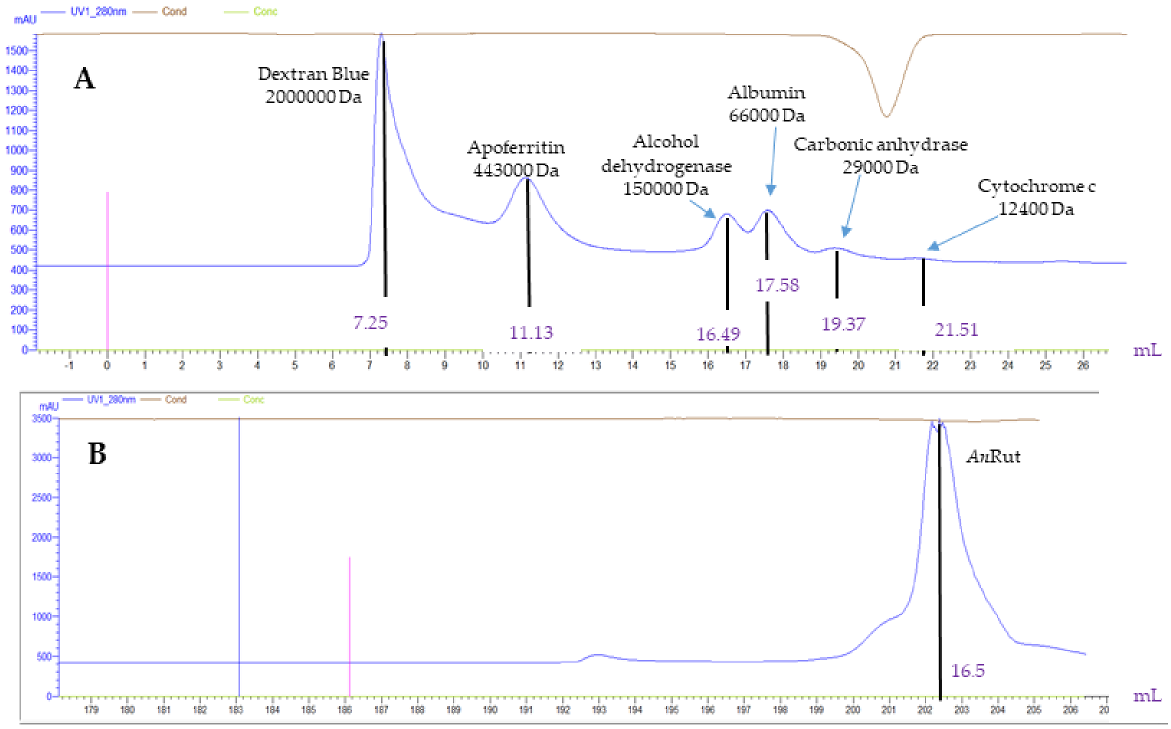

Figure S2. Chromatogram of size exclusion analysis. (**A**), Calibration with molecular weight markers and their elution volumes; (**B**), determination of native deglycosylated *An*Rut enzyme. *An*Rut is a dimer composed of two identical catalytic subunits (calculated molar weight of 87.1 kDa).

2. Molecular Modeling

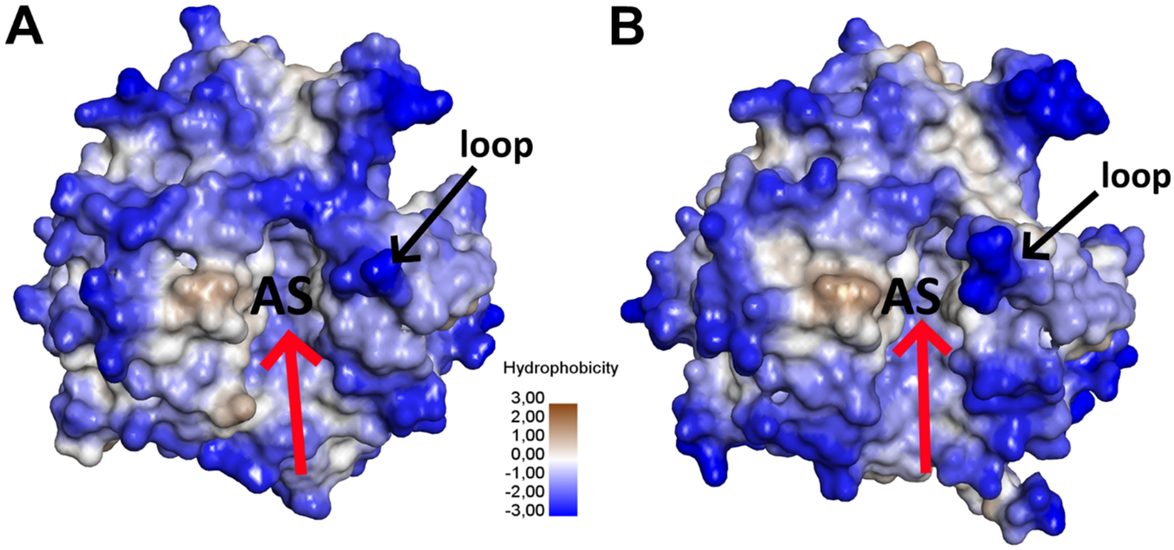

Figure S3. (**A**), *Ca*Exg protein represented by surface model colored by hydrophobicity with a deep valley (red arrow) and a large flexible loop (black arrow); (**B**), *Sc*Exg protein represented by surface model colored by hydrophobicity with a deep valley (red arrow) and a large flexible loop (black arrow).

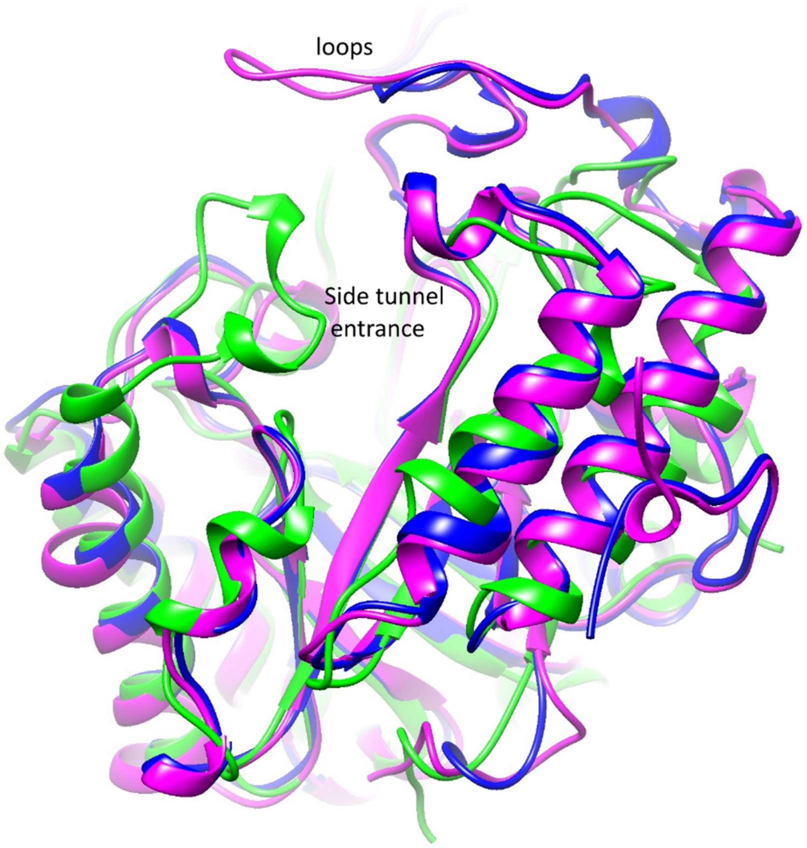

Figure S4. The superposition of all three protein crystal structures in ribbon models (*An*Rut in green, *Ca*Exg in blue and *Sc*Exg in magenta) reveals their structural similarity and differences. The main difference is detected at the side tunnel entrance, where the loop (in green) of *An*Rut forms the tunnel in contrast to the other two proteins, which form a large valley at this site.

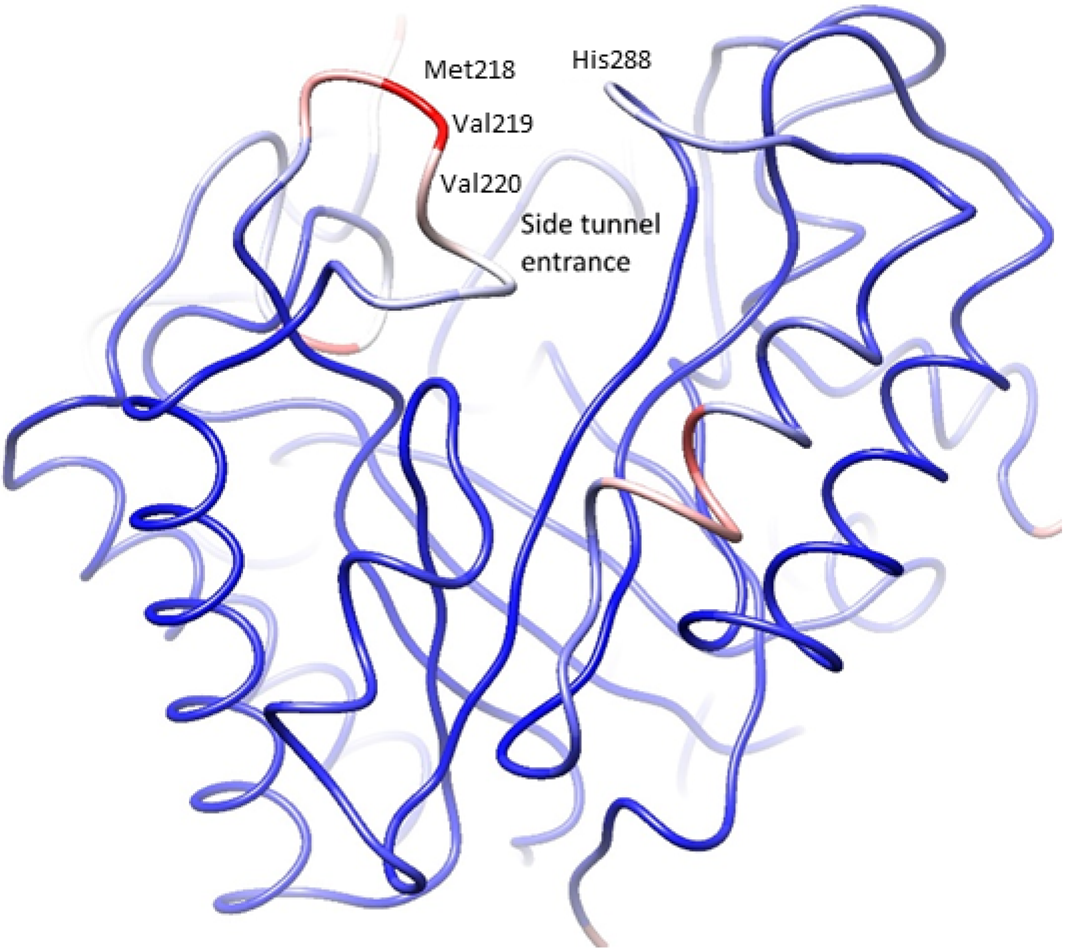

Figure S5. The ribbon model of *An*Rut protein after 100 ns of molecular dynamics simulations. The ribbon is colored according to the root mean square fluctuation (RMSF) analysis to reproduce the flexibility of protein residues (may be compared to crystallographic B-factors). The flexible protein areas are colored in red, the least flexible in blue. The most flexible part was detected within the side tunnel part (loop Asn215-Pro224); nevertheless, during the whole simulation time the tunnel remained preserved.

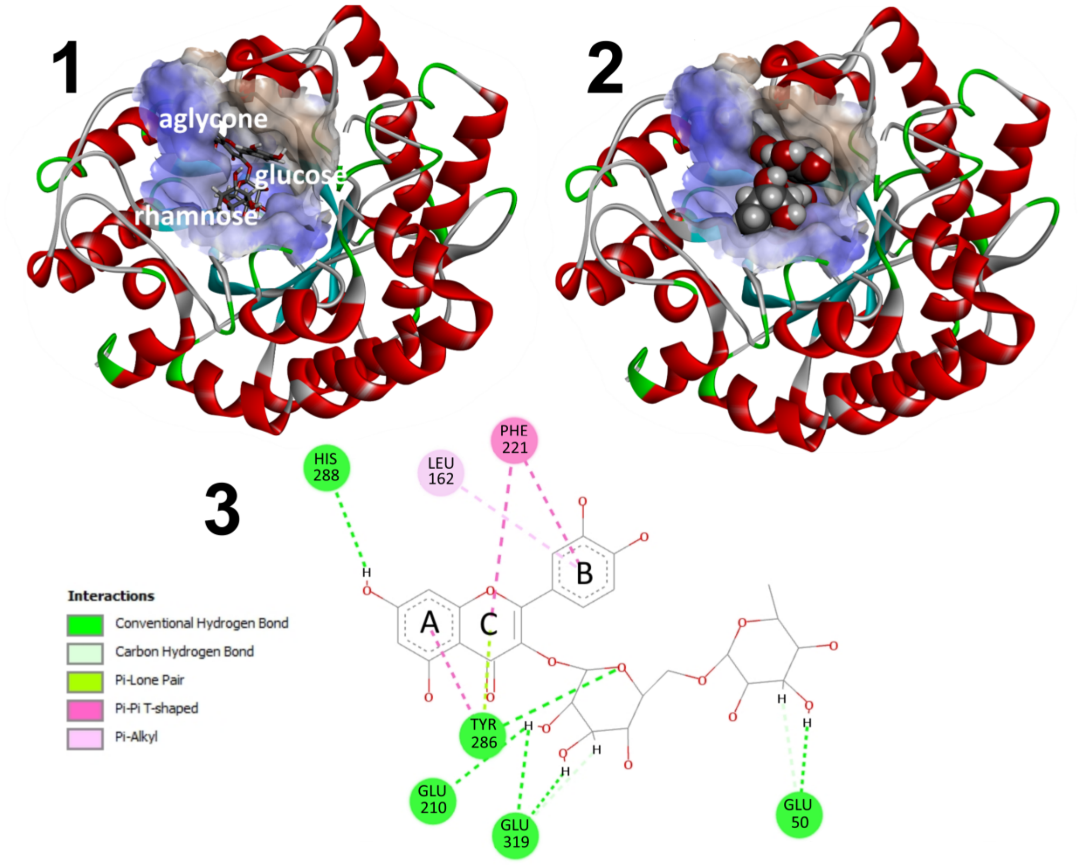

Figure S6. The *An*Rut-rutin complex (in the hydrophobic pocket of active site) obtained by the substrate transport from protein surface into the receptor active site near the catalytic acid/base residue Glu210. The most favorable binding mode (energy score -8.3 kcal/mol) of rutin is deeply inserted in the hydrophobic part of side tunnel by the main part of aglycone (rings A, C) unit. (**1**), Rutin is shown as a stick model, and *An*Rut as a ribbon model; (**2**), rutin is shown as a CPK model to provide a more realistic impression of rutin molecular size in the hydrophobic pocket of the active site, and *An*Rut is shown as a ribbon model; (**3**), the 2D diagram of non-covalent interactions in *An*Rut-rutin complex. Polar interactions, hydrogen bonds, π-alkyl, π-lone pair and π-π T-shaped interactions in the modeled complex are displayed. π-Interactions are present only between the aglycone and the *An*Rut sidechains, where the aglycone is clamped by Leu162 and by two aromatic sidechains of Phe221 and Tyr286, building π-interactions with two ring structures of the bound quercetin moiety. Additionally, the aglycone (specifically, the A ring of quercetin) forms H-bond with His288, which significantly participate in the formation of the side tunnel. The glucose subunit forms four strong H-bond interactions with Glu210, Glu319, and Tyr286 residues. The rhamnose unit of rutin is least important for substrate binding as it forms only one H-bond with Glu50 residue.

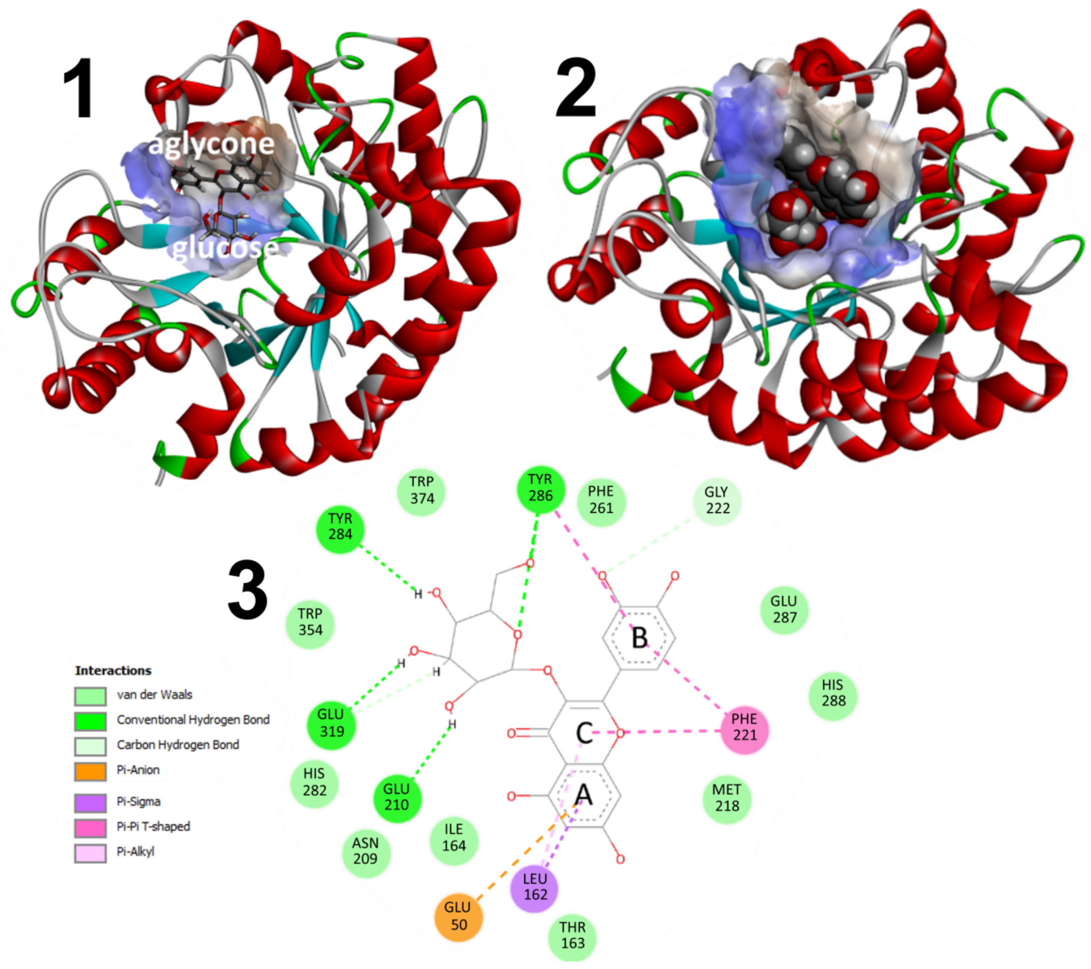

Figure S7A. The most favorable mode of binding of the *An*Rut-isoquercitrin complex (in hydrophobic pocket of active site) obtained by the substrate transport from the protein surface into the receptor active site near the catalytic acid/base residue Glu210. In this binding mode (energy score −8.4 kcal/mol), isoquercitrin is deeply inserted in the hydrophobic part of side tunnel by the hydroxyphenyl part of isoquercitrin aglycone (B ring) unit. (**1**), Isoquercitrin is shown as a stick model in the hydrophobic pocket of the active site within the *An*Rut ribbon model; (**2**), isoquercitrin is viewed by CPK model to provide a more realistic impression of rutin molecular size in hydrophobic pocket of the active site within the *An*Rut ribbon model; (**3**), the 2D diagram of non-covalent interactions in *An*Rut- isoquercitrin complex with the most favorable binding mode. Polar interactions, hydrogen bonds, π-alkyl, π-lone pair and π-π T-shaped interactions in the modeled complex are displayed. The π-interactions with Phe221, Tyr286, Glu50 and Leu162 residues are present only between the aglycone and the *An*Rut sidechains whereas the glucose unit forms five significant H-bonds with Glu210, Glu319, Tyr284, and Tyr286 residues. Both the aglycone and the glucosyl moiety appear to contribute to the binding of substrate to the *An*Rut active site.

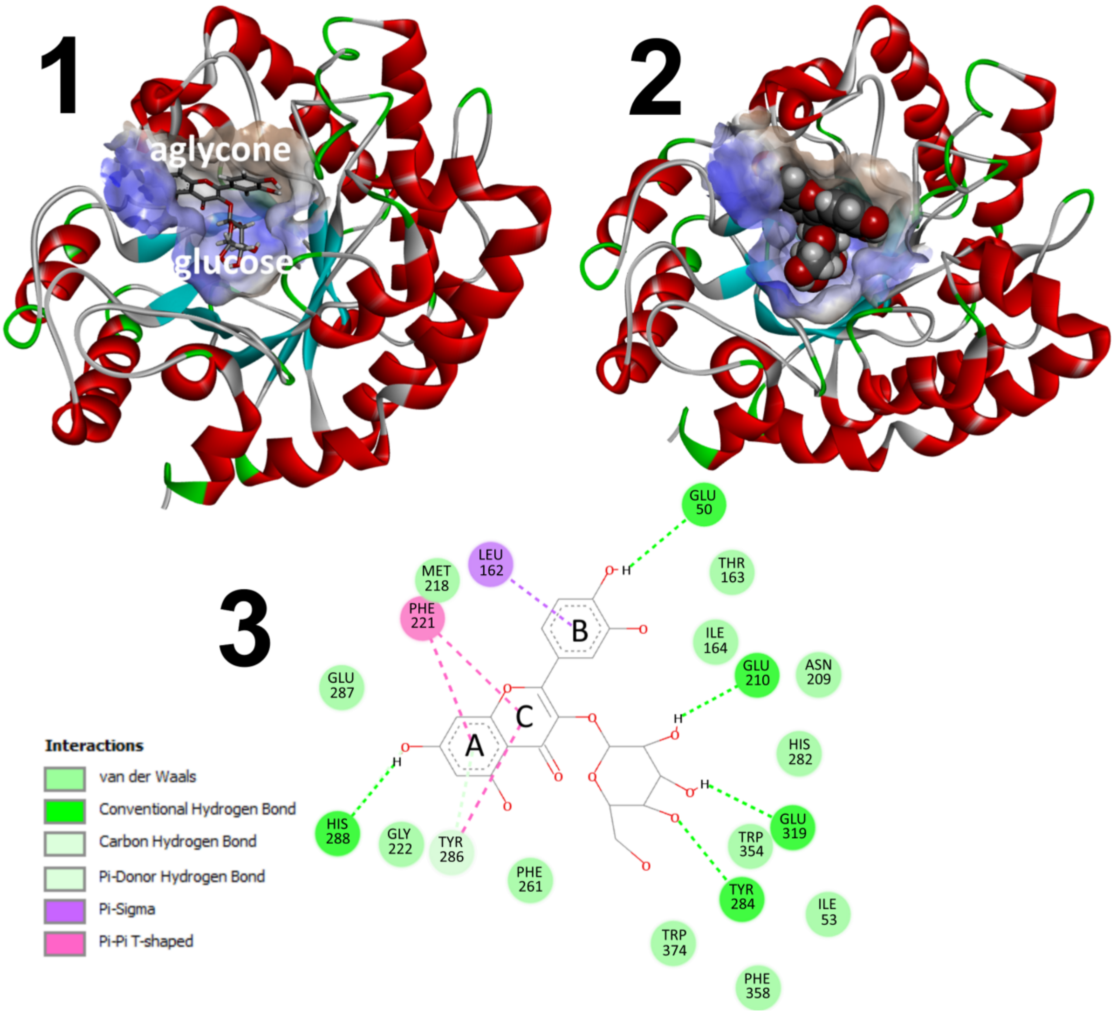

Figure S7B. The second most favorable binding mode of the *An*Rut-isoquercitrin complex obtained by the substrate transport from the protein surface into the receptor active site near the catalytic acid/base residue Glu210. In this binding mode (energy score -8.0 kcal/mol), isoquercitrin is deeply inserted in the hydrophobic part of side tunnel by the main part of aglycone (rings A, C) unit similar to the rutin binding mode. (**1**), Isoquercitrin is shown as a stick model in the hydrophobic pocket of the active site within the *An*Rut ribbon model; (**2**), isoquercitrin is shown as a CPK model to provide a more realistic impression of rutin molecular size in the hydrophobic pocket of the active site within the *An*Rut ribbon model; (**3**), the 2D diagram of non-covalent interactions in *An*Rut-isoquercitrin complex with the second most favorable binding mode. Polar interactions, hydrogen bonds, π-alkyl, π-lone pair and π-π T-shaped interactions in the modeled complex are displayed. π-Interactions are present only between the aglycone and the *An*Rut sidechains, where the aglycone is clamped by Leu162 and by two aromatic sidechains of Phe221 and Tyr286. Additionally, the A ring of quercetin aglycone forms H-bond with His288, and the B ring of quercetin forms H-bond with the Glu50. The glucose subunit forms three strong H-bond interactions with Glu210, Glu319, and Tyr286 residues.

3. Structural Characterization of Compounds

Table S1. ^{1}H and ^{13}C NMR data of product pentyl rutinoside (**10**) (399.87 MHz for ^{1}H, 100.55 MHz for ^{13}C, CD_3OD, 30 °C).

	Atom	δ_C	m.	δ_H	n_H	m.	*J*[Hz]	Crucial HMBC
**Glc**	1	104.47	D	4.224	1	d	7.8	1″
	2	75.16	D	3.156	1	dd	9.1, 7.8	
	3	78.18	D	3.333	1	dd	9.1, 8.8	
	4	71.79	D	3.259	1	dd	9.6, 8.8	
	5	76.89	D	3.384	1	ddd	9.6, 6.2, 1.9	
	6	68.27	T	3.966	1	dd	11.3, 1.9	1′
				3.612	1	dd	11.3, 6.2	
**Rha**	1′	102.35	D	4.743	1	dm	1.8	6
	2′	72.27	D	3.826	1	dd	3.4, 1.8	
	3′	72.44	D	3.659	1	dd	9.5, 3.4	
	4′	74.09	D	3.360	1	dd	9.5, 9.5	
	5′	69.84	D	3.665	1	ddq	9.5, 0.5, 6.2	
	6′	18.08	Q	1.258	3	d	6.2	
**aglycone**	1″	71.00	T	3.838	1	dt	9.6, 6.8	1
				3.533	1	dt	9.6, 6.8	
	2″	30.56	T	1.628	2	m		
	3″	29.34	T	1.359	2	m		
	4″	23.59	T	1.359	2	m		
	5″	14.42	Q	4.254	3	t	7.1	

Glc-glucosyl moiety; Rha- rhamnosyl moiety.

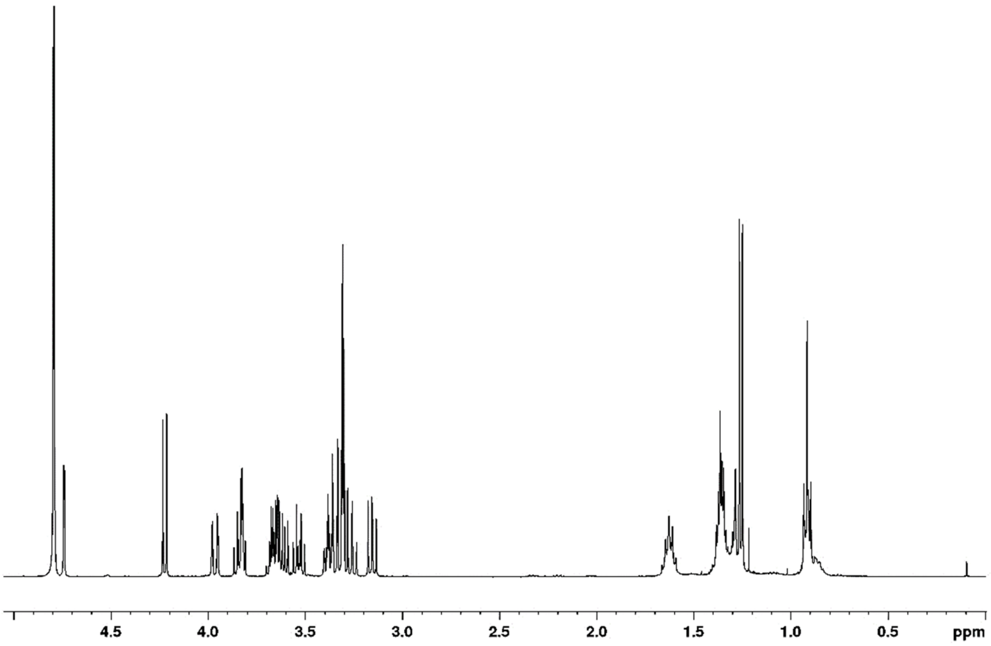

Figure S8A. ^{1}H NMR spectrum of pentyl rutinoside (**10**) (399.87 MHz for ^{1}H, CD_3OD, 30 °C).

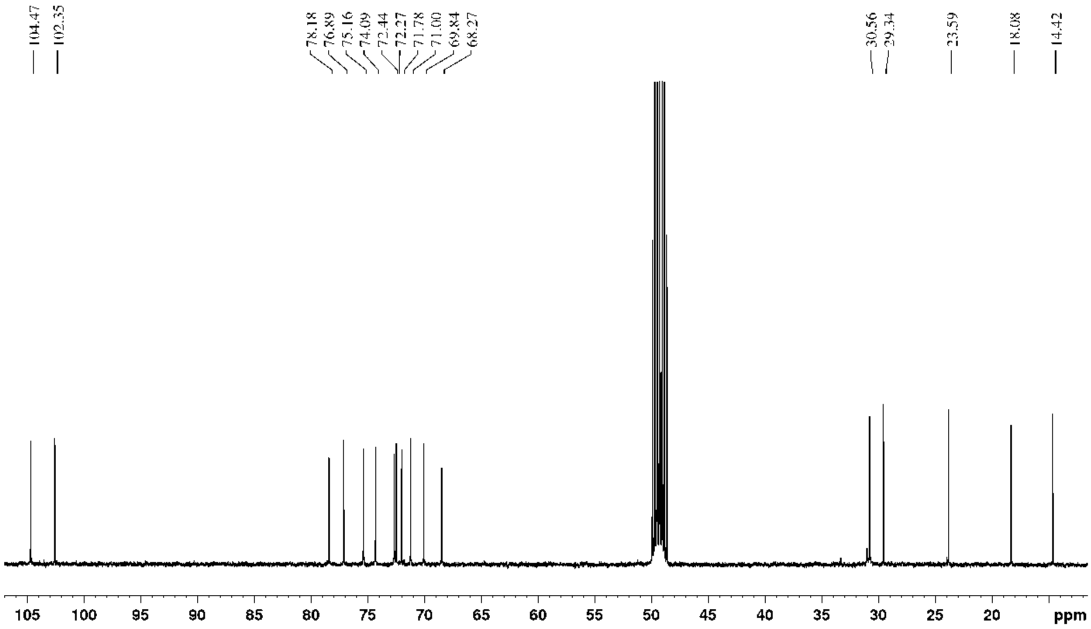

Figure S8B. ^{13}C NMR spectrum of pentyl rutinoside (**10**) (100.55 MHz for ^{13}C, CD_3OD, 30 °C).

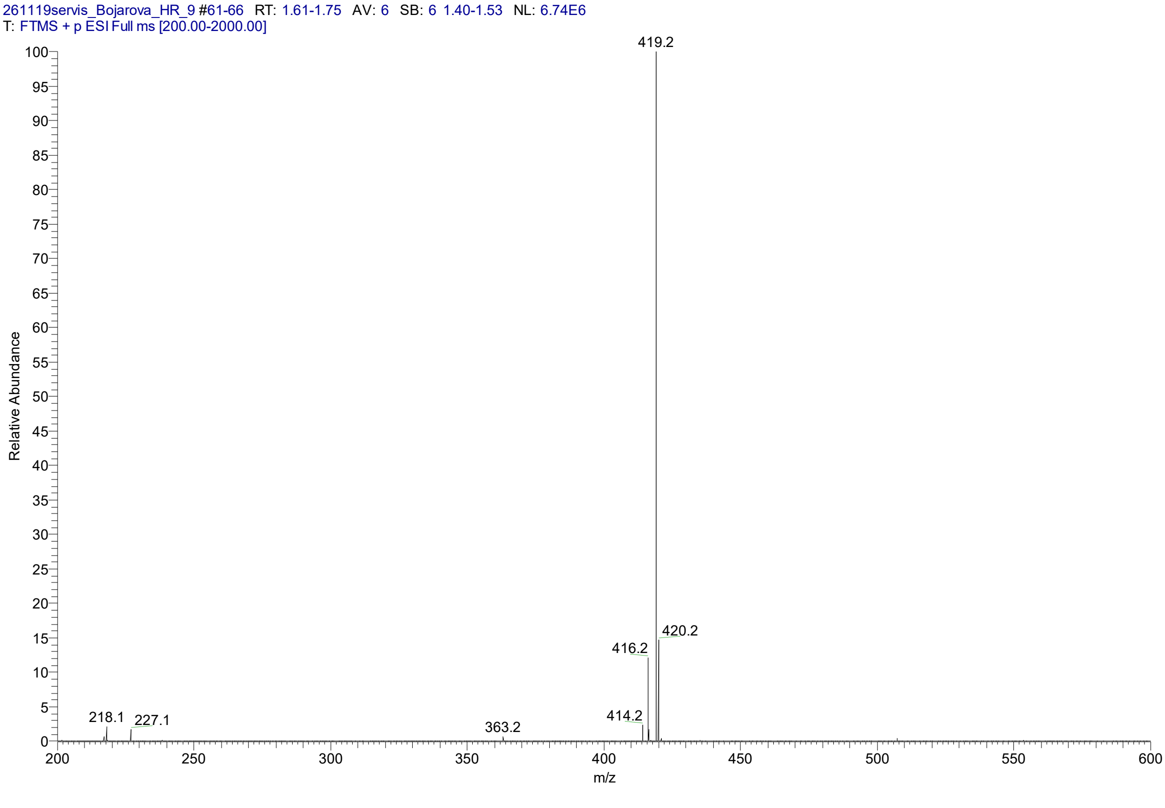

Figure S8C. ESI$^+$ MS spectrum of pentyl rutinoside (**10**) ([M + Na]$^+$, *m/z* 419.2).

Table S2. ^{1}H and ^{13}C NMR data of rutinosyl azide (**11**) (399.87 MHz for ^{1}H, 100.55 MHz for ^{13}C, D_2O, 30 °C).

	Atom	δ_C	m.	δ_H	n_H	m.	J[Hz]
**Glc**	**1**	90.43	D	4.745	1	d	8.7
	**2**	73.01	D	3.286	1	dd	9.3, 8.7
	**3**	75.93	D	3.529	1	dd	9.3, 9.0
	**4**	69.57	D	3.446	1	dd	9.6, 9.0
	**5**	77.03	D	3.678	1	ddd	9.6, 6.0, 1.8
	**6**	66.99	T	4.026	1	dd	11.4, 1.8
				3.732	1	dd	11.4, 6.0
**Rha**	**1′**	100.82	D	4.836	1	dm	1.8
	**2′**	70.26	D	3.999	1	dd	3.5, 1.8
	**3′**	70.46	D	3.796	1	dd	9.7, 3.5
	**4′**	72.30	D	3.451	1	dd	9.7, 9.6
	**5′**	69.01	D	3.752	1	ddq	9.6, 0.6, 6.3
	**6′**	16.90	Q	1.310	3	d	6.3

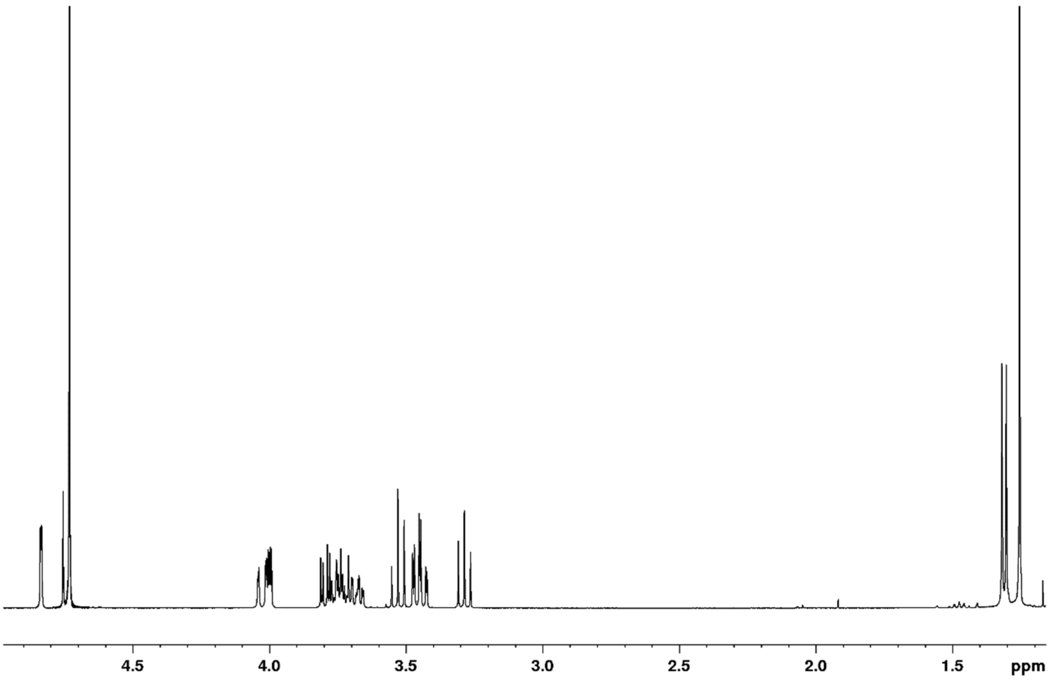

Figure S9A. ^{1}H NMR spectrum of rutinosyl azide (**11**) (399.87 MHz for ^{1}H, D_2O, 30 °C).

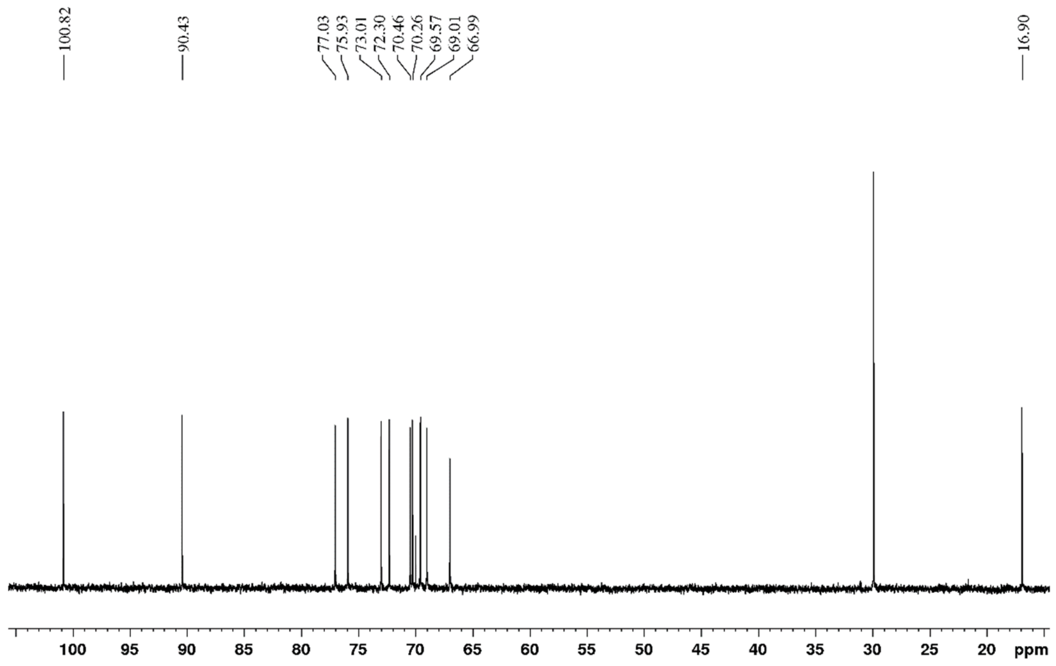

Figure S9B. ^{13}C NMR spectrum of rutinosyl azide (**11**) (100.55 MHz for ^{13}C, D_2O, 30 °C).

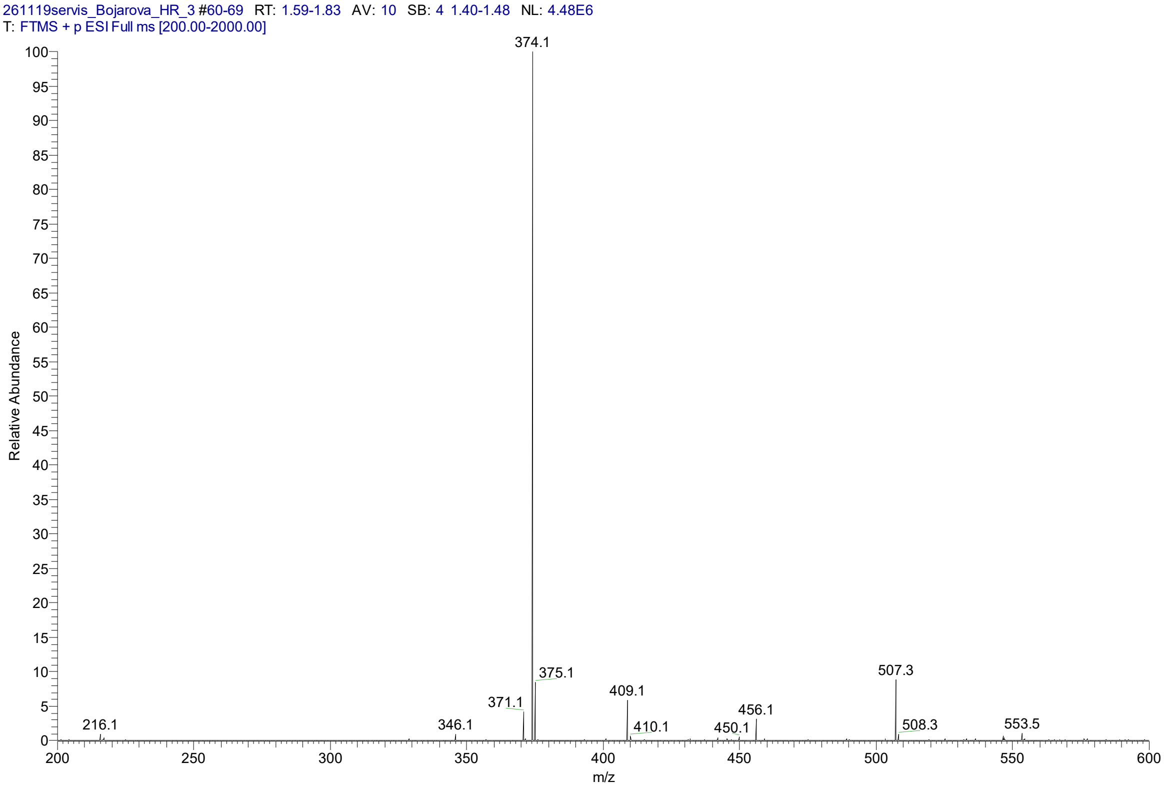

Figure S9C. ESI$^+$ MS spectrum of rutinosyl azide (**11**) ([M + Na]$^+$, *m/z* 374.1).

Table S3. ^{1}H and ^{13}C NMR data of 2-azidoethyl rutinoside (**12**) (600.23 MHz for ^{1}H, 150.93 MHz for ^{13}C, D_2O, 30 °C).

	Atom	δ_C	m.	δ_H	n_H	m.	*J*[Hz]	Crucial HMBC
**Glc**	**1**	102.77	D	4.551	1	d	8.0	1″
	**2**	73.31	D	3.362	1	dd	9.4, 8.0	
	**3**	75.96	D	3.554	1	dd	9.4, 9.0	
	**4**	69.96	D	3.476	1	dd	10.0, 9.0	
	**5**	75.10	D	3.644	1	ddd	10.0, 6.1, 1.9	
	**6**	67.12	T	4.076	1	dd	11.6, 1.9	1′
				3.760	1	dd	11.6, 6.1	
**Rha**	**1′**	100.90	D	4.889	1	dm	1.8	6
	**2′**	70.33	D	4.039	1	dd	3.5, 1.8	
	**3′**	70.50	D	3.847	1	dd	9.7, 3.5	
	**4′**	72.34	D	3.499	1	dd	9.7, 9.6	
	**5′**	68.95	D	3.797	1	dq	9.6, 6.3	
	**6′**	16.89	Q	1.355	3	d	6.3	
**aglycone**	**1″**	68.89	T	4.083	1	ddd	11.4, 6.1, 3.6	1
				3.919	1	ddd	11.4, 6.7, 3.7	
	**2″**	50.82	T	3.609	2	m	-	

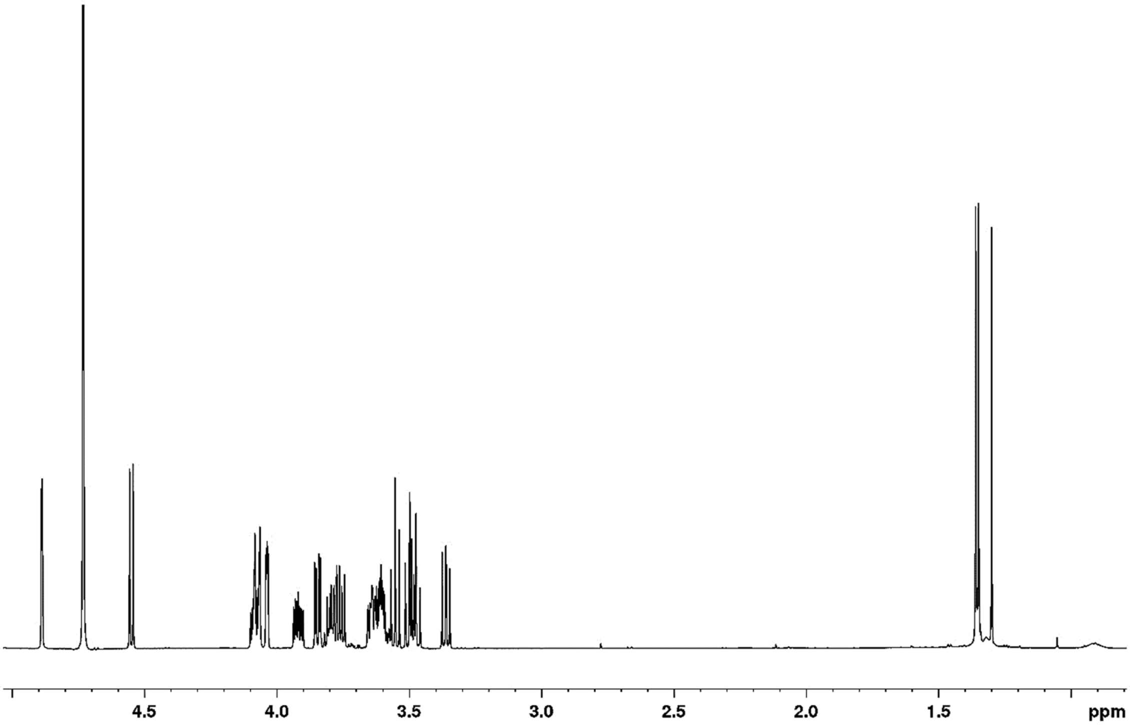

Figure S10A. ^{1}H NMR spectrum of 2-azidoethyl rutinoside (**12**) (600.23 MHz for ^{1}H, D_2O, 30 °C).

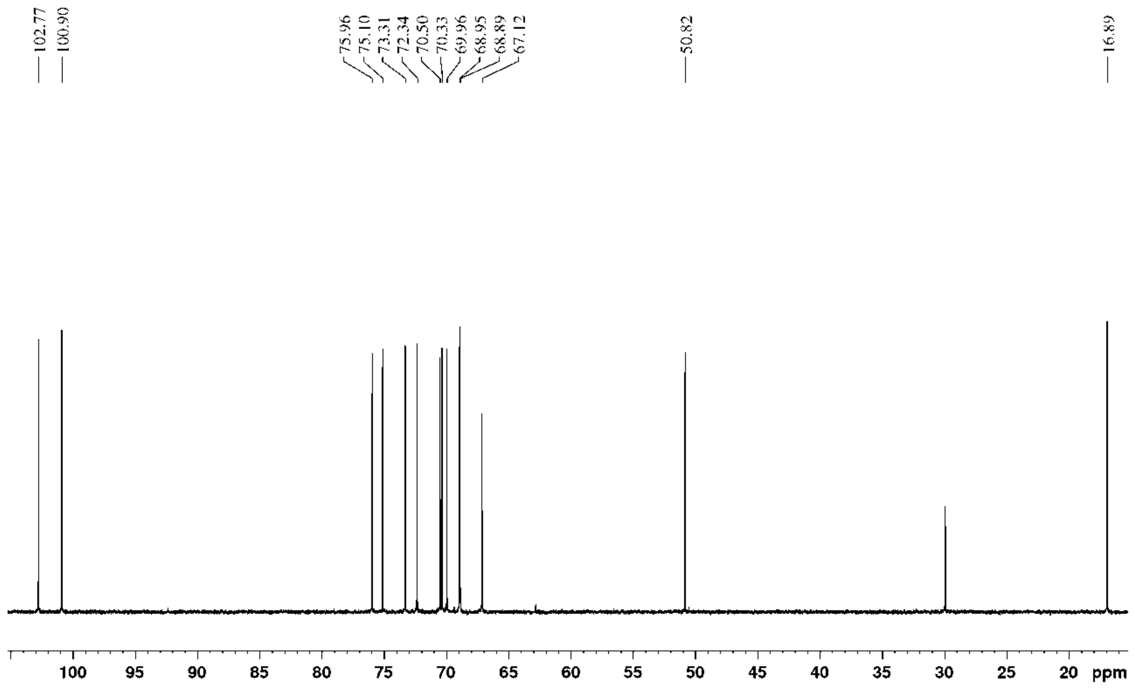

Figure S10B. ^{13}C NMR spectrum of 2-azidoethyl rutinoside (**12**) (150.93 MHz for ^{13}C, D_2O, 30 °C).

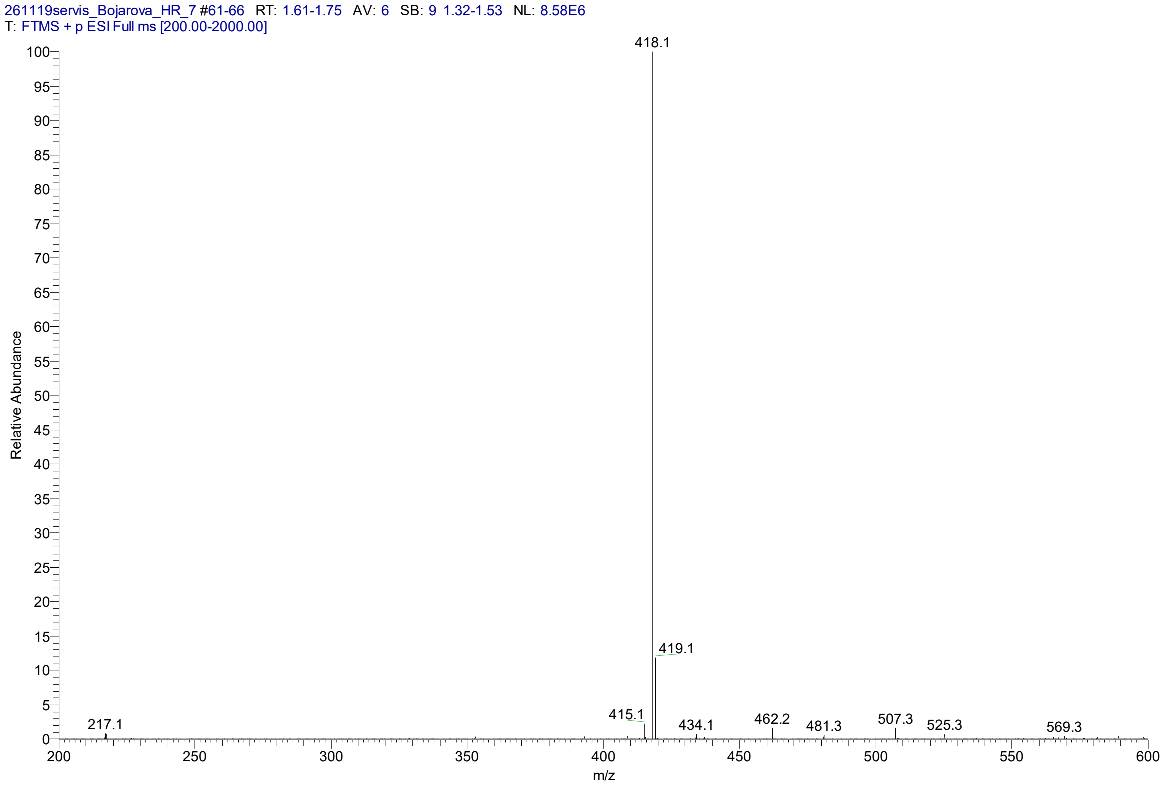

Figure S10C. ESI$^+$ MS spectrum 2-azidoethyl rutinoside (**12**) ([M + Na]$^+$, *m/z* 418.1).

Table S4. ^{1}H and ^{13}C NMR data of 2-phenylethyl rutinoside (**13**) (399.87 MHz for ^{1}H, 100.55 MHz for ^{13}C, D_2O, 30 °C).

	Atom	δ_C	m.	δ_H	n_H	m.	*J*[Hz]	Crucial HMBC
**Glc**	**1**	102.62	D	4.461	1	d	8.0	1″, 2″
	**2**	73.29	D	3.253	1	dd	9.3, 8.0	
	**3**	75.97	D	3.471	1	dd	9.3, 9.1	
	**4**	69.97	D	3.391	1	dd	9.7, 9.1	
	**5**	75.00	D	3.553	1	ddd	9.7, 6.2, 2.0	
	**6**	67.15	T	3.999	1	dd	11.6, 2.0	1′
				3.695	1	dd	11.6, 6.2	
**Rha**	**1′**	100.87	D	4.834	1	dm	1.8	6
	**2′**	70.33	D	3.977	1	dd	3.5, 1.8	
	**3′**	70.47	D	3.792	1	dd	9.8, 3.5	
	**4′**	72.31	D	3.444	1	dd	9.8, 9.5	
	**5′**	68.94	D	3.754	1	dq	9.8, 6.3	
	**6′**	16.93	Q	1.295	3	d	6.3	
**aglycone**	**1″**	71.17	T	4.130	1	ddd	10.2, Σ*J* = 13.8	1
				3.941	1	ddd	10.2, Σ*J* = 14.0	
	**2″**	35.54	T	2.981	2	dd	Σ*J* = 13.9	
	ipso-	138.98	S	-	0	-		1″, 2″
	ortho-	129.27	D	7.375	2	m		2″
	meta-	128.95	D	7.400	2	m		
	para-	126.84	D	7.312	1	m		

[a] … HSQC readout.

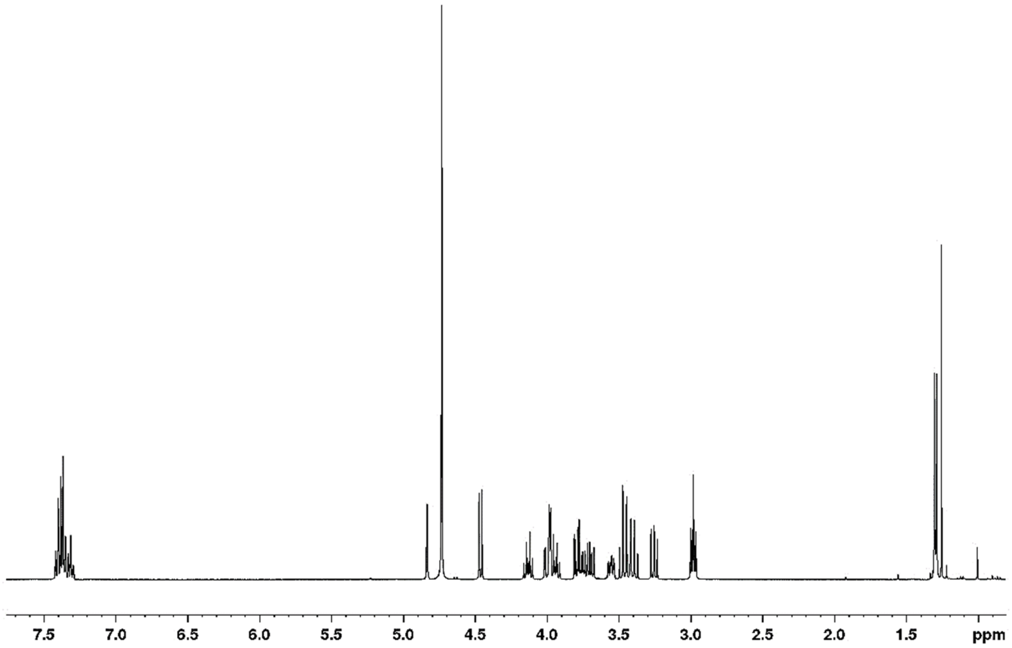

Figure S11A. ^{1}H NMR spectrum of 2-phenylethyl rutinoside (**13**) (399.87 MHz for ^{1}H, D_2O, 30 °C).

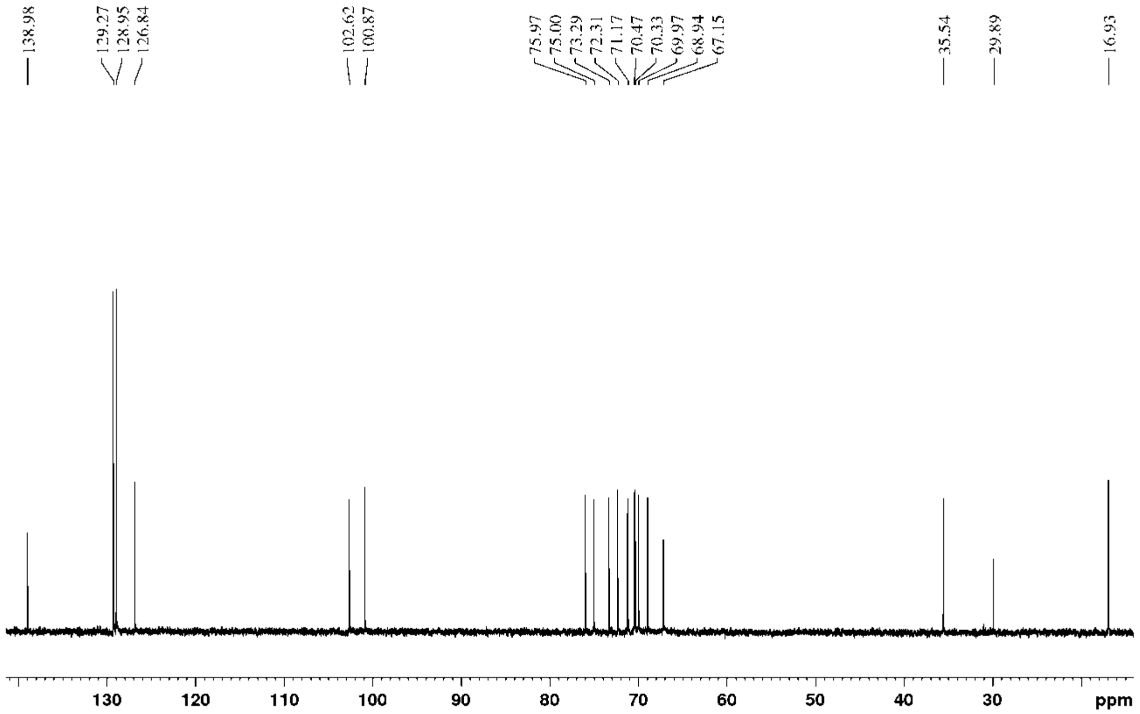

Figure S11B. ^{13}C NMR spectrum of 2-phenylethyl rutinoside (**13**) (100.55 MHz for ^{13}C, D_2O, 30 °C).

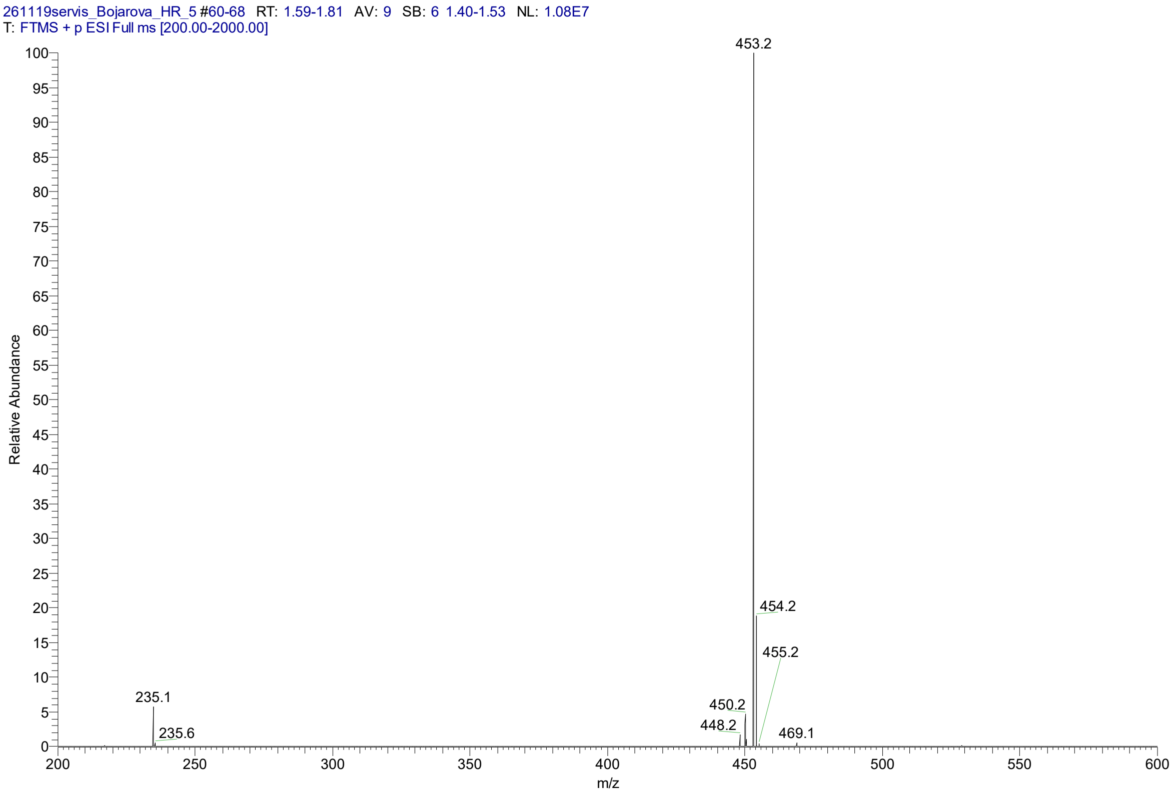

Figure S11C. ESI$^+$ MS spectrum of 2-phenylethyl rutinoside (**13**) ([M + Na]$^+$, *m/z* 453.2).

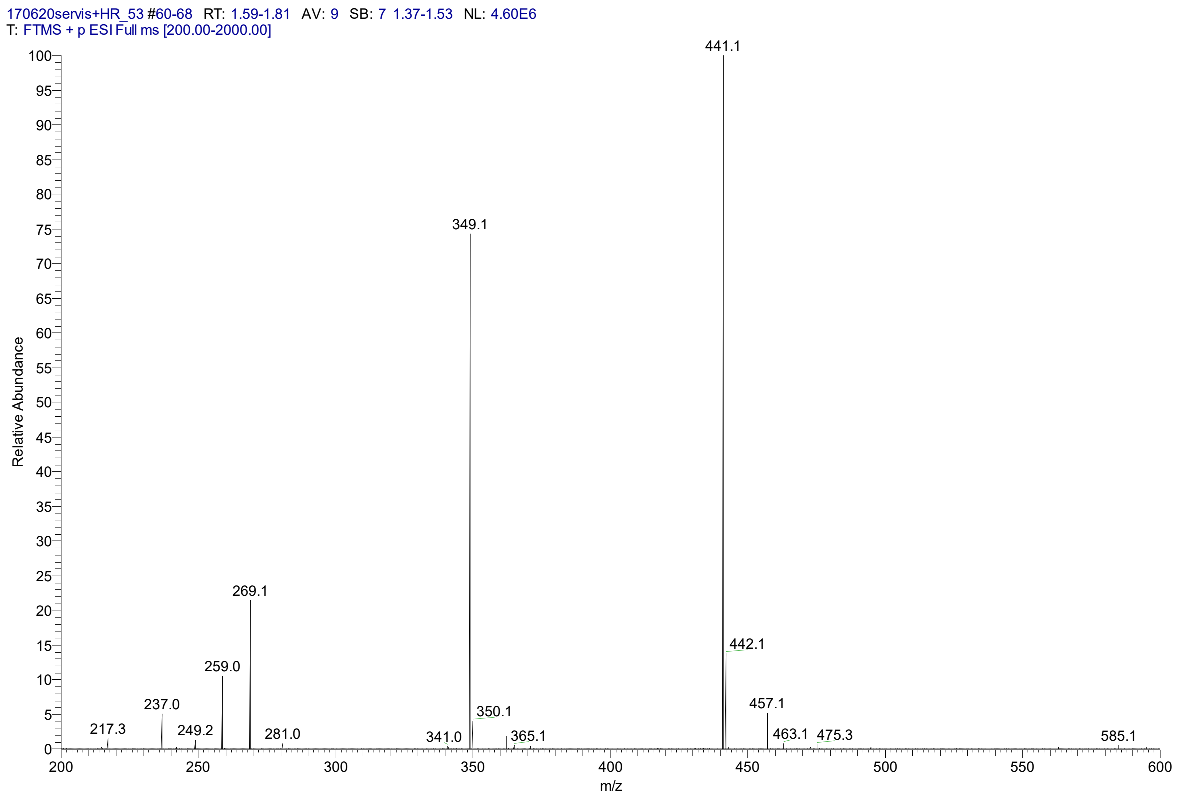

Figure S12. ESI+ MS spectrum of catechol rutinoside (**14**) ([M + Na]+, *m/z* 441.1).

Table S5. ^{1}H and ^{13}C NMR data of *p*-nitrophenol rutinoside (**15**) (399.87 MHz for ^{1}H, 100.55 MHz for ^{13}C, D_2O, 30 °C).

	Atom	δ_C	m.	δ_H	n_H	m.	*J*[Hz]	Crucial HMBC
**Glc**	**1**	99.46	D	5.307	1	m		
	**2**	73.03	D	3.66 [a]	1	m		
	**3**	75.70	D	3.66 [a]	1	m		
	**4**	69.71	D	3.565	1	m		
	**5**	75.34	D	3.835	1	ddd	9.7, 6.0, 2.1	
	**6**	66.35	T	4.047	1	dd	11.6, 2.1	1′
				3.745	1	dd	11.6, 6.0	
**Rha**	**1′**	100.46	D	4.780	1	d	1.8	6
	**2′**	70.30	D	3.922	1	dd	3.5, 1.8	
	**3′**	70.52	D	3.784	1	dd	9.7, 3.5	
	**4′**	72.28	D	3.415	1	dd	9.7, 9.6	
	**5′**	68.93	D	3.717	1	dq	9.6, 6.3	
	**6′**	16.73	Q	1.176	3	d	6.3	
**aglycone**	***ipso-***	161.85	S	-	0	-		
	ortho-	116.77	D	7.265	2	m		
	meta-	126.38	D	8.299	2	m		
	para-	142.94	S	-	0			

[a] … HSQC readout.

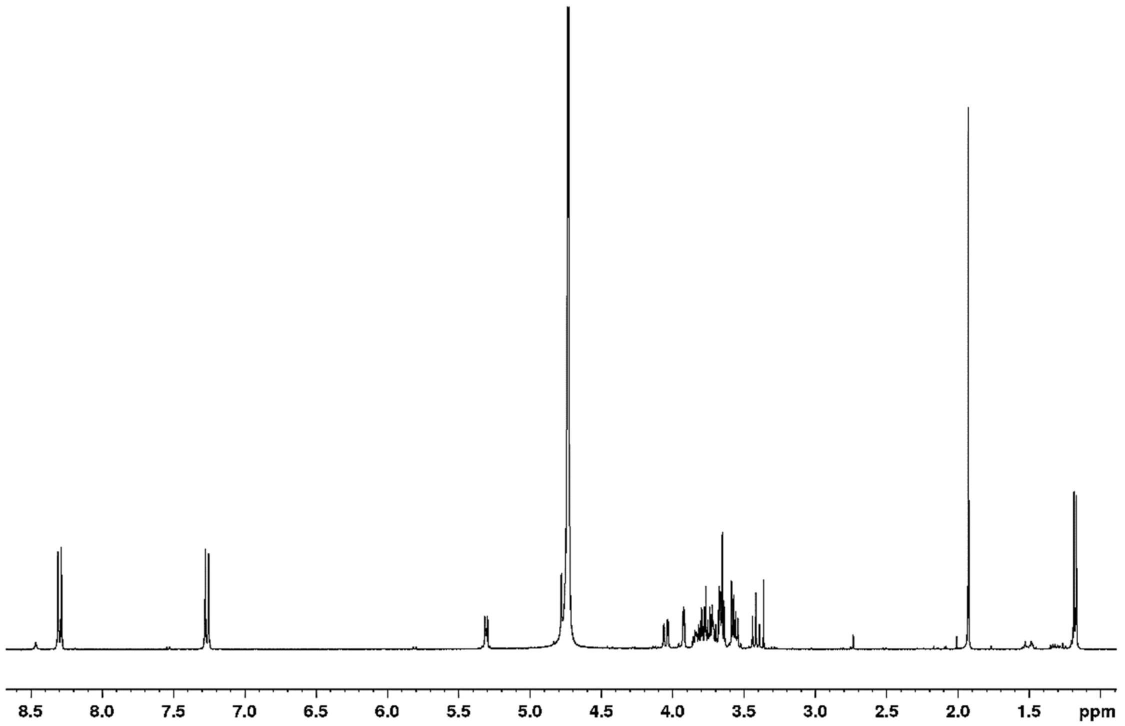

Figure S13A. ^{1}H NMR spectrum of *p*-nitrophenyl rutinoside (**15**) (399.87 MHz for ^{1}H, D_2O, 30 °C).

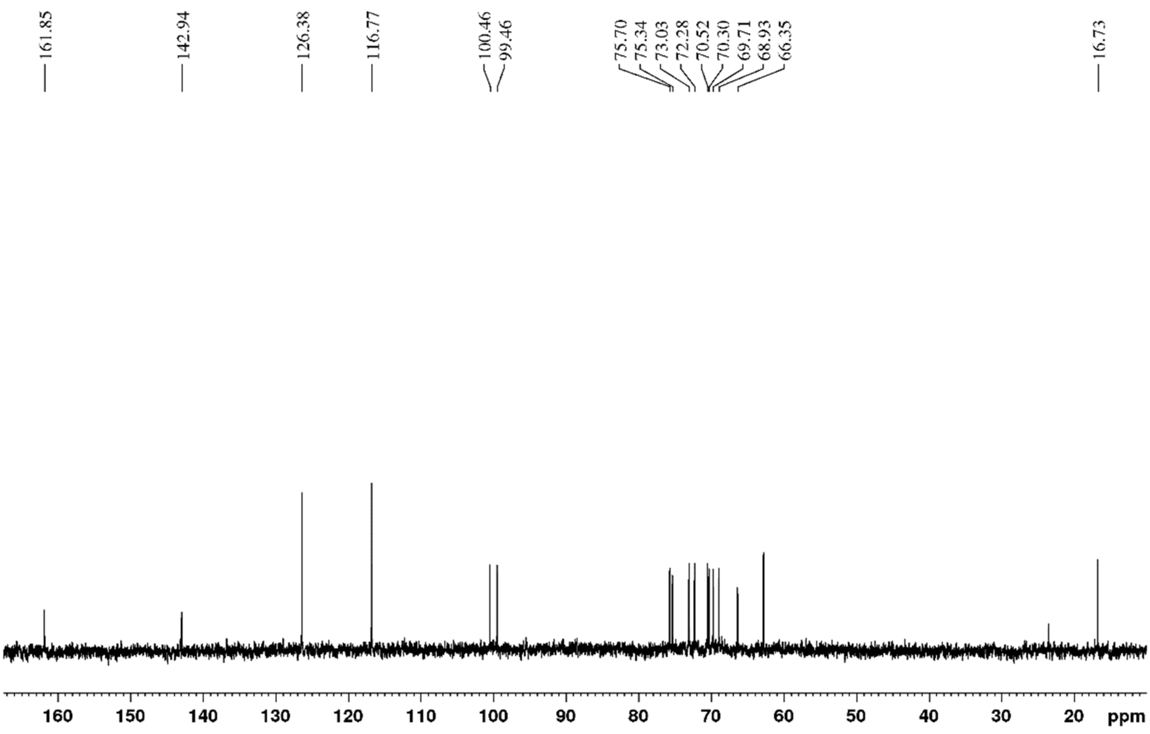

Figure S13B. ^{13}C NMR spectrum of *p*-nitrophenol rutinoside (**15**) (100.55 MHz for ^{13}C, D_2O, 30 °C).

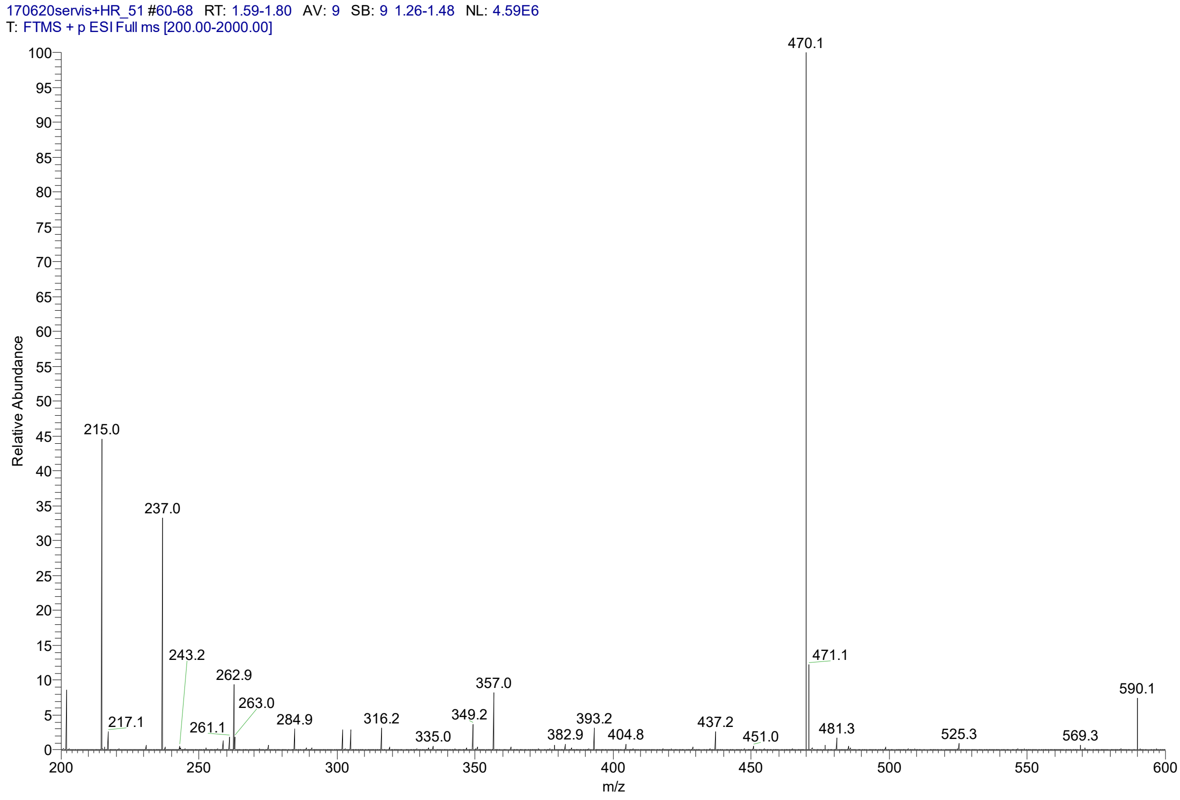

Figure S13C. ESI$^+$ MS spectrum of *p*-nitrophenyl rutinoside (**15**) ([M + Na]$^+$, *m/z* 470.1).

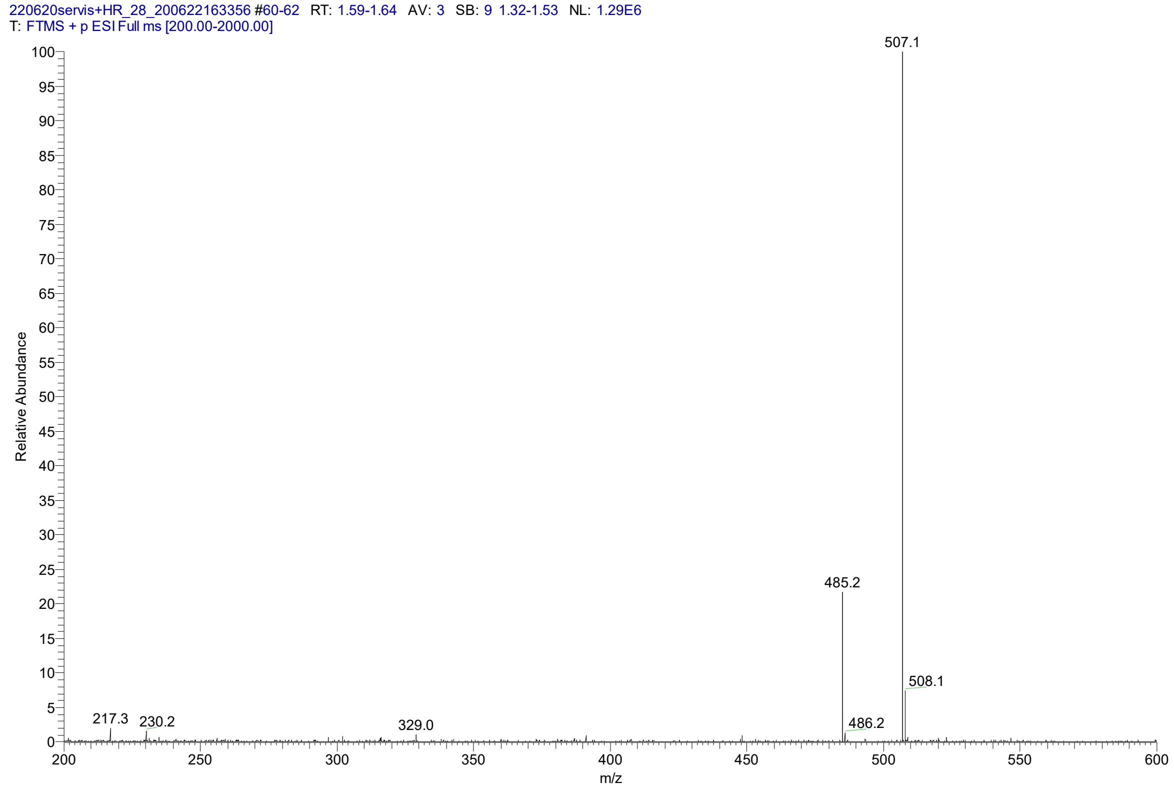

Figure S14. ESI$^+$ MS spectrum of 4-methylumbelliferyl rutinoside (**16**) ([M + H]$^+$, *m/z* 485.2; [M + Na]$^+$, *m/z* 507.1).

Table S6. ^{1}H and ^{13}C NMR data of pentyl glucoside (**17**) (399.87 MHz for ^{1}H, 100.55 MHz for ^{13}C, D_2O, 30 °C).

	Atom	δ_C	m.	δ_H	n_H	m.	*J*[Hz]
**Glc**	**1**	102.44	D	4.456	1	d	8.0
	**2**	73.44	D	3.260	1	dd	9.3, 8.0
	**3**	76.12	D	3.488	1	dd	9.3, 8.9
	**4**	69.96	D	3.382	1	dd	9.8, 8.9
	**5**	76.16	D	3.451	1	ddd	9.8, 5.7, 2.2
	**6**	61.07	T	3.918	1	dd	12.3, 2.2
				3.722	1	dd	12.3, 5.7
**aglycone**	**1′**	70.96	T	3.920	1	dt	9.9, 6.9
				3.679	1	dt	9.9, 6.9
	**2′**	28.21	T	1.634	2	m	
	**3′**	27.59	T	1.33[a]	2	m	
	**4′**	22.01	T	1.33[a]	2	m	
	**5′**	13.52	Q	0.890	3	m	

[a] … HSQC readout.

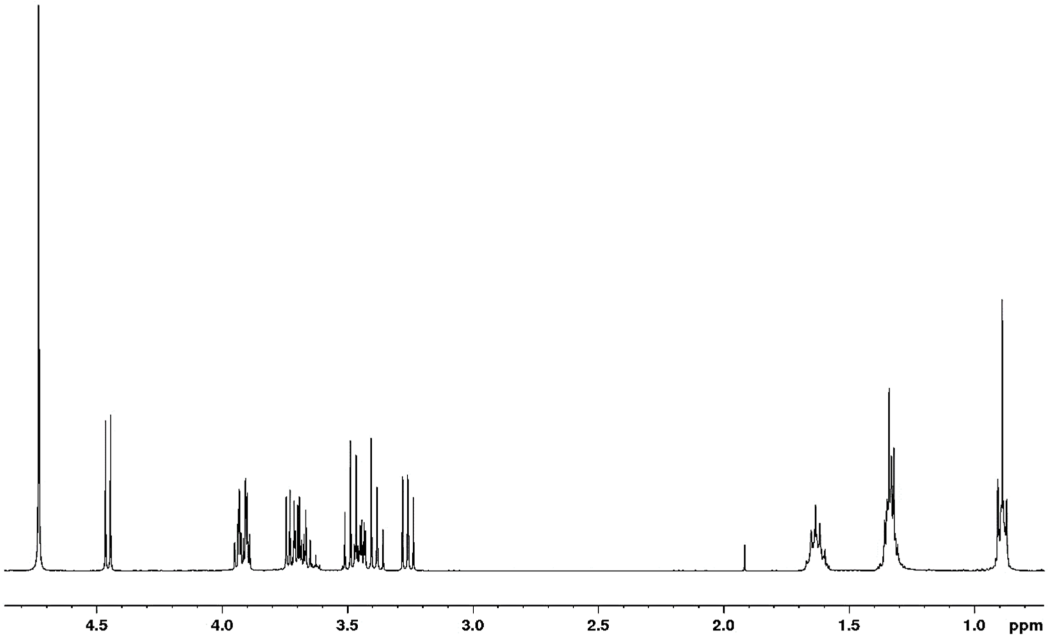

Figure S15A. ^{1}H NMR spectrum of pentyl glucoside (**17**) (399.87 MHz for ^{1}H, D_2O, 30 °C).

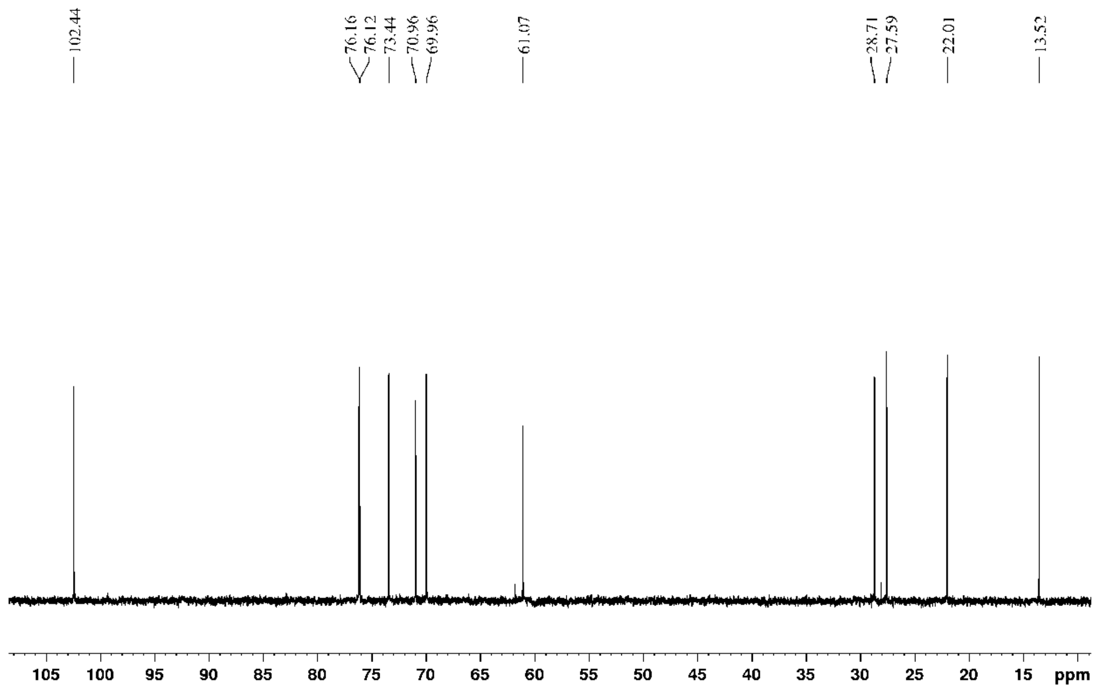

Figure S15B. ^{13}C NMR spectrum of pentyl glucoside (**17**) (100.55 MHz for ^{13}C, D_2O, 30 °C).

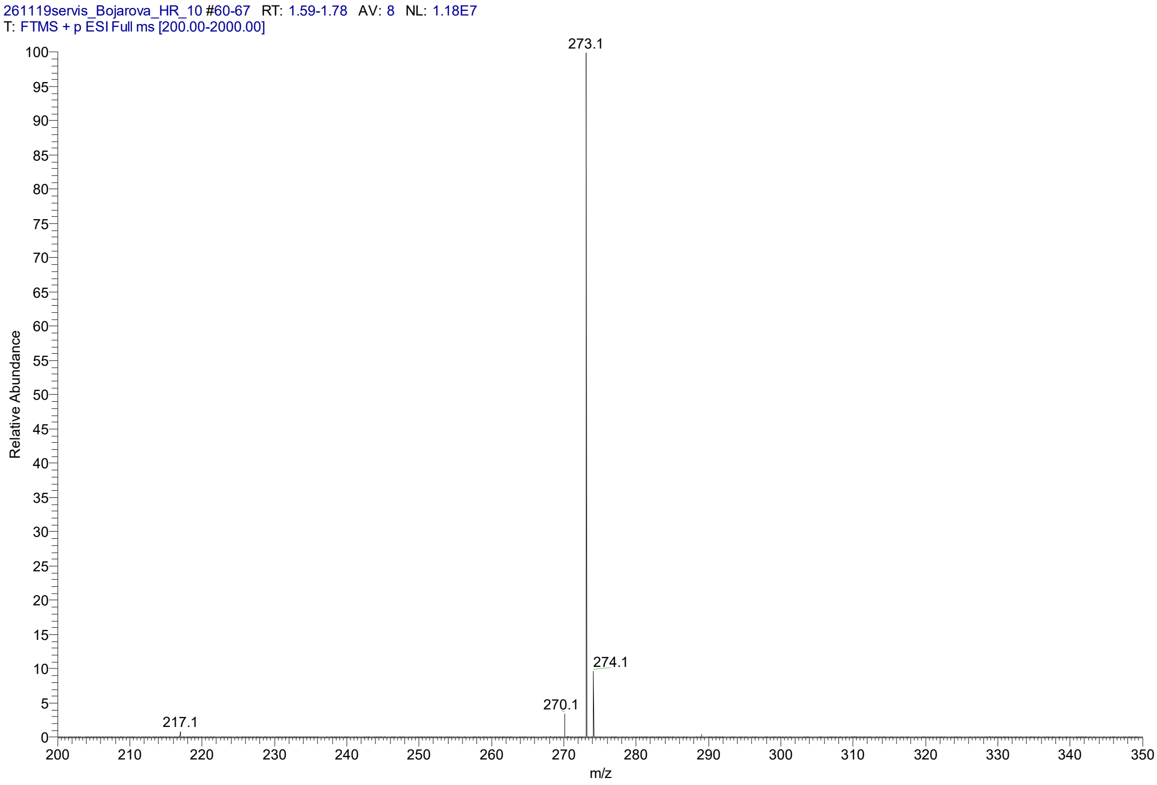

Figure S15C. ESI^+ MS spectrum of pentyl glucoside (**17**) ($[M + Na]^+$, *m/z* 273.1).

Table S7. ^{1}H and ^{13}C NMR data of β-glucosyl azide (**18**) (399.87 MHz for ^{1}H, 100.55 MHz for ^{13}C, D_2O, 30 °C).

	**Atom**	δ_C	**m.**	δ_H	**n**$_H$	**m.**	***J*[Hz]**
**Glc**	**1**	90.34	D	4.734	1	d	8.8
	**2**	73.10	D	3.268	1	dd	9.2, 8.8
	**3**	76.00	D	3.517	1	dd	9.2, 9.1
	**4**	69.45	D	3.417	1	dd	9.8, 9.1
	**5**	78.15	D	3.538	1	ddd	9.8, 5.6, 2.2
	**6**	60.83	T	3.918	1	dd	12.4, 2.2
				3.743	1	dd	12.4, 5.6

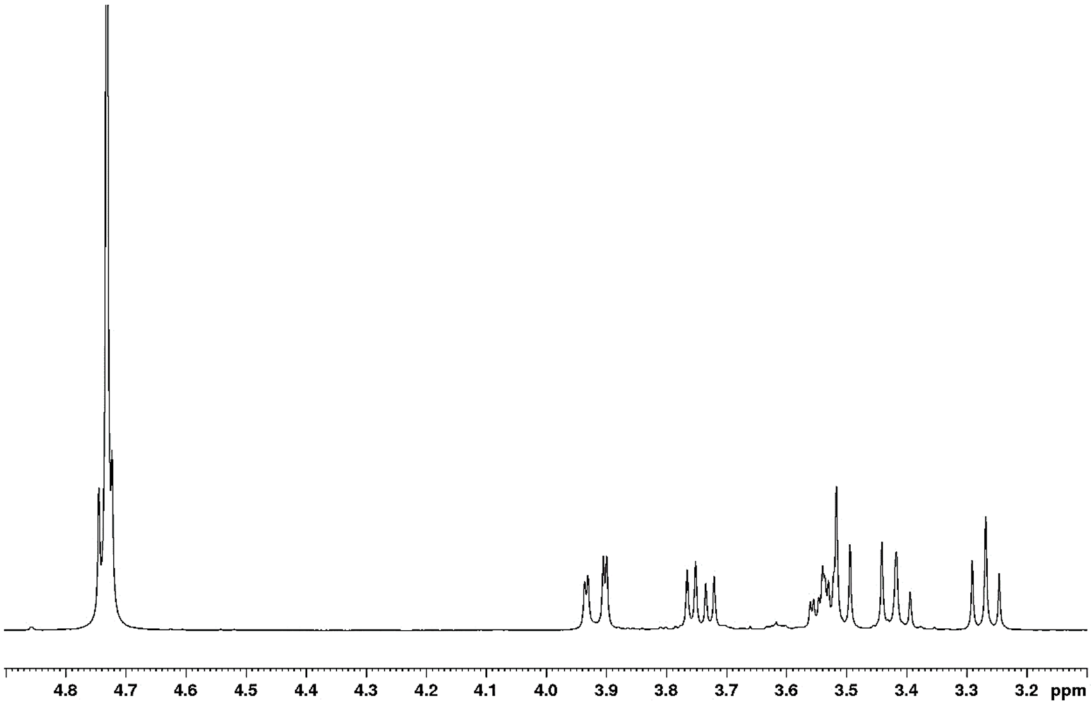

Figure S16A. ^{1}H NMR spectrum β-glucosyl azide (**18**) (399.87 MHz for ^{1}H, D_2O, 30 °C).

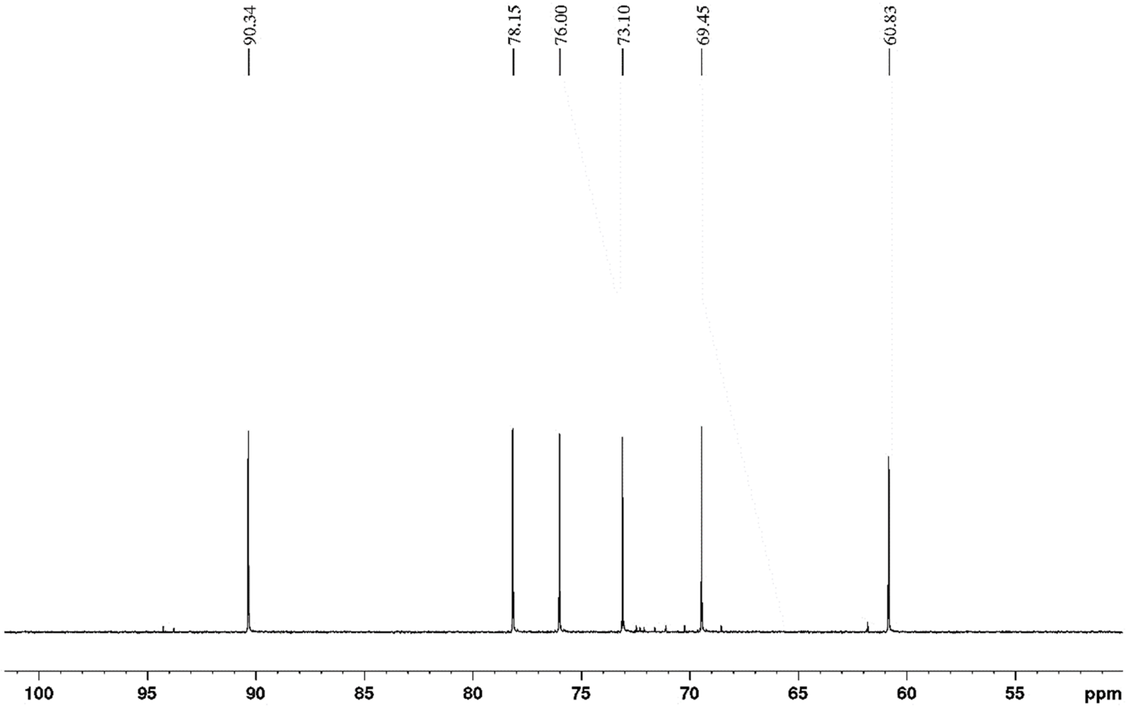

Figure S16B. ^{13}C NMR spectrum of β-glucosyl azide (**18**) (100.55 MHz for ^{13}C, D_2O, 30 °C).

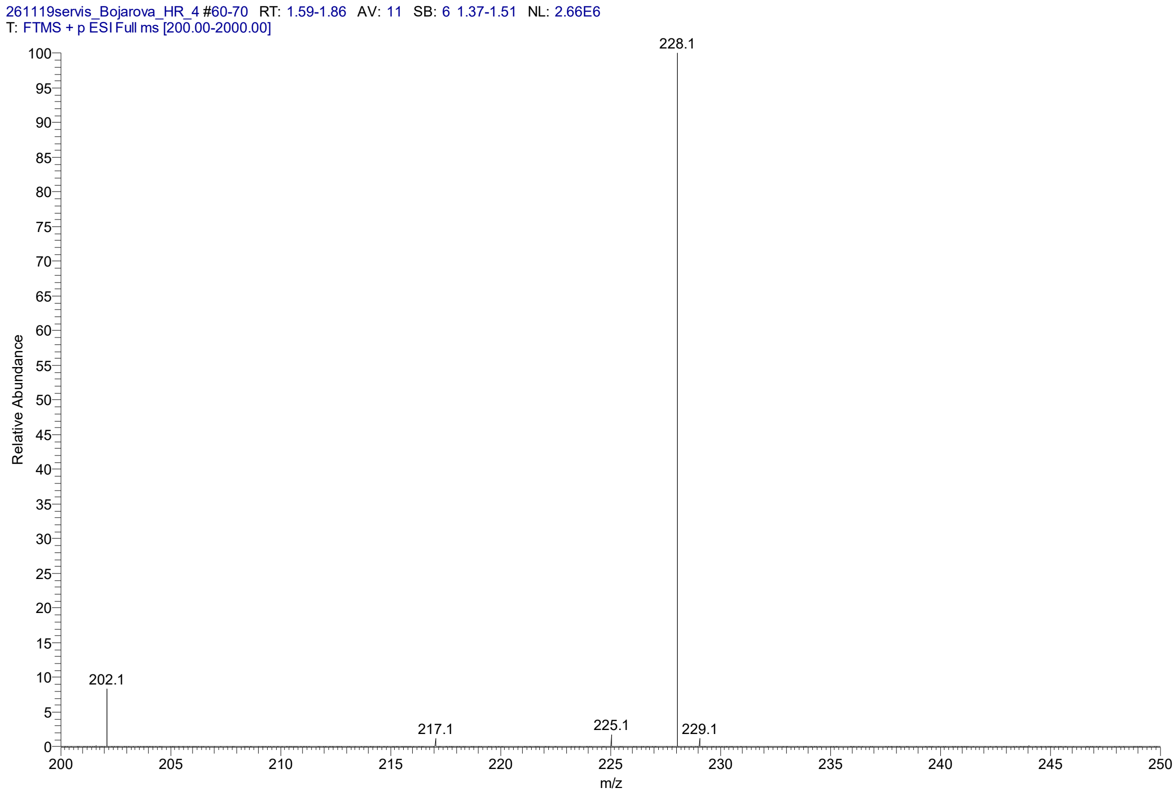

Figure S16C. ESI$^+$ MS spectrum of β-glucosyl azide (**18**) ([M + Na]$^+$, *m/z* 228.1).

Table S8. ^{1}H and ^{13}C NMR data of 2-azidoethyl glucoside (**19**) (399.87 MHz for ^{1}H, 100.55 MHz for ^{13}C, D_2O, 30 °C).

	Atom	δ_C	m.	δ_H	n_H	m.	*J*[Hz]
**Glc**	**1**	102.60	D	4.511	1	d	7.9
	**2**	73.36	D	3.305	1	dd	9.3, 7.9
	**3**	75.99	D	3.508	1	dd	9.3, 9.0
	**4**	69.90	D	3.400	1	dd	9.7, 9.0
	**5**	76.24	D	3.474	1	ddd	9.7, 5.9, 2.2
	**6**	61.02	T	3.930	1	dd	12.3, 2.2
				3.734	1	dd	12.3, 5.9
**aglycone**	**1′**	68.74	T	4.061	1	dt	11.4, 4.9
				3.858	1	dt	11.4, 5.1
	**2′**	50.82	T	3.565	2	dd	5.1, 4.9

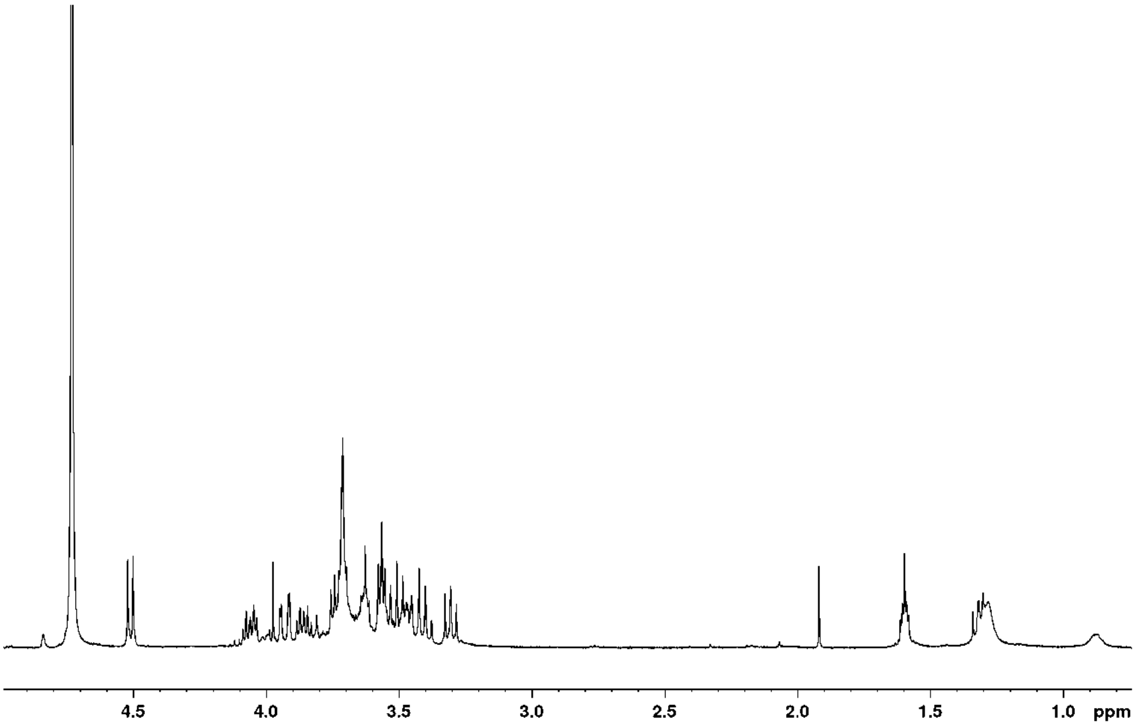

Figure S17A. ^{1}H NMR spectrum of 2-azidoethyl glucoside (**19**) (399.87 MHz for ^{1}H, D_2O, 30 °C).

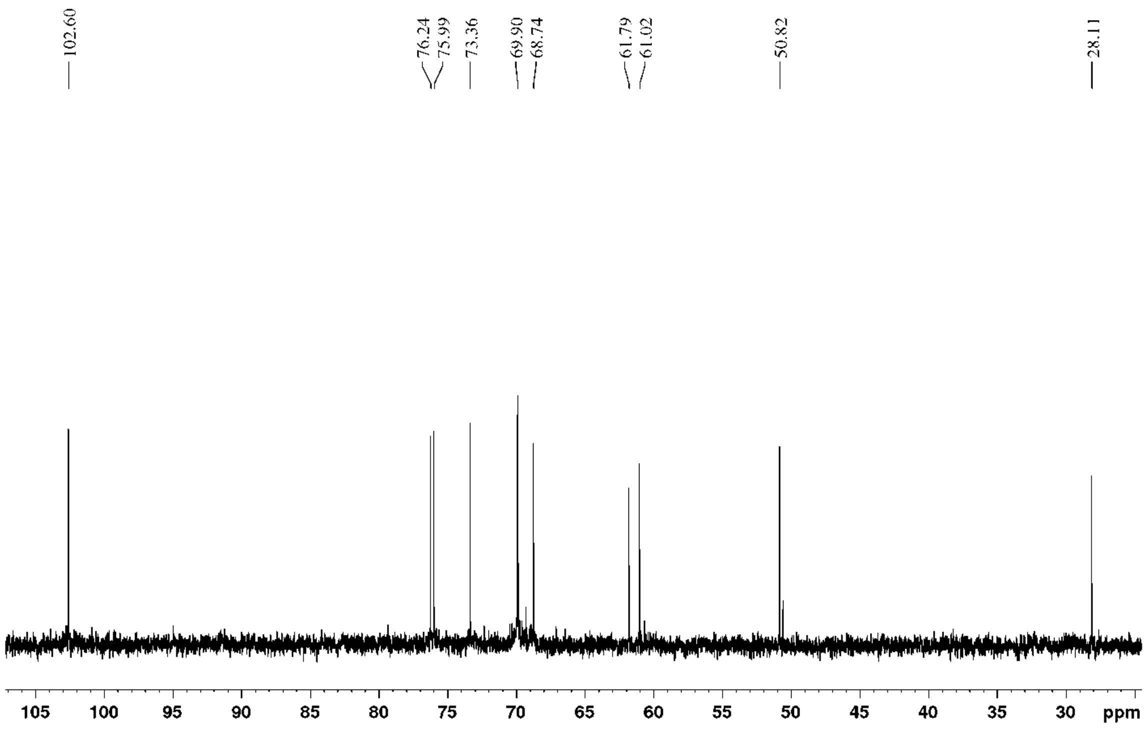

Figure S17B. ^{13}C NMR spectrum of 2-azidoethyl glucoside (**19**) (100.55 MHz for ^{13}C, D_2O, 30 °C).

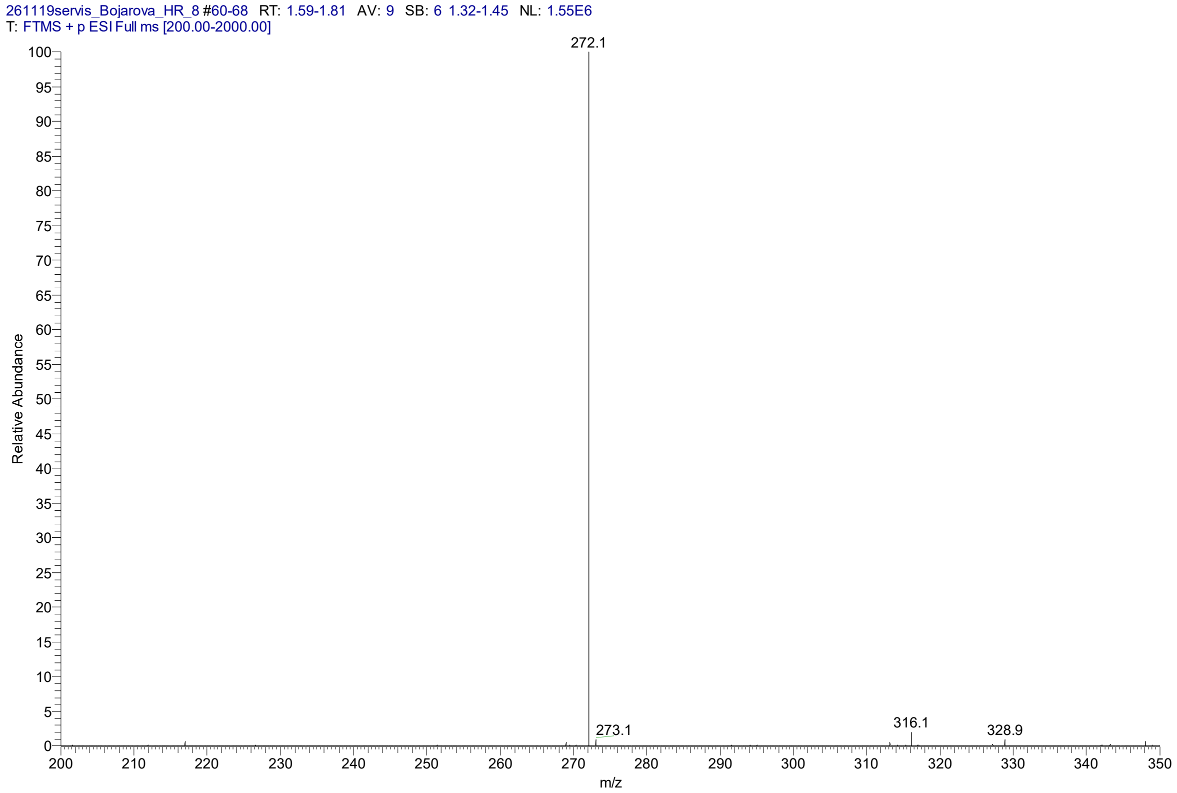

Figure S17C. ESI+ MS spectrum of 2-azidoethyl glucoside (**19**) ($[M + Na]^+$, *m/z* 272.1).

Table S9. ^{1}H and ^{13}C NMR data of 2-phenylethyl glucoside (**20**) (399.87 MHz for ^{1}H, 100.55 MHz for ^{13}C, D_2O, 30 °C).

	Atom	δ_C	m.	δ_H	n_H	m.	*J*[Hz]
**Glc**	**1**	102.47	D	4.471	1	d	8.0
	**2**	73.36	D	3.244	1	dd	9.3, 8.0
	**3**	76.02	D	3.472	1	dd	9.3, 8.8
	**4**	69.91	D	3.372	1	dd	9.7, 8.8
	**5**	76.16	D	3.474	1	ddd	9.7, 5.9, 2.2
	**6**	61.03	T	3.907	1	dd	12.3, 2.2
				3.713	1	dd	12.3, 5.7
**aglycone**	**1'**	71.01	T	4.160	1	dt	10.1, 6.9
				3.938	1	dt	10.1, 6.9
	**2'**	35.46	T	2.980	2	dd	6.9, 6.9
	***i*-**	139.02	S	-	0		
	***o*-**	129.29	D	7.37[a]	2	m	
	***m*-**	128.94	D	7.39[a]	2	m	
	***p*-**	126.82	D	7.311	1	m	

[a] … HSQC readout.

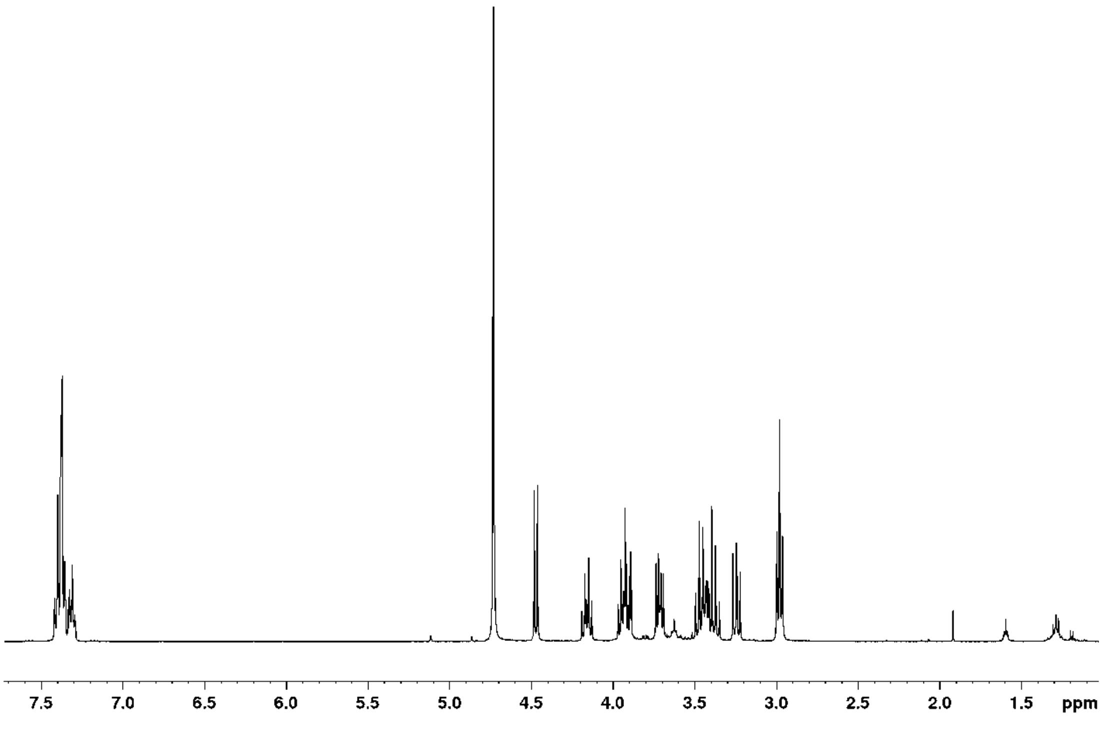

Figure S18A. ^{1}H NMR spectrum of 2-phenylethyl glucoside (**20**) (399.87 MHz for ^{1}H, D_2O, 30 °C).

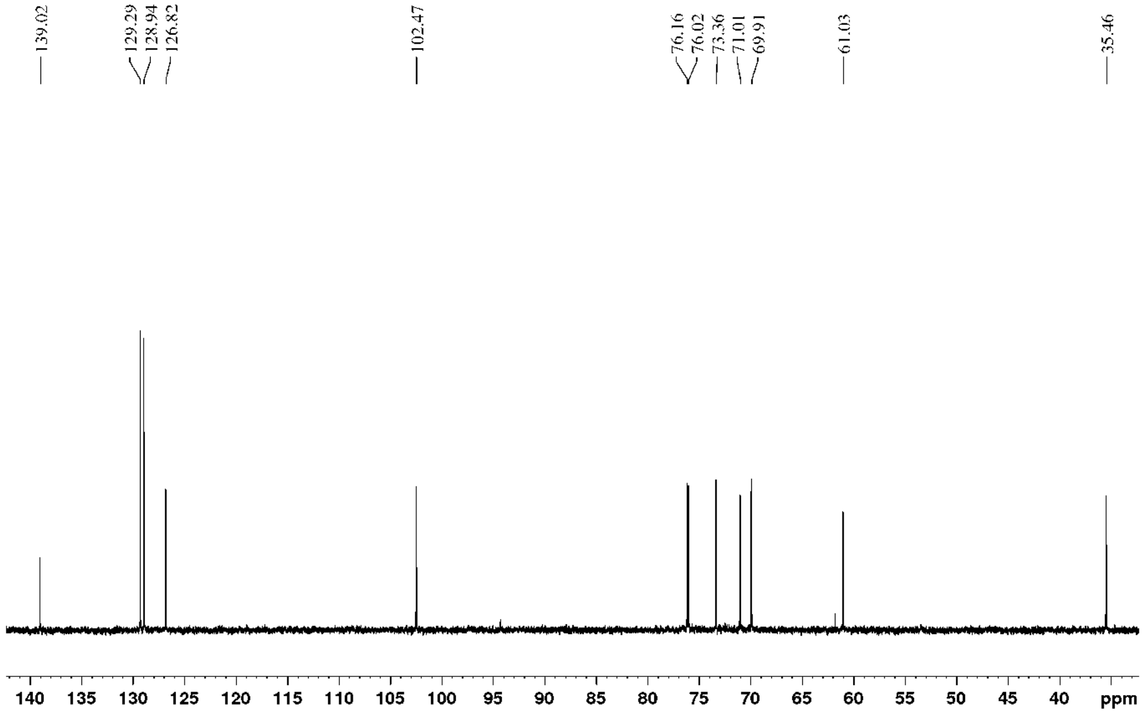

Figure S18B. ^{13}C NMR spectrum of 2-phenylethyl glucoside (**20**) (100.55 MHz for ^{13}C, D_2O, 30 °C).

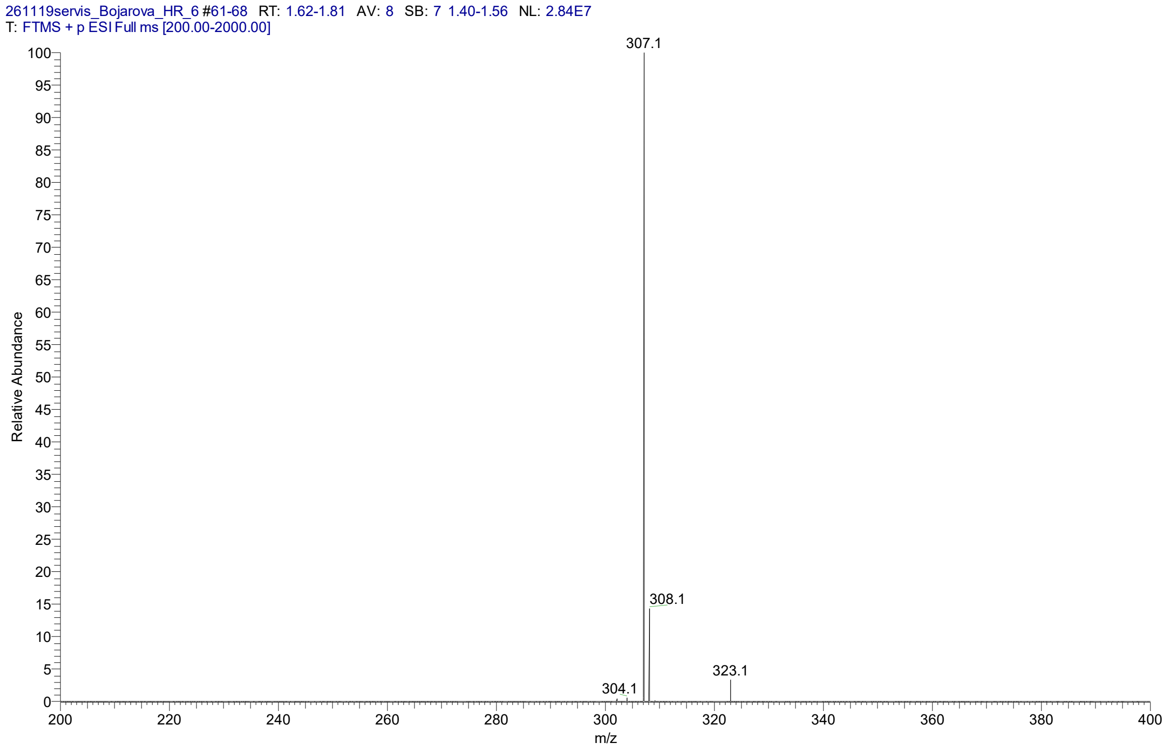

Figure S18C. ESI$^+$ MS spectrum of 2-phenylethyl glucoside (**20**) ([M + Na]$^+$, *m/z* 307.1).

Table S10. ^{1}H and ^{13}C NMR data of catechol glucoside (**21**) (399.87 MHz for ^{1}H, 100.55 MHz for ^{13}C, CD_3OD, 30 °C).

	**Atom**	δ_C	**m.**	δ_H	n_H	**m.**	***J*[Hz]**
**Glc**	**1**	104.58	D	4.742	1	d	7.6
	**2**	74.97	D	3.492	1	dd	9.3, 7.6
	**3**	77.71	D	3.453	1	m	
	**4**	71.39	D	3.40[a]	1	m	
	**5**	78.38	D	3.40[a]	1	m	
	**6**	62.51	T	3.890	1	dd	12.0, 2.0
				3.719	1	m	
**aglycon**	**1′**	146.93	S	-	0		
	**2′**	148.62	S	-	0		
	**3′**	117.20	D	6.834	1	dd	8.0, 1.6
	**4′**	124.93	D	6.903	1	ddd	8.0, 7.4, 1.5
	**5′**	121.05	D	6.769	1	ddd	8.1, 7.4, 1.6
	**6′**	119.15	D	7.178	1	dd	8.1, 1.5

[a] … HSQC readout.

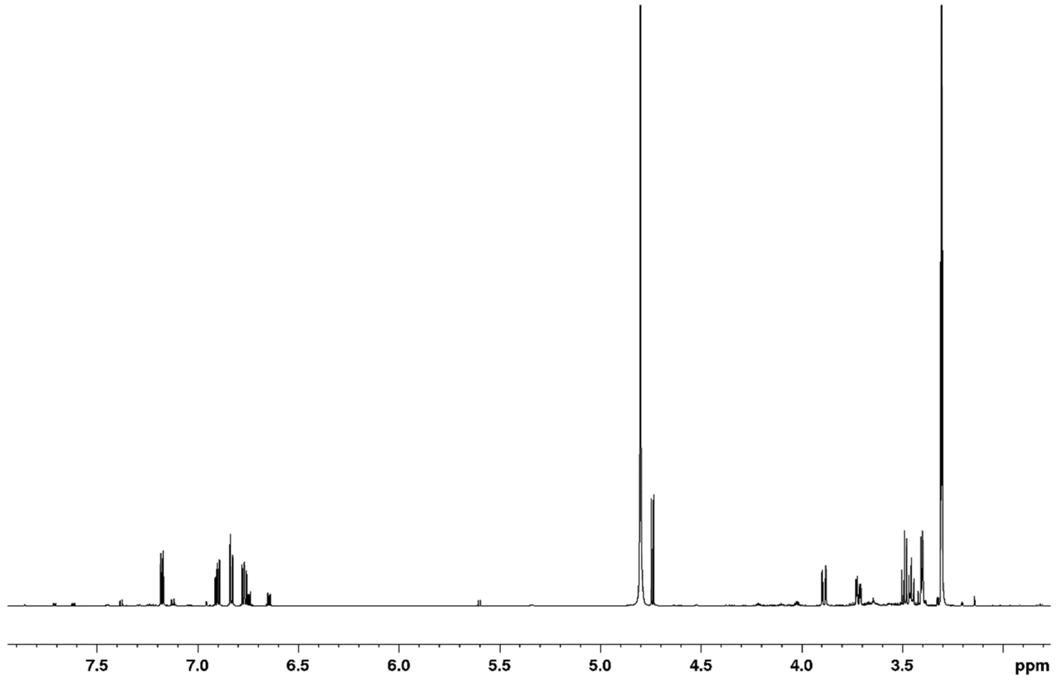

Figure S19A. ^{1}H NMR spectrum of catechol glucoside (**21**) (399.87 MHz for ^{1}H, CD_3OD, 30 °C).

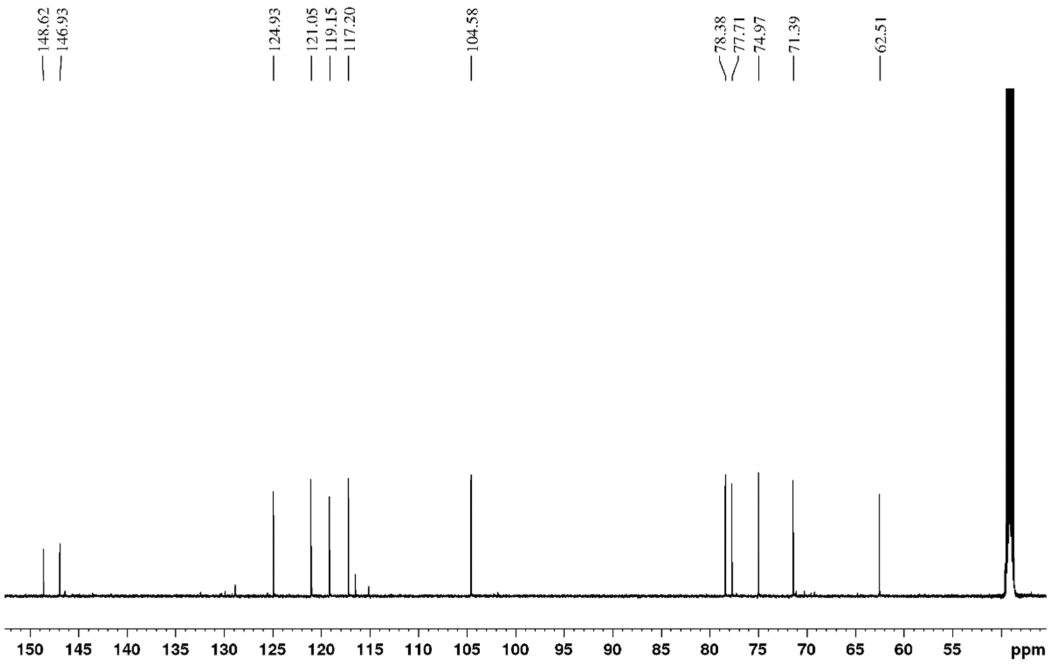

Figure S19B. ^{13}C NMR spectrum of catechol glucoside (**21**) (100.55 MHz for ^{13}C, CD_3OD, 30 °C).

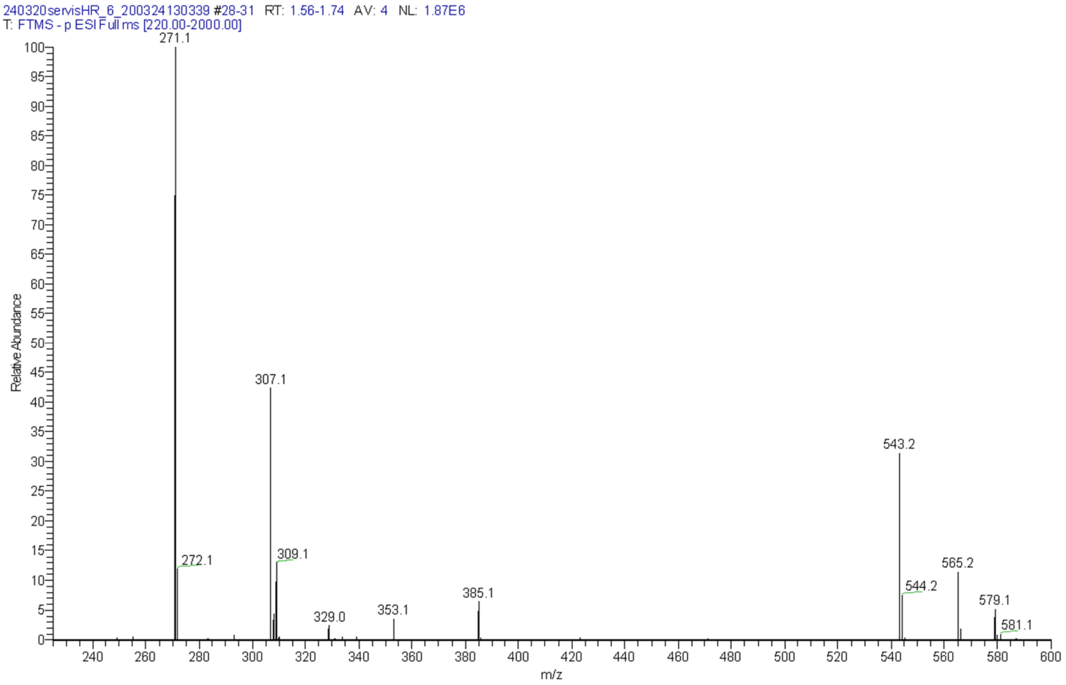

Figure S19C. ESI$^-$ MS spectrum of catechol glucoside (**21**) ([M − H]$^-$, *m*/*z* 271.1).

Table S11. ^{1}H and ^{13}C NMR data of *p*-nitrophenyl glucoside (**22**) (600.23 MHz for ^{1}H, 150.93 MHz for ^{13}C, D_2O, 30 °C).

	Atom	δ_C	m.	δ_H	n_H	m.	*J* [Hz]
**Glc**	**1**	99.88	D	5.395	1	m	
	**2**	73.15	D	3.763	1	m	
	**3**	75.83	D	3.763	1	m	
	**4**	69.72	D	3.649	1	m	
	**5**	76.67	D	3.821	1	ddd	9.8, 5.7, 2.3
	**6**	60.88	T	4.068	1	dd	12.4, 2.3
				3.889	1	dd	12.4, 5.7
**aglycone**	***ipso-***	162.10	S	-	0		
	ortho-	116.94	D	7.386	2	m	
	meta-	126.48	D	8.399	2	m	
	para-	143.05	S	-	0		

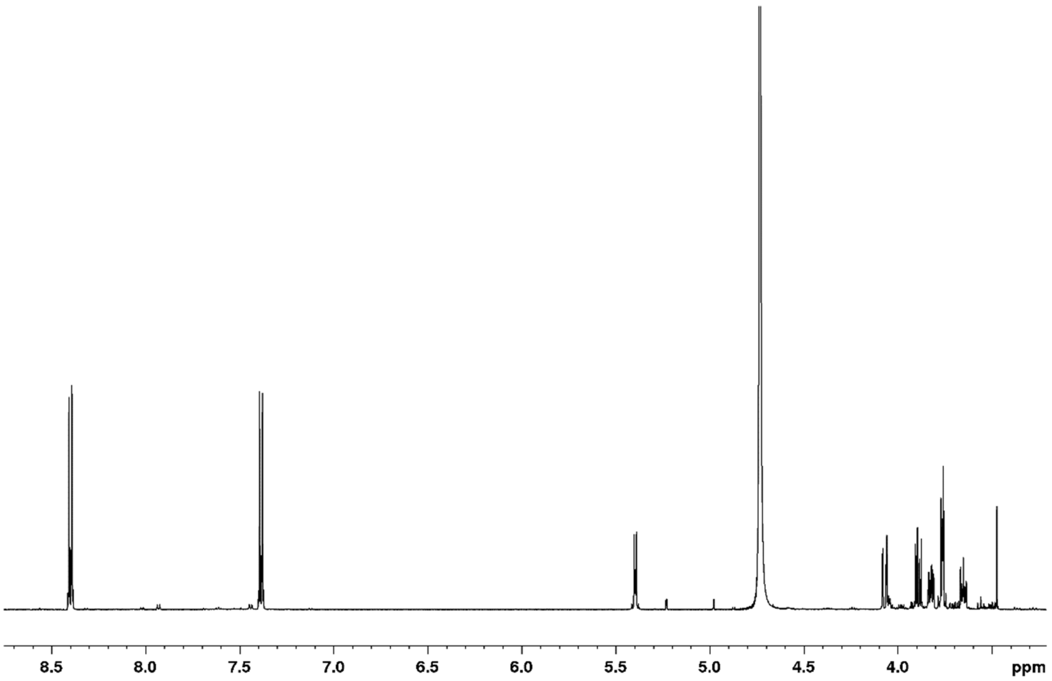

Figure S20A. ^{1}H NMR spectrum of *p*-nitrophenyl glucoside (**22**) (600.23 MHz for ^{1}H, D_2O, 30 °C).

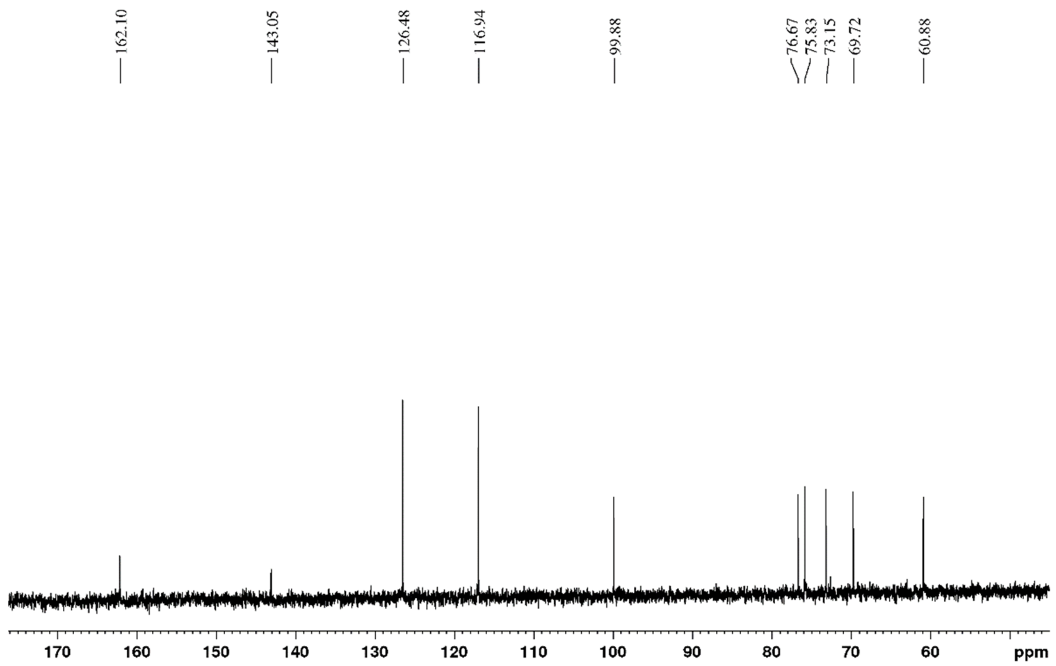

Figure S20B. ^{13}C NMR spectrum of *p*-nitrophenyl glucoside (**22**) (150.93 MHz for ^{13}C, D_2O, 30 °C).

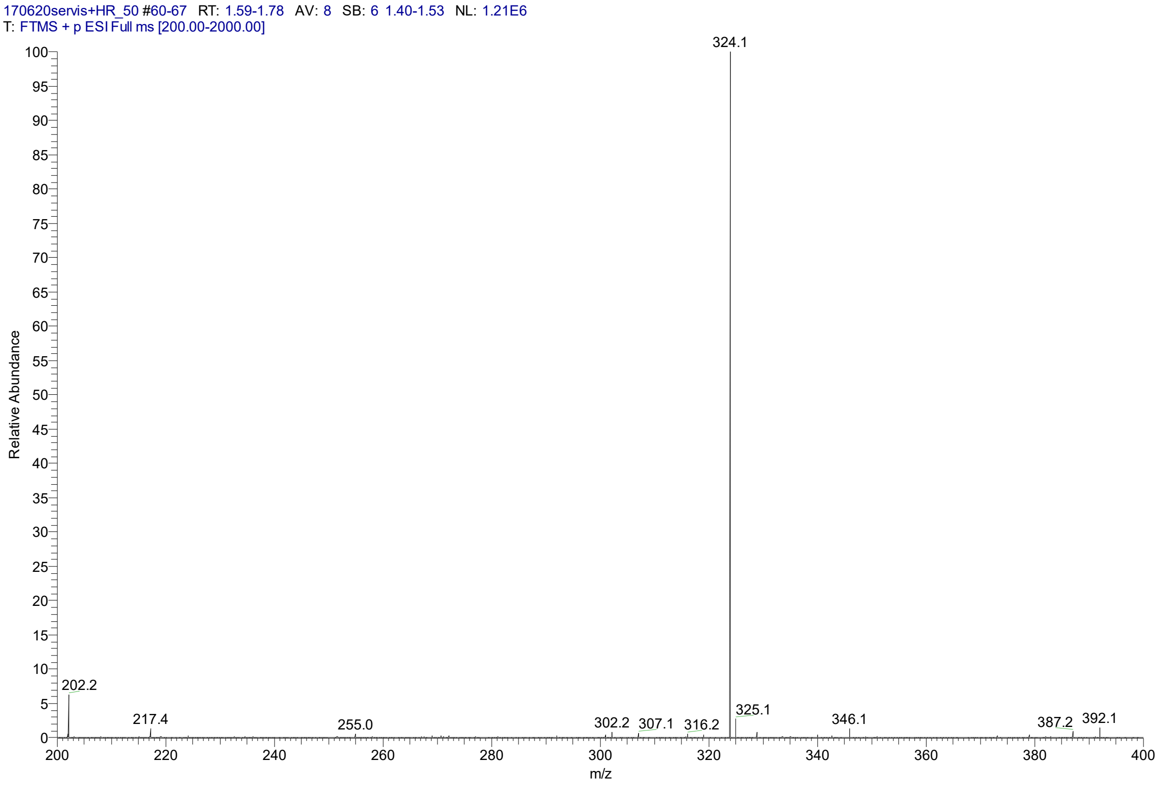

Figure S20C. ESI^+ MS spectrum of *p*-nitrophenyl glucoside (**22**) ($[M + Na]^+$, *m/z* 324.1).

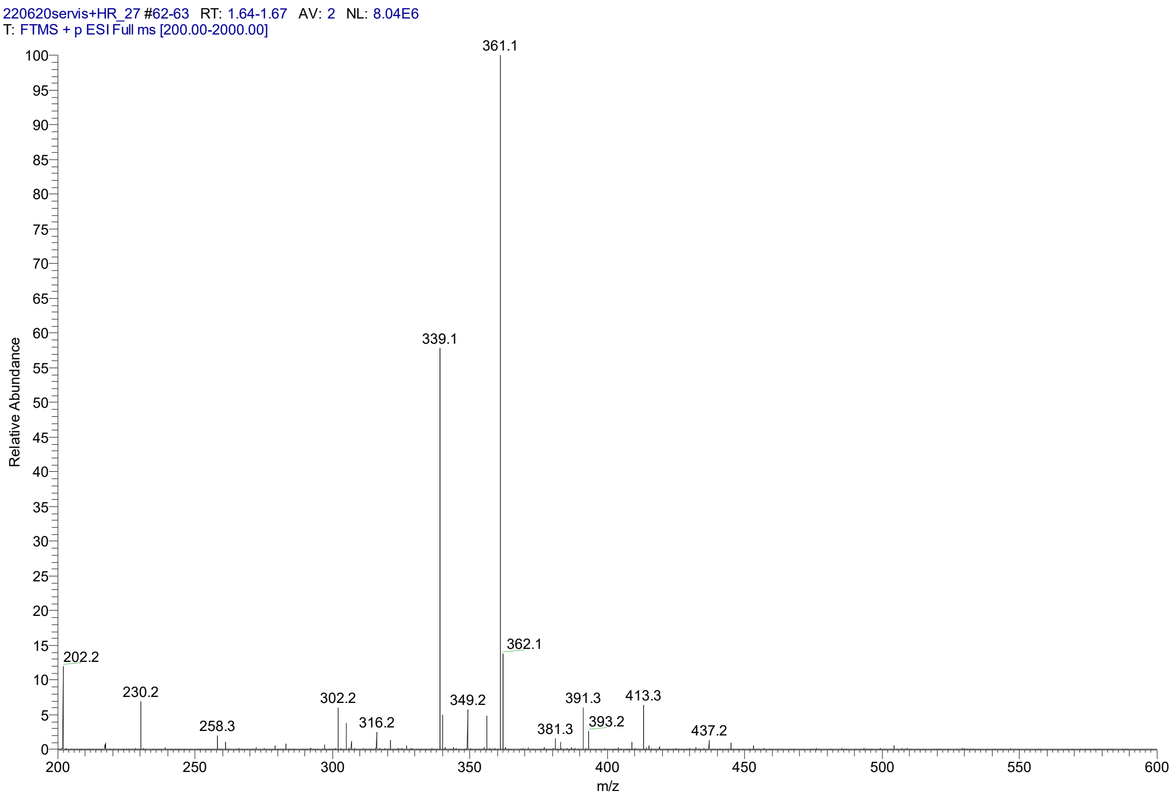

Figure S21. ESI⁺ MS spectrum of 4-methylumbelliferyl glucoside (**23**) ($[M + H]^+$, *m/z* 339.1; $[M + Na]^+$, *m/z* 361.1).

Table S12. ^1H, ^{13}C and ^{15}N NMR data of 1,2,3-*tris*-[1-(α-L-rhamnopyranosyl-(1→6)-β-D-glucopyranosyl)-1*H*-1,2,3-triazol-4-yl)-2-ethyloxy]- benzene (**25**) (700.13 MHz for ^1H, 176.05 MHz for ^{13}C, 70.94 MHz for ^{15}N, D_2O, 30 °C).

	Atom	δ_C	m.	δ_N	δ_H	n_H	m.	*J*[Hz]
**Rhamnose** °	**1**	100.85	D	-	4.780	2	d	1.8
	**2**	70.33	D	-	3.951	2	dd	3.5, 1.8
	**3**	70.54	D	-	3.769	2	dd	9.7, 3.5
	**4**	72.32	D	-	3.437	2	dd	9.7, 9.6
	**5**	68.95	D	-	3.695	2	dq	9.6, 6.3
	**6**	16.91	Q	-	1.257	6	d	6.3
**Glucose** °	**1**	102.78	D	-	4.408	2	d	7.9
	**2**	73.20	D	-	3.214	2	dd	9.4, 7.9
	**3**	75.68	D	-	3.450	2	dd	9.4, 9.0
	**4**	70.00	D	-	3.336	2	dd	9.9, 9.0
	**5**	75.06	D	-	3.517	2	ddd	9.9, 6.0, 2.0
	**6**	67.19	T	-	3.953	2	dd	11.7, 2.0
					3.600	2	dd	11.7, 6.0
**Spacer** °	**1**	62.26	T	-	5.220	4	s	
	**2**	143.5	S	-	-	0		
	**3**	126.22	D	-	8.120	2	s	
	**4**	-		256	-	0		
	**5**	-		n.d.	-	0		
	**6**	-		n.d.	-	0		
	**7**	50.70	T	-	4.277	2	m	
					4.126	2	m	
	**8**	68.40	T	-	4.710	4	m	
**Rhamnose** ⁱ	**1**	100.88	D	-	4.775	1	d	1.8

	**2**	70.33	D	-	3.934	1	dd	3.5, 1.8
	**3**	70.54	D	-	3.749	1	dd	9.8, 3.5
	**4**	72.32	D	-	3.435	1	dd	9.8, 9.6
	**5**	68.95	D	-	3.695	1	dq	9.6, 6.3
	**6**	16.91	Q	-	1.257	3	d	6.3
**Glucose** [i]	**1**	102.87	D	-	4.400	1	d	7.9
	**2**	73.20	D	-	3.214	1	dd	9.4, 7.9
	**3**	75.68	D	-	3.464	1	dd	9.4, 9.1
	**4**	70.08	D	-	3.340	1	dd	9.9, 9.1
	**5**	75.06	D	-	3.535	1	ddd	9.9, 4.8, 2.0
	**6**	67.29	T	-	3.965	1	dd	11.7, 2.0
					3.590	1	dd	11.7, 4.8
**Spacer** [i]	**1**	65.29	T	-	5.087	2	s	
	**2**	143.3	S	-	-	0		
	**3**	126.51	D	-	7.830	1	s	
	**4**	-		245	-	0		
	**5**	-		n.d.	-	0		
	**6**	-		342	-	0		
	**7**	50.54	T	-	4.194	1	m	
					4.020	1	m	
	**8**	68.54	T	-	4.587	2	m	
**aromatics part**	***i-***	135.9[a]	S	-	-	0		
	o-	152.00	S	-	-	0		
	m-	108.80	D	-	6.902	2	d	8.4
	p-	125.55	D	-	7.179	1	t	8.4

[a] HSQC readout; [o,i] substituent in *ortho*/*ipso* position at aromatic ring; n.d. not detected.

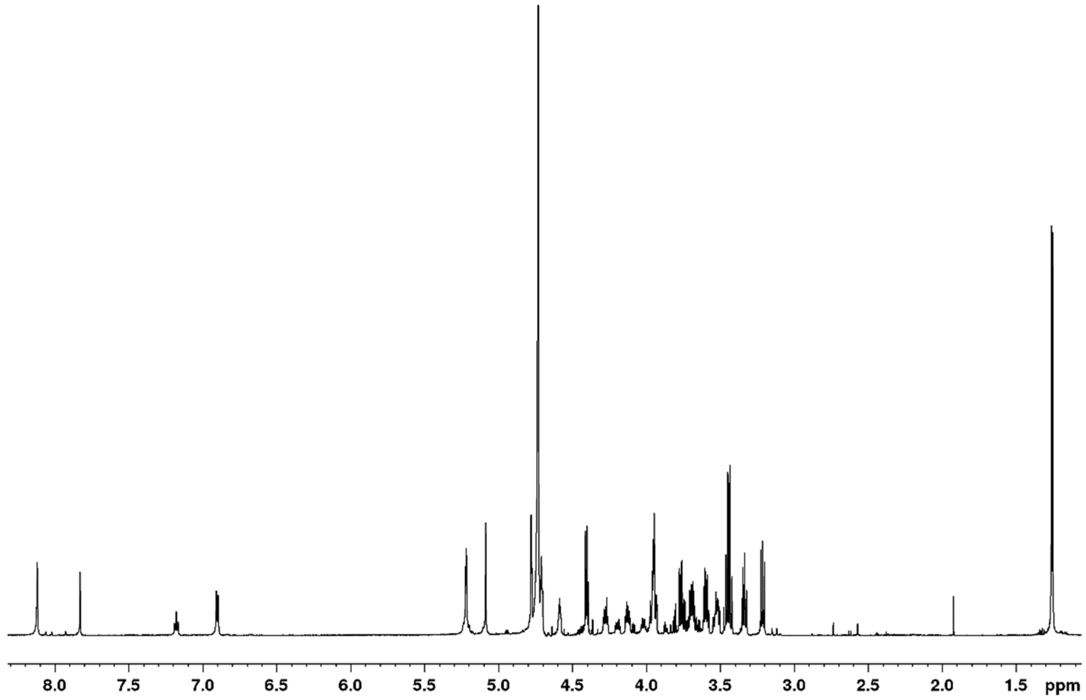

Figure S22A. ^{1}H NMR spectrum of 1,2,3-*tris*-[1-(α-L-rhamnopyranosyl-(1→6)-β-D-glucopyranosyl)-1*H*-1,2,3-triazol-4-yl)-2-ethyloxy]benzene (**25**) (700.13 MHz for ^{1}H, D_2O, 30 °C).

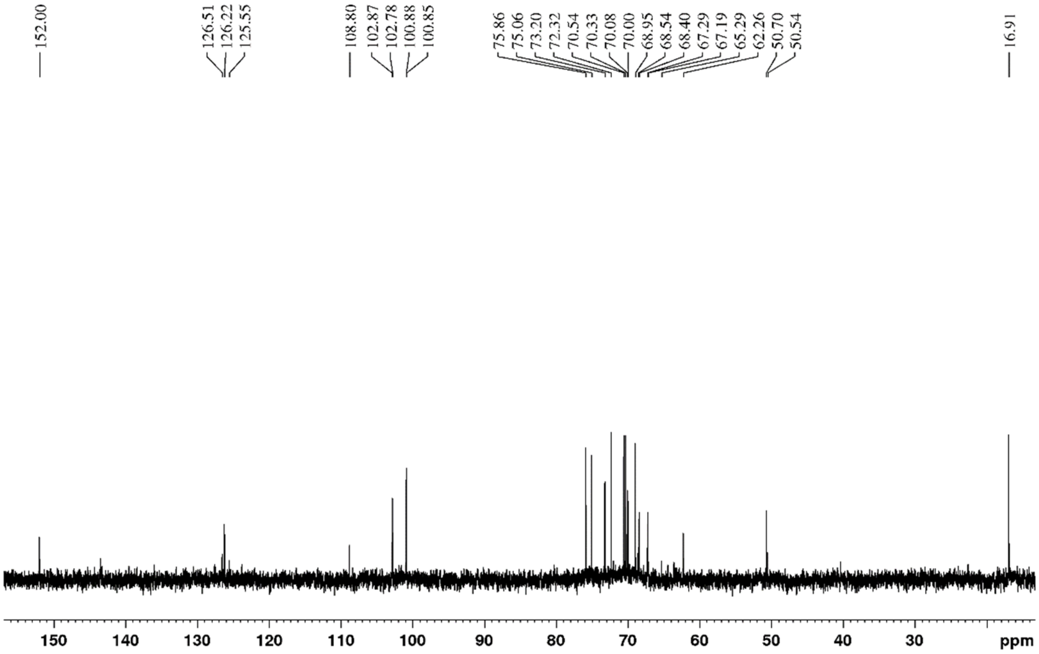

Figure S22B. ^{13}C NMR spectrum of 1,2,3-*tris*-[1-(α-L-rhamnopyranosyl-(1→6)-β-D-glucopyranosyl)-1*H*-1,2,3-triazol-4-yl)-2-ethyloxy]benzene (**25**) (176.05 MHz for ^{13}C, D_2O, 30 °C).

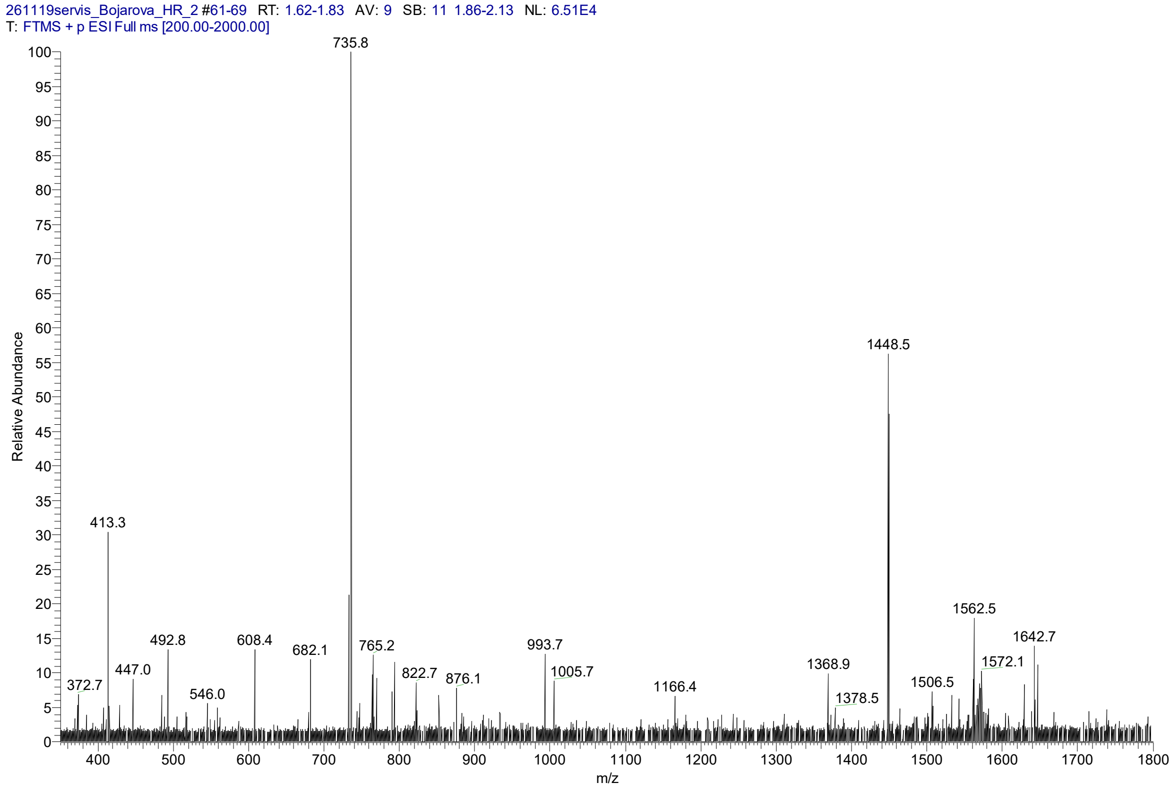

Figure S22C. ESI^{+} MS spectrum of 1,2,3-*tris*-[1-(α-L-rhamnopyranosyl-(1→6)-β-D-glucopyranosyl)-1*H*-1,2,3-triazol-4-yl)-2-ethyloxy]benzene (**25**) ([M + Na]$^{+}$, *m*/*z* 1448.5; (M + 2Na]$^{2+}$, *m*/*z* 735.8).
